# PI(5)P Regulates Autophagosome Biogenesis

**DOI:** 10.1016/j.molcel.2014.12.007

**Published:** 2015-01-22

**Authors:** Mariella Vicinanza, Viktor I. Korolchuk, Avraham Ashkenazi, Claudia Puri, Fiona M. Menzies, Jonathan H. Clarke, David C. Rubinsztein

**Affiliations:** 1 Department of Medical Genetics, Cambridge Institute for Medical Research, Wellcome/MRC Building, Cambridge Biomedical Campus, Hills Road, Cambridge CB2 0XY, UK; 2 Department of Pharmacology, University of Cambridge, Tennis Court Road, Cambridge CB2 1PD, UK

## Abstract

Phosphatidylinositol 3-phosphate (PI(3)P), the product of class III PI3K VPS34, recruits specific autophagic effectors, like WIPI2, during the initial steps of autophagosome biogenesis and thereby regulates canonical autophagy. However, mammalian cells can produce autophagosomes through enigmatic noncanonical VPS34-independent pathways. Here we show that PI(5)P can regulate autophagy via PI(3)P effectors and thereby identify a mechanistic explanation for forms of noncanonical autophagy. PI(5)P synthesis by the phosphatidylinositol 5-kinase PIKfyve was required for autophagosome biogenesis, and it increased levels of PI(5)P, stimulated autophagy, and reduced the levels of autophagic substrates. Inactivation of VPS34 impaired recruitment of WIPI2 and DFCP1 to autophagic precursors, reduced ATG5-ATG12 conjugation, and compromised autophagosome formation. However, these phenotypes were rescued by PI(5)P in VPS34-inactivated cells. These findings provide a mechanistic framework for alternative VPS34-independent autophagy-initiating pathways, like glucose starvation, and unravel a cytoplasmic function for PI(5)P, which previously has been linked predominantly to nuclear roles.

## Highlights

• PI(5)P positively regulates autophagy
• PI(5)P is associated with autophagy effectors that bind PI(3)P
• PI(5)P sustains noncanonical autophagy in PI(3)P-depleted cells
• PI(5)P is essential for VPS34-independent, glucose-starvation-induced autophagy

## Graphical Abstract

**Figure:**
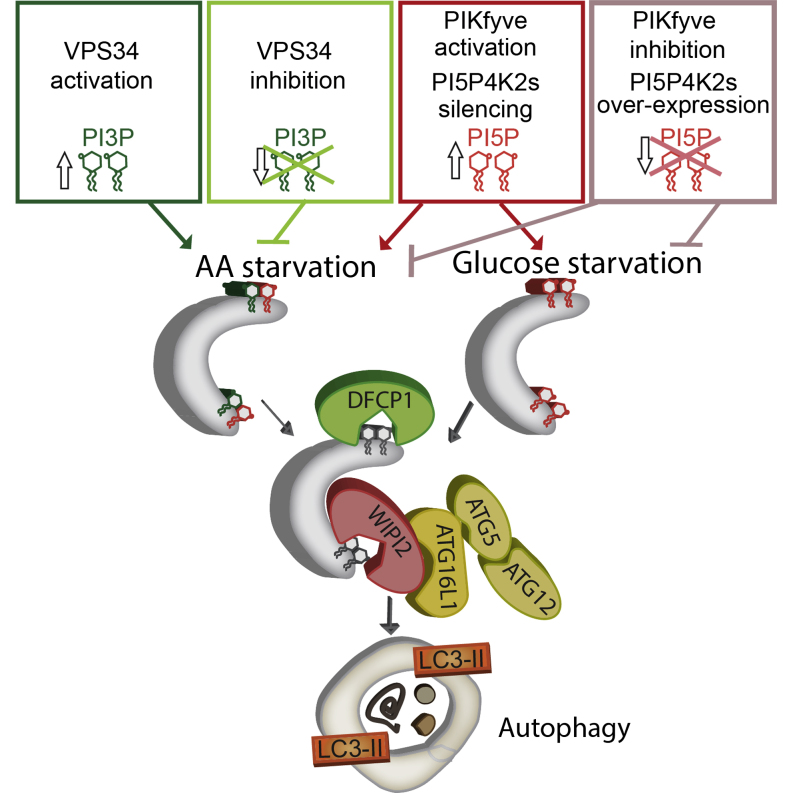

## Teaser

PI(3)P, the product of VPS34, regulates canonical autophagy; however, mammalian cells can produce autophagosomes through enigmatic noncanonical VPS34-independent pathways. Vicinanza et al. show that PI(5)P can regulate autophagy, even in cells where VPS34 is compromised and acts via PI(3)P effectors. This provides a mechanistic explanation for forms of noncanonical autophagy.

## Introduction

Macroautophagy (henceforth autophagy) is a cellular process that delivers damaged organelles, invasive bacteria, and long-lived or aggregate-prone proteins to lysosomes for degradation (Boya et al., 2013). These substrates are engulfed along with bulk cytoplasm by double-membraned, cup-shaped phagophores, which form autophagosomes after their edges extend and fuse. After autophagosome-lysosome fusion, the resulting degradation products are recycled back to the cytosol and are reused to enhance cell survival during nutrient deprivation. Autophagy impacts the pathogenesis of diverse diseases, including neurodegenerative conditions, cancers, and infectious diseases (Nixon, 2013).

Many proteins (ATGs) regulate the membrane remodelling and trafficking events in autophagy, but comparatively little is known about the roles of lipids and their metabolizing enzymes in this process (Nakatogawa et al., 2009). Phosphoinositides (PIs) are low-abundance lipids that are interconverted by highly regulated sets of PI kinases and phosphatases. The interconvertibility of PIs enables rapid local changes in the identity of intracellular compartments to dynamically recruit effector proteins to specific membranes at the right time. The class III phosphatidylinositol 3-kinase (also known as VPS34) and its product phosphatidylinositol 3-phosphate (PI(3)P) are critical for autophagosome formation (Boya et al., 2013; Nakatogawa et al., 2009). Local increases of PI(3)P recruits proteins associated with autophagy initiation, such as DFCP1 (double FYVE-containing protein 1) and the WIPI proteins (WD-repeat protein interacting with PI), of which WIPI2 has been characterized as an effector of autophagy (Polson et al., 2010).

While autophagy has been classically considered to be PI(3)P-dependent, noncanonical VPS34-independent autophagy has been reported (Codogno et al., 2012; Plowey et al., 2008; Scarlatti et al., 2008; Zhu et al., 2007), as autophagosomes are seen in T lymphocytes and sensory neurons from *Vps34*^−/−^ mice (Zhou et al., 2010) and in glucose-starved cells treated with the VPS34 inhibitor Wortmannin (Wm) (McAlpine et al., 2013). However, it is unclear whether these phenomena may be explicable by VPS34-independent source(s) of PI(3)P (Devereaux et al., 2013) or by other PIs.

PI(5)P remains the most enigmatic of the PIs due to its low abundance (Shisheva, 2013). The pathways regulated by PI(5)P in mammalian cells and its effectors/binding proteins are still poorly understood. So far these include chromatin organization in the nuclei, bacteria invasion, and cytoskeletal remodelling (Shisheva, 2013). Here, we describe a role for PI(5)P as a regulator of autophagosome biogenesis that can also regulate autophagy when VPS34 is inhibited.

## Results

### PI(5)P Regulates Autophagosome Biogenesis

**Figure 1:**
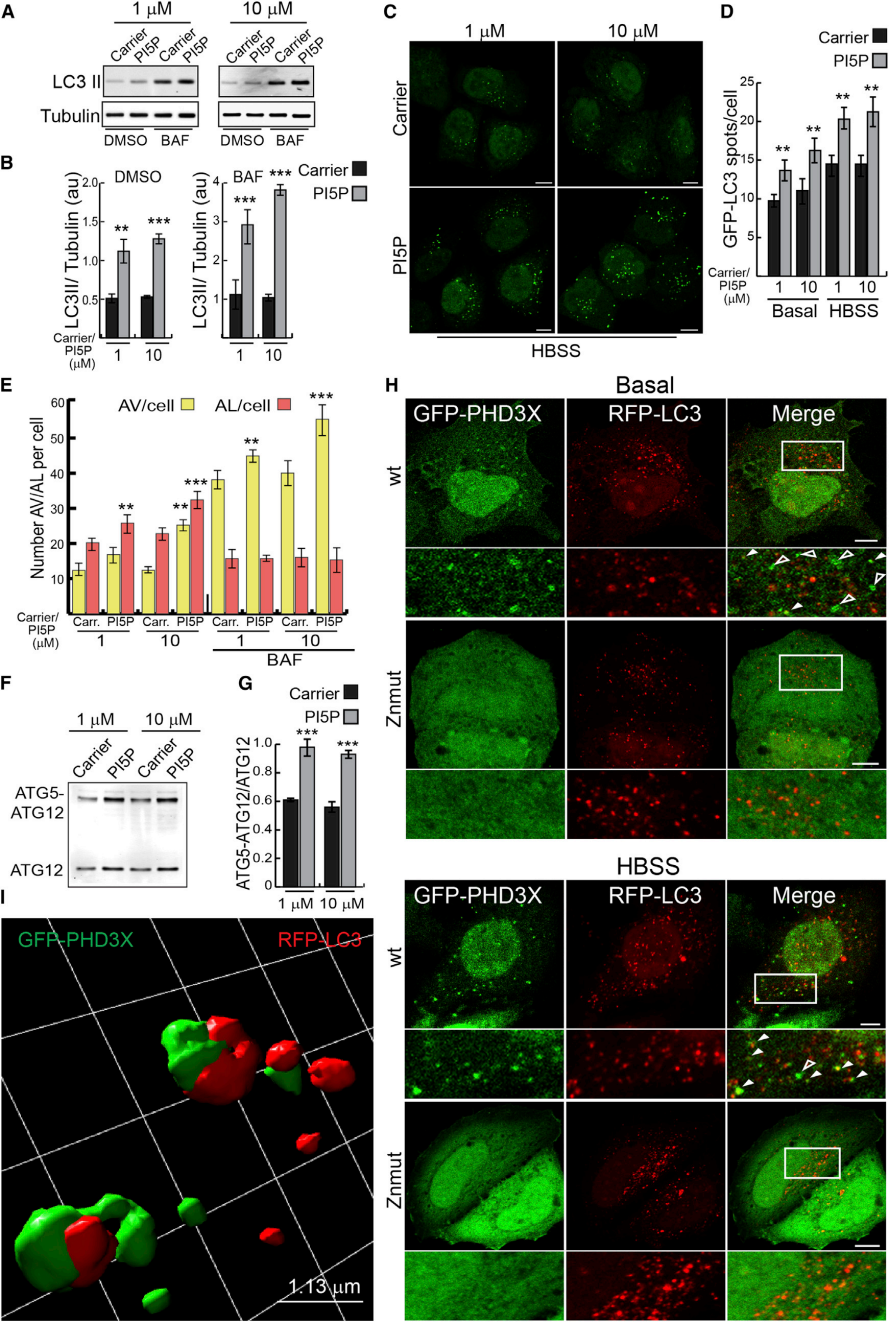
PI(5)P Regulates Autophagosome Biogenesis (A and B) Western blot analysis of LC3-II and tubulin levels and quantification of LC3-II/tubulin ratio in HeLa cells treated with carrier alone, or in combination with PI(5)P di-C16 at indicated concentrations for 1 hr, in the absence and presence of 400 nM BAF (treated in combination with lipids). Note that LC3-I is often very faint compared with LC3-II in HeLa cells under the protein extraction conditions we used (see Figure S1C). However, this was not a problem because one should relate LC3-II to tubulin (mean ± SEM). (C and D) HeLa cells stably expressing GFP-LC3 were treated as in (A) and then left in complete media (basal) or starvation media (HBSS) for 2 hr, then fixed and analyzed on a Cellomics ArrayScan system. Quantification of numbers of GFP-LC3 vesicles per cell in the different conditions is shown in (D) (mean ± SEM). (E) HeLa cells stably expressing GFP-mRFP-LC3 were treated as in (A) and analyzed on a Cellomics ArrayScan system. Quantification of numbers of autophagic vesicles (AV) or autolysosomes (AL) per cell in the different conditions is shown in the graph (mean ± SEM). (F and G) Western blot analysis of free ATG12 and ATG5-ATG12 complex levels with anti-HA antibody in HeLa cells transfected with HA-ATG12 and ATG5 and loaded with exogenous lipids. We quantified the ratio of ATG5-ATG12 versus free ATG12 by direct infrared fluorescence detection on an Odyssey Infrared Imaging System to assess ATG5-ATG12 conjugation efficiency (mean ± SEM). (H) HeLa cells transfected with GFP-PHD3X or GFP-PHD3X Znmut and RFP-LC3 for 16 hr were left in complete media (basal) or starvation media (HBSS) for 1 hr, then fixed and imaged on a confocal microscope. Empty arrowheads indicate PHD3X single-labeled structures, while filled arrowheads indicate PHD3X-LC3- and PHD3X-ATG16-positive structures. Bar, 10 μm. (I) HeLa cells transfected with GFP-PHD3X and RFP-LC3 for 16 hr were left in HBSS for 1 hr, then fixed and imaged on Elyra superresolution microscope. Final visualization was performed in Volocity 6.3 software using isosurface rendering of selected cropped regions of the data sets. Note that this rendering means that vesicles positive for green and red do not look yellow but have green and red on the surface. Representative cropped regions from different cells are shown. Bar, 1.13 μm. See also Figure S1.

Autophagosome numbers can be measured by assessing phosphatidylethanolamine-conjugated ATG8/LC3 (LC3-II) levels versus a loading control (e.g., tubulin), or by scoring the number of LC3-positive vesicles, since LC3-II is specifically targeted to autophagosomal membranes (Kabeya et al., 2000). In the same way that PI(3)P alone is sufficient to stimulate autophagy in mammalian cells (Petiot et al., 2000), we found that addition of exogenous PI(5)P di-C16 significantly increased LC3-II levels and LC3-positive autophagic vesicle numbers (Figures 1A–1D). While LC3-II levels correlate with autophagosome numbers, these can increase due to enhanced formation or decreased degradation (Rubinsztein et al., 2009). Consistent with a role in autophagosome synthesis, PI(5)P increased LC3-II levels, in an apparently dose-dependent manner, when we blocked LC3-II clearance by inhibiting autophagosome-lysosome fusion using Bafilomycin A1 (BAF) (Figures 1A and 1B and Figures S1A and S1B available online), and resulted in increased numbers of GFP-LC3 puncta (autophagosomes) in both nutrient-replete media and Hank’s balanced salt solution (HBSS) (amino-acid/serum starvation and 1 g/l D-glucose, compared to 4.5 g/l in Dulbecco’s modified Eagle’s medium [DMEM]) (Figures 1C and 1D). The effects of PI(3)P (a positive control) were stronger at 0.1 μM compared to PI(5)P, while the effects of PI(5)P were more noticeable at 1 and 10 μM (Figures S1A and S1B). PI and PI(4)P did not obviously stimulate autophagy, and PI(4)P loading of cells treated with BAF suggested possible inhibition of autophagy (Figure S1C). PI(5)P loading in cells stably expressing mRFP-GFP tandem fluorescent-tagged LC3 (see Supplemental Experimental Procedures) increased the numbers of autophagosomes and autolysosomes (Figures 1E and S1D). Furthermore, addition of PI(5)P increased the conjugation of ATG5 with ATG12 (Figures 1F and 1G), a critical event in phagophore biogenesis that appears to be regulated by PI(3)P (Ravikumar et al., 2008).

Exogenous PI(5)P was delivered to autophagic structures, as we observed fluorescent BODIPY-labeled PI(5)P on RFP-LC3 puncta (Figure S1E). BODIPY-labeled PI(5)P loading of living cells increased the number of RFP-LC3 dots dose dependently (Figure S1E). One difficulty in studying the intracellular localization of endogenous PI(5)P is the uncertain specificity of PI(5)P bioprobes. While the plant homeodomain (PHD) of ING2 (Bua et al., 2013; Gozani et al., 2003) and the pleckstrin homology (PH) domain of Dok proteins (Guittard et al., 2009, 2010) show strong preference for PI(5)P and have been used for intracellular localization and manipulation of PI(5)P (Viaud et al., 2014a), we cannot exclude that they do not bind other lipids to some extent. With this caveat in mind, we detected GFP-tagged PHD3X (three tandem repeats of PHD of ING2) (Bua et al., 2013; Gozani et al., 2003; Guittard et al., 2010) localization at the nucleus and plasma membrane (as previously described) and on discrete puncta, 25% of which were RFP-LC3 positive (Figure 1H). When cells were starved, GFP-PHD3X-positive structures increased from 4–6 to >12 spots per cell, 50% of which colocalized with RFP-LC3 (Figure 1H). A PHD mutant defective in PI(5)P binding (PHD3X Znmut) (Bua et al., 2013) failed to localize on discrete structures (Figure 1H). Superresolution structured illumination microscopy (SR-SIM) confirmed the confocal localization of PHD3X on LC3-positive vesicles/autophagosomes (Figure 1I; Movie S1).

Overexpression of GFP-PHD3X, which sequesters intracellular PI(5)P, dramatically decreased the percentage of cells with more than 10 LC3 dots (both endogenous LC3 and RFP-tagged LC3 were assessed), compared to GFP-empty vector or GFP-PHD3X Znmut (Figures S1F–S1I), an effect mimicked by overexpression of GFP-PH-Dok-5, an alternative PI(5)P binding module (Guittard et al., 2010; Figures S1F and S1G). As we could not exclude that some of the effects of these probes may be due to binding to other lipids, we next manipulated intracellular PI(5)P levels by targeting enzymes relevant for its biogenesis and turnover.

### PI(5)P Synthesized by PIKfyve Regulates Autophagosome Formation

**Figure 2:**
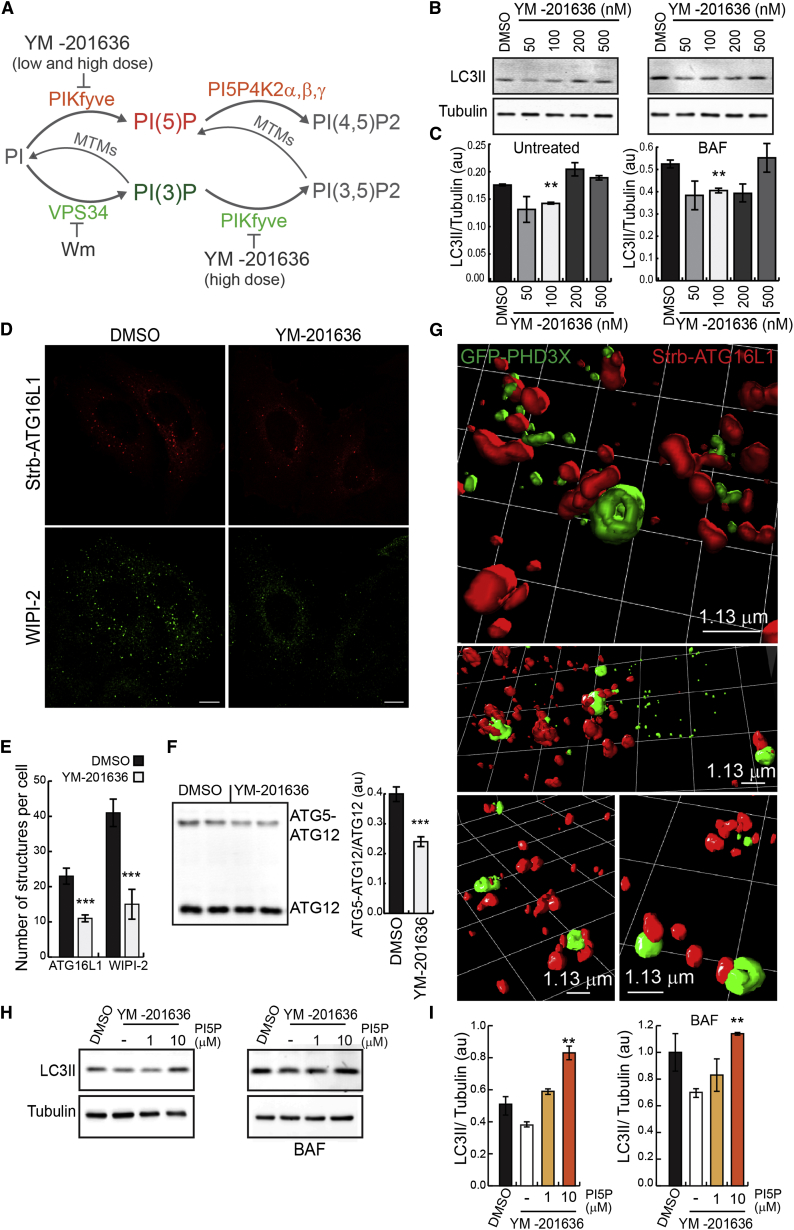
PI(5)P Synthesized by PIKfyve Regulates Autophagosome Formation (A) Schematic representation of pathways for PI(5)P and PI(3)P synthesis/turnover and drugs targeting enzymes involved in these pathways. (B and C) Western blot analysis of LC3-II and tubulin levels and quantification of LC3-II/tubulin ratio in HeLa cells treated with DMSO or increasing concentrations of YM-201636 for 2 hr in the presence or absence of BAF (mean ± SEM). (D and E) HeLa cells transiently transfected with Strawberry-ATG16L1 were treated with YM-201636 (100 nM, 2 hr) in HBSS, then fixed and stained for endogenous WIPI-2. Bar, 10 μm. (E) Quantification of ATG16L1 and WIPI2 structures per cell, n = 20 cells (mean ± SEM; n = 3 independent experiments; ^∗∗∗^p < 0.001, t test). (F) Western blot analysis of free ATG12 and ATG5-ATG12 complex levels with anti-HA antibody in HeLa cells transfected with HA-ATG12 and ATG5 and treated with YM-201636 (100 nM, 2 hr) (mean ± SEM). (G) HeLa cells transfected with GFP-PHD3X and Strawberry-ATG16L1 for 16 hr were left in HBSS for 1 hr, then fixed and imaged on Elyra superresolution microscope. Final visualization was performed as previously described. Bar, 1.13 μm. (H and I) Western blot analysis of LC3-II and tubulin levels and quantification of LC3-II/tubulin ratio in cells incubated with YM-201636 (100 nM, 2 hr) and loaded with exogenous PI(5)P for the last 1 hr in the presence or absence of BAF (mean ± SEM). See also Figure S2.

Type III PtdInsP 5-kinase PIKfyve appears to regulate PI(5)P biosynthesis (Figure 2A; Sbrissa et al., 1999), as reduced PI(5)P levels are seen in *PIKfyve* hypomorph and heterozygous mice and in cells silenced by small interfering RNA (siRNA), overexpressing a dominant-negative mutant or treated with pharmacological inhibitor of the kinase (Ikonomov et al., 2011; Sbrissa et al., 2002, 2012; Zolov et al., 2012). Low doses (100 nM) of the PIKfyve inhibitor YM-201636 (Sbrissa et al., 2012) decreased LC3-II levels (Figures 2B, 2C, and S2A), the number of ATG16L1 (autophagosome precursor) and WIPI-2 vesicles (Figures 2D and 2E), and ATG5-ATG12 conjugation (Figure 2F).

Autophagosomes may contain PI(5)P from early stages of their biogenesis, since GFP-PHD3X was associated with mStrawberry-ATG16L1 structures (which label phagophores or prephagophore structures) in both nutrient-replete and starved conditions (50% and 70% colocalization, respectively) (Figure S2B). A 3D analysis of PHD3X-labeled structures assessed by superresolution structured illumination microscopy (SR-SIM) revealed PI(5)P on ATG16L1-positive vesicles in autophagy-stimulating conditions (amino-acid starvation, HBSS media) (Figure 2G; Movie S2). Low doses of YM-201636 (100 nM) or PIKfyve silencing selectively depleted GFP-PHD3X on ATG16L1 vesicles, consistent with a specific reduction of PI(5)P on autophagosome precursors (Figures S2B and S2C). To further validate the idea that PI(5)P is present on early autophagic membranes, we expressed the GFP-PHD3X probe, in cells where phagophores accumulated and autophagosome completion was impaired by the overexpression of proteolytic-activity-deficient mutant of ATG4B (ATG4BC74A), that prevents LC3 lipidation and inhibits autophagosome formation (Fujita et al., 2008). ATG4BC74A increased the numbers of ATG16L1 structures labeled with GFP-PHD3X, in both basal and starvation conditions (Figure S2D), with some forming large ring-shaped structures.

PIKfyve is also the primary kinase responsible for production of PI(3,5)P2, a key regulator of early-to-late endosome membrane trafficking (Shisheva, 2013; Figure 2A). Note that higher concentrations and longer treatment with YM-201636, which inhibits PI(3)P conversion to PI(3,5)P2, causing elevation of PI(3)P (de Lartigue et al., 2009; Zolov et al., 2012), do not reduce autophagosome numbers (Figures 2B, 2C, and S2A), possibly due to defects in the endosomal/lysosomal compartment that block autophagosome degradation (Figures 2A, S2A, and S2E). Consistent with this, a similar block in autophagosome degradation is seen with PIKfyve siRNA knockdown (Figure S2F). To parse out the effects of impaired autophagosome formation and degradation, we treated cells with BAF for 2 hr in the presence of HBSS (an autophagy stimulus), and we found less autophagosome formation in the PIKfyve knockdown cells (Figure S2G), consistent with reduced ATG5-ATG12 conjugation after PIKfyve knockdown (as seen with short-term low-dose YM-201636 treatment) (Figure S2H). Importantly, the addition of exogenous PI(5)P di-C16 to cells treated with low concentrations of YM-201636 (Figures 2H and 2I) or treated with PIKfyve siRNA (Figures S2G and S2H) significantly rescued LC3-II levels and ATG5-ATG12 conjugation, arguing that PI(5)P is the relevant signaling molecule in PIKfyve-dependent autophagosome biogenesis.

Several in vivo studies suggest that PIKfyve may indirectly control PI(5)P levels by producing PI(3,5)P2, which is then transformed into PI(5)P by 3-phosphatases of the myotubularin family (Figure 2A; Oppelt et al., 2013, 2014; Viaud et al., 2014b; Zolov et al., 2012). If this is the case, then the production of PI(5)P is likely localized to internal membranes where PI(3,5)P2 is located. We investigated the localization of PI(3,5)P2 using the tandem repeats of the N-terminal domain of mucolipin1 (ML1N^∗^2), recently described as a reliable and specific probe for PI(3,5)P2 (Li et al., 2013). GFP-ML1N^∗^2 associated with early autophagosomal structures labeled with mStrawberry-ATG16L1 under nutrient-starvation conditions (80% of ATG16 structures contain GFP-ML1N^∗^2 during starvation versus 20% in complete media), and this association was sensitive to YM-201636 treatment (Figure S2I). Consistent with PI(3,5)P2 being a precursor of PI(5)P, we found that overexpression of myotubularin-related phosphatase 3 (MTMR3), one of the myotubularin PI 3-phosphatase enzymes previously implicated in PI(5)P production along with PIKfyve (Oppelt et al., 2013, 2014; Viaud et al., 2014b; Zolov et al., 2012), increased numbers of GFP-PHD3X structures, while its knockdown had the reverse effect (Figure S2J) during nutrient starvation (HBSS). Various myotubularin family members have been linked to autophagy, mainly in the regulation of PI3P metabolism (Taguchi-Atarashi et al., 2010; Vergne et al., 2009; Zou et al., 2012). Thus, myotubularins other than MTMR3 may also regulate PI(5)P levels during autophagy induction, like PI(3)P. PI(3,5)P2 localization during starvation resembles what we found for PI(5)P, and raises the possibility that PI(3,5)P2 may contribute as a precursor for the PI(5)P pool required for autophagy (Figure 2A). PI(5)P and its precursor PI(3,5)P2 localize on autophagosome precursors, and inhibition of their synthesis results in autophagy inhibition. Our data provide clear evidence that PI(5)P synthesis occurs during early stages of autophagosome formation and is modulated by nutrient state.

### Kinases that Convert PI(5)P to PI(4,5)P2 Regulate Autophagy

**Figure 3:**
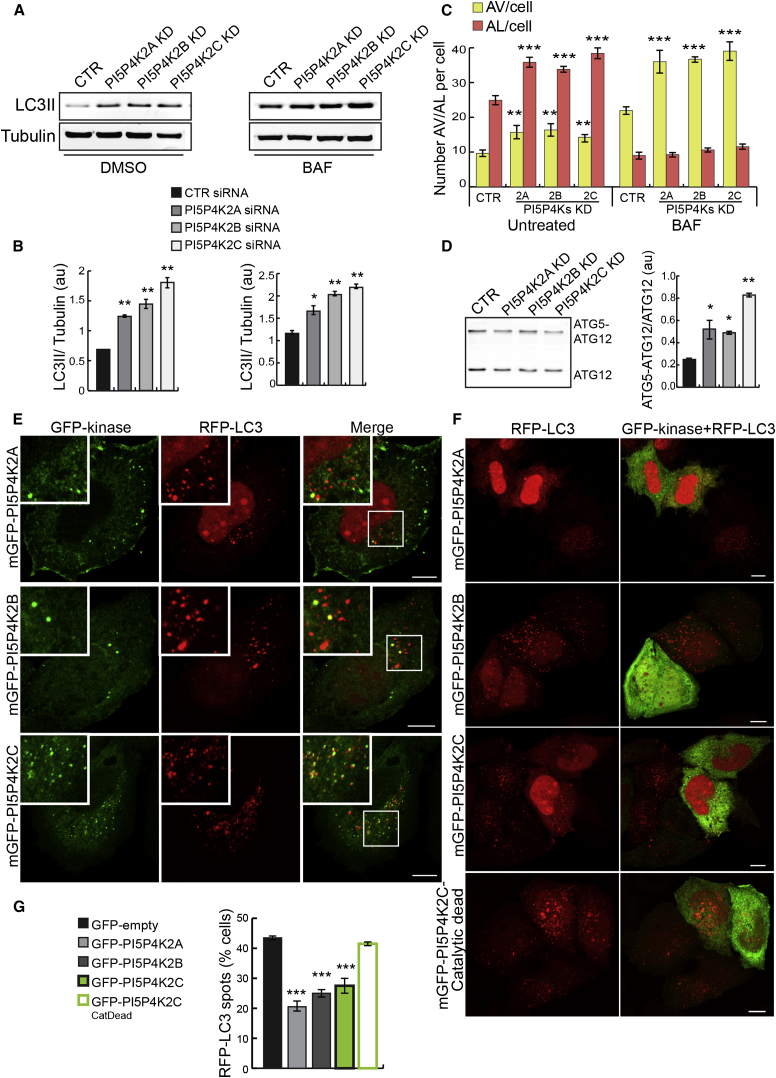
PI5P4K2s Localize on Autophagosomes and Regulate Autophagy (A and B) Western blot analysis of LC3-II and tubulin levels and quantification of LC3-II/tubulin ratio in HeLa cells transfected for 5 days with two rounds of control, PI5P4K2A, 2B, or 2C siRNA either left untreated or treated with BAF (200 nM, 16 hr) (mean ± SEM). (C) HeLa cells stably expressing GFP-mRFP-LC3 treated as in (A) were analyzed on a Cellomics ArrayScan system as previously described (mean ± SEM). (D) Western blot analysis of free ATG12 and ATG5-ATG12 complex levels with anti-HA antibody in HeLa cells treated with control, PI5P4K2A, 2B, and 2C siRNA and transfected with HA-ATG12 and ATG5 for the last 16 hr (mean ± SEM). (E and F) HeLa cells transfected with GFP-PI5P4K2A, 2B, 2C, or catalytic-dead PI5P4K2C along with RFP-LC3 for 16 hr (E) or 30 hr (F) were fixed and imaged on a confocal microscope. Bar, 10 μm. (G) Quantification of cells (percentage of total) showing more than ten autophagic vesicles (RFP-LC3 vesicles) in the different conditions from (F) is shown in the graph; n = 200 cells (mean ± SEM). See also Figure S3.

The major route for PI(5)P removal is attributed to type II PI5PK kinases (phosphatidylinositol 5-phosphate 4-kinases, PI5P4K2s) (Figure 2A; Clarke et al., 2010; Shisheva, 2013; Viaud et al., 2014a). Mammalian genomes contain three genes, PI5P4K2A, B, and C, encoding three type II PI(5)P 4-kinase isoforms, alpha (PI5P4K2α), beta (PI5P4K2β), and gamma (PI5P4K2γ), respectively. Silencing of the three PI5P4K2s, which increase cellular PI(5)P levels (Sarkes and Rameh, 2010; Wilcox and Hinchliffe, 2008), increased autophagosome formation (increased levels of LC3-II in the presence of BAF) (Figures 3A, 3B, and S3A–S3E), autophagosome and autolysosome numbers (in cells stably expressing mRFP-GFP tandem fluorescent-tagged LC3) (Figures 3C and S3F), and ATG5-ATG12 conjugation (Figure 3D). PI5P4K2A, B, and C silencing resulted in more obvious localization of GFP-PHD3X on autophagosome precursors and mature autophagosomes (ATG16L1 and RFP-LC3-positive structures) (Figure S3G). Consistent with the distribution of PI(5)P (Figure 1H), PI5P4K2α, β, and γ localized to different extents on autophagosomes (RFP-LC3 structures, Figure 3E), with PI5P4K2γ showing the most prominent association with autophagosomal structures. The different PI5P4K2 isoforms appeared to have different efficacies. While this may be due to different knockdown efficiencies, we noted that PI5P4K2C, whose knockdown had the strongest effect, was also most obviously associated with autophagosomes. Overexpression of active GFP-tagged PI5P4K2α, β, and γ, which attenuate signaling from PI(5)P (by consuming PI(5)P to produce PI(4,5)P2), impaired autophagy (Figures 3F and 3G), while a GFP-tagged PI5P4K2γ-inactive mutant did not (Figures 3F and 3G). These data indicate a function for PI(5)P in the induction of autophagy.

### PI5P4K2s Alter the Levels of Autophagy Substrates

**Figure 4:**
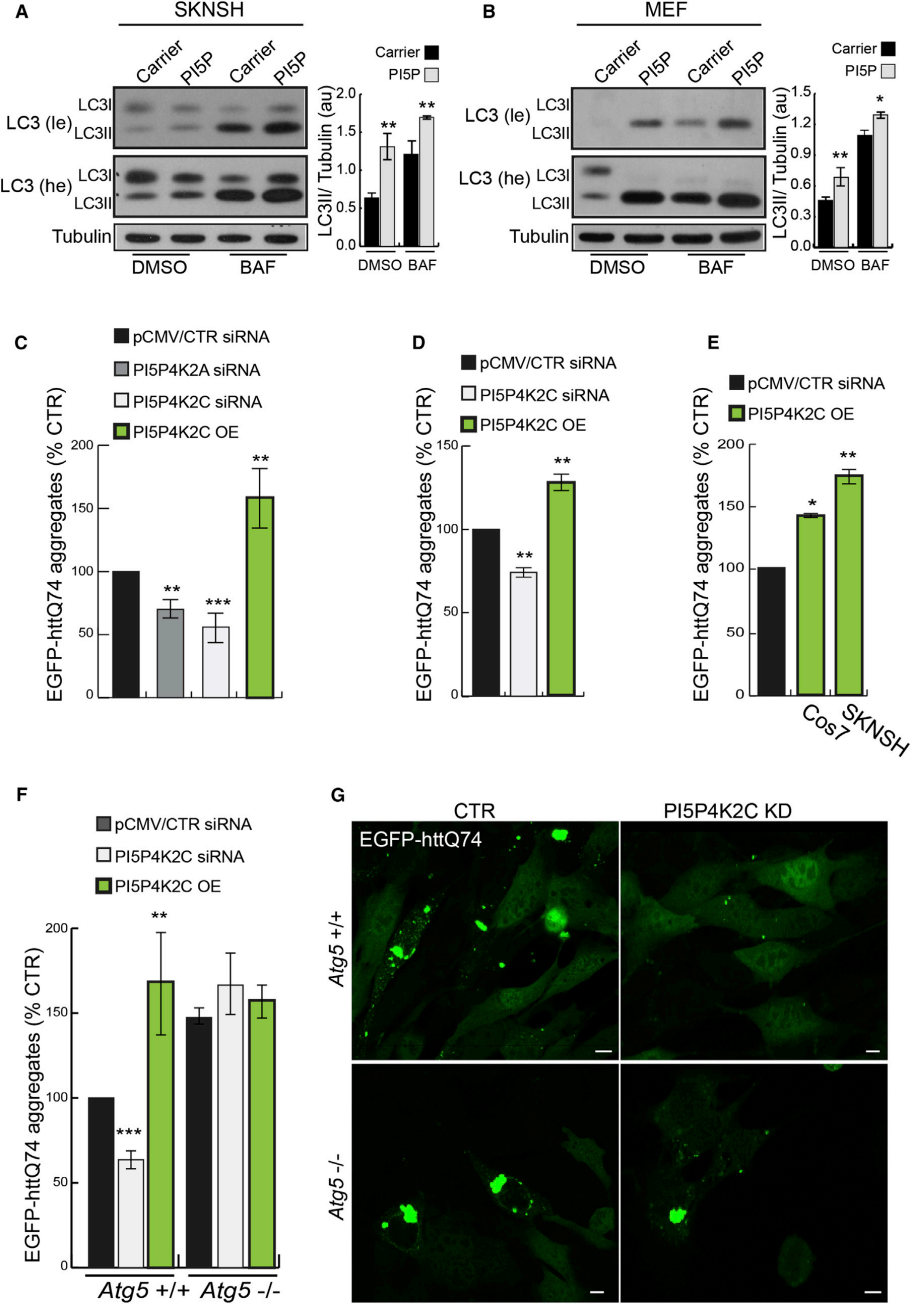
PI5P4K2A and PI5P4K2C Regulate Accumulation of Mutant Huntingtin Exon 1 (A and B) Western blot analysis of LC3-II and tubulin levels and quantification of LC3-II/tubulin ratio in SKNSH and MEFs treated for 2 hr with carrier alone or in combination with PI(5)P di-C16 (10 μM) in the absence and presence of BAF (mean ± SEM). (C) HeLa cells treated with PI5P4K2A or PI5P4K2C siRNA for 5 days or with pCMV-PI5P4K2C overexpression construct for 48 hr were transfected with EGFP-httQ74 for the last 48 hr. Percentage of cells with EGFP-positive aggregates is shown in the graph; n = 500 cells (mean ± SEM). Cells expressing control vector typically have 25% of aggregates and we set the control at 100% to enable comparison and statistics from independent experiments. (D and E) Percentage of cells with EGFP-positive aggregates was scored in HEK293 cells treated with PI5P4K2C siRNA for 5 days and cotransfected with EGFP-httQ74 for the last 48 hr (D), or in COS7 and SKNSH cells (E), transfected with PI5P4K2C plasmid together with EGFP-httQ74 for the last 48 hr. (F and G) WT (*Atg5+/+*) or autophagy-deficient cells lacking the key autophagy gene *Atg5* (*Atg5−/−*) MEFs were treated with PI5P4K2C siRNA (5 days) or PI5P4K2C plasmid (48 hr) together with EGFP-httQ74 for 48 hr. The percentages of transfected cells with EGFP-httQ74 aggregates were assessed and shown in the graph; n = 500 cells (mean ± SEM). Representative confocal images are shown in (G). See also Figures S3 and S4.

PI(5)P loading significantly increased LC3-II levels (in the absence and presence of BAF) in both human neuroblastoma (SKNSH) and mouse embryonic fibroblasts (MEFs) (Figures 4A and 4B). We investigated the effects of PI5P4K2s knockdown and overexpression on the levels of diverse autophagic substrates, including the ubiquitin-binding adaptor protein p62 and a mutant form of huntingtin associated with Huntington’s disease (EGFP-httQ74) (Ravikumar et al., 2002). PI5P4K2s knockdown decreased the levels of p62 (Figures S3A–S3C) and the numbers of cells with mutant huntingtin aggregates (Figures 4C and 4D), which correlate with mutant protein levels (Narain et al., 1999), like other autophagy inducers (Williams et al., 2008), while PI5P4K2C overexpression had the opposite effect (Figures 4C–4E). While PI5P4K2C silencing decreased the proportion of cells with EGFP-httQ74 aggregates and the opposite was seen with PI5P4K2C overexpression, these effects were only seen in wild-type (WT, *Atg5*^*+/+*^) MEFs, and not in autophagy-deficient cells (*Atg5*^*−/−*^*)*, suggesting these PI5P4K2C effects are autophagy dependent (Figures 4F, 4G, S4A, and S4B).

### PI(5)P Sustains Autophagy in Cells Depleted of PI(3)P

**Figure 5:**
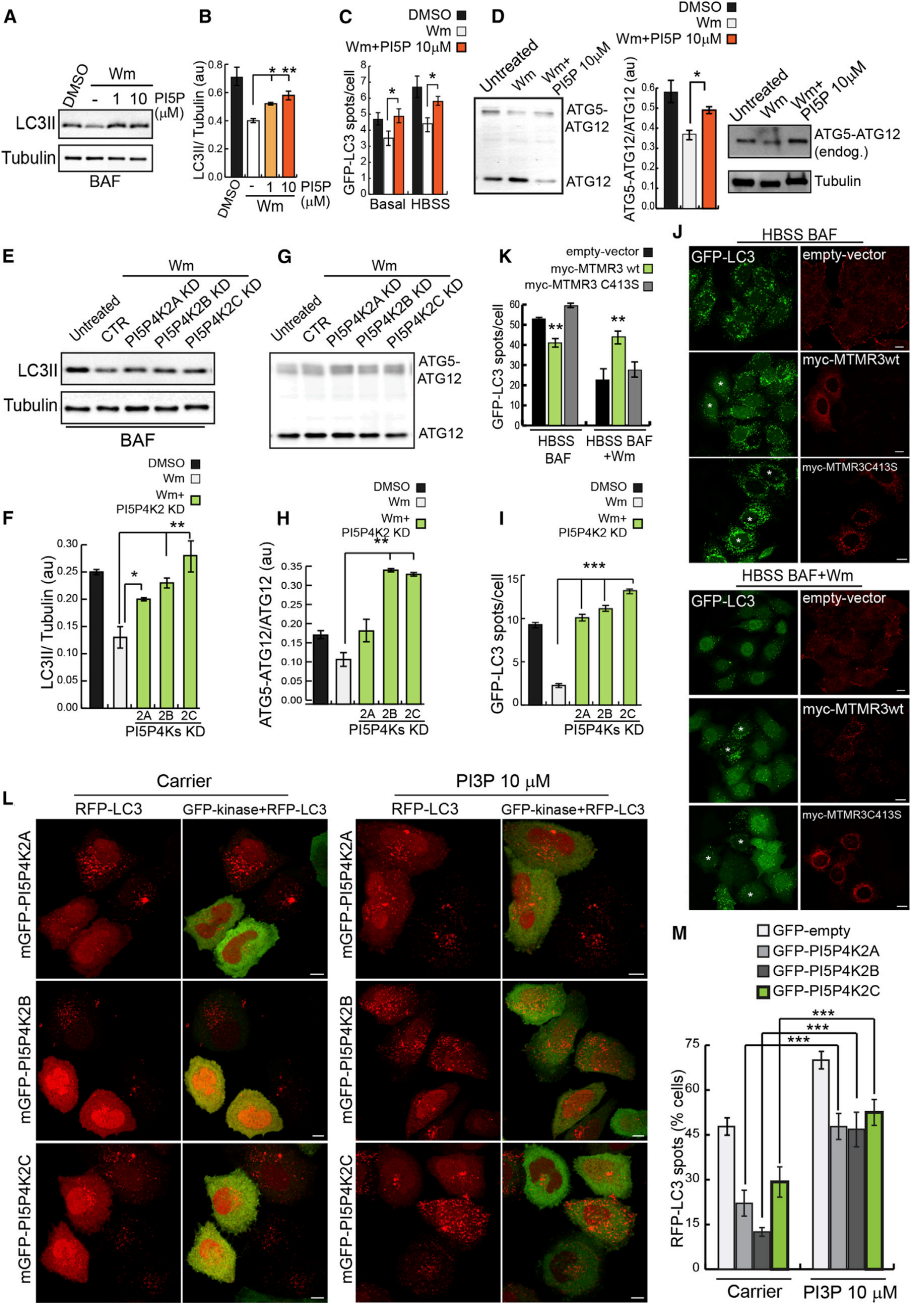
PI(5)P Triggers Autophagy in the Absence of VPS34 Activation (A and B) Western blot analysis of LC3-II and tubulin levels and quantification of LC3-II/tubulin ratio in HeLa cells pretreated with Wm (200 nM, 2 hr), then loaded with 10 μM PI(5)P for 1 hr (in the presence of Wm and BAF) (mean ± SEM). (C) Quantification of numbers of GFP-LC3 vesicles in HeLa cells stably expressing GFP-LC3 treated as in (A) and then shifted to starvation media (HBSS) or complete media (Basal) for 2 hr (in the presence of Wm) (mean ± SEM). (D) HeLa cells transfected with HA-ATG12 and ATG5 treated with 200 nM Wm as in (A) and subjected to western blot analysis with anti-HA antibody to detect free ATG12 and the ATG5-ATG12 complex (mean ± SEM). ATG5-ATG12 conjugation was checked for endogenous proteins in the same conditions using an anti-ATG12 antibody (bottom). (E and F) Western blot analysis of LC3-II and tubulin levels and quantification of LC3-II/tubulin ratio in HeLa cells transfected with control, PI5P4K2A, 2B, and 2C siRNA and treated with Wm (200 nM, 2 hr in the presence of BAF) (mean ± SEM). (G and H) Western blot analysis of free ATG12 and ATG5-ATG12 complex levels with anti-HA antibody in HeLa cells treated with control, PI5P4K2A, 2B, and 2C siRNA, transfected with HA-ATG12 and ATG5 for last 16 hr and treated with Wm for 2 hr (mean ± SEM). (I) HeLa cells stably expressing GFP-LC3 treated with control, PI5P4K2A, 2B, and 2C siRNA, were pretreated with Wm for 2 hr in complete medium and then shifted in HBSS media for 2 hr (in the presence of Wm) (mean ± SEM). (J and K) HeLa cells stably expressing GFP-LC3 were transfected for 30 hr with myc-tagged empty vector, myc-MTMR3WT, and myc-MTMR3C413S, incubated for 4 hr in HBSS in the presence of BAF and in the presence or absence of Wm. Cells were fixed, stained with anti-myc antibodies, and imaged by confocal microscope. Asterisks indicate transfected cells. Quantification of numbers of GFP-LC3 vesicles per cell is shown in (K) (mean ± SEM). (L and M) HeLa cells transfected with GFP-PI5P4K2A, 2B, 2C, and RFP-LC3 for 30 hr were loaded with 10 μM PI(3)P for 1 hr in complete medium, and then fixed and imaged on confocal microscope. Bar, 10 μm. Quantification of cells (percentage of total) showing more than 10 RFP-LC3 vesicles in the different conditions from (J) is shown in (M); n = 200 cells (mean ± SEM). See also Figure S5.

We tested whether PI(5)P could sustain autophagy in cells depleted of PI(3)P with the VPS34 inhibitor Wm. We validated the specificity of Wm for VPS34 activity during autophagy induction by treating VPS34-null MEFs (Figure S5A) and HeLa or GFP-LC3 HeLa stable cell lines with Wm for 2 hr in HBSS (Figures S5B and S5C). Consistent with previous studies, Wm inhibited ATG5-ATG12 conjugation, LC3 lipidation, and prevented autophagosome formation (Blommaart et al., 1997; Ravikumar et al., 2008; Figures S5A–S5D; Figures 5A–5D). VPS34-null MEFS are insensitive to Wm compared to their WT counterparts (Figure S5A), and autophagy inhibition caused by Wm in HBSS-treated cells was rescued by PI(3)P supplementation (Figures S5B and S5C). VPS34-null cells did not have obviously decreased autophagy in HBSS (Figure S5A) like Wm-treated cells, suggesting that long-term VPS34 depletion may be compensated for by alternative pathways or by additional defects in autophagosome degradation (Devereaux et al., 2013). PI(3)P also regulates proteins crucial for sorting to vacuoles or lysosomes (Schink et al., 2013), and long-term VPS34 inhibition can impair endocytic trafficking (Petiot et al., 2003; Siddhanta et al., 1998) and cause vacuolation of late endosomal compartments (Futter et al., 2001) and mistrafficking of cathepsin D from late endosomes to lysosomes (Row et al., 2001).

To test whether PI(5)P was able to sustain autophagy in cells depleted of PI(3)P, we used acute Wm treatment and loaded cells with exogenous PI(5)P di-C16. Under these conditions, the requirement for the generation of PI(3)P appeared to be abrogated, as we detected LC3 lipidation (Figures 5A and 5B), increased LC3 vesicles under starvation (Figures 5C and S5C), increased double-membraned autophagosomes by electron microscopy (Figure S5D), and ATG5-ATG12 conjugation (Figure 5D), even in the presence of Wm. Similarly, increasing endogenous PI(5)P levels by PI5P4K2s knockdown resulted in an increase in LC3-II levels (Figures 5E and 5F), ATG5-ATG12 conjugation (Figures 5G and 5H), and numbers of LC3 vesicles (Figures 5I and S5E) that were resistant to Wm.

As a genetic alternative to Wm, we performed a short-term siRNA knockdown of VPS34, which does dramatically impact autophagy in HBSS (in contrast to longer-term knockdowns or Cre-mediated gene excision in the MEFs) (Figure S5A). In such VPS34-siRNA-treated cells, PI(5)P was able to rescue autophagy and ATG5-ATG12 conjugation. (Figure S5F).

We also manipulated PI(5)P and PI(3)P levels by overexpressing MTMR3, which would decrease PI(3)P levels but increase PI(5)P levels (Figures 2A and S5G). MTMR3 overexpression (but not MTMR3C413S catalytic mutant overexpression) in GFP-LC3 HeLa cells incubated in HBSS impaired autophagosome formation (BAF for 4 h), as previously reported (Taguchi-Atarashi et al., 2010; Figures 5J and 5K). This effect is likely due to the effects of MTMR3 on PI(3)P catabolism, which would be expected to blunt the HBSS-induced autophagy-stimulating levels of this lipid. To measure the effect of MTMR3 overexpression in a context where the role of PI(3)P can be excluded (Figure 2A), to allow us to largely focus our attention on the ability of this enzyme to generate PI(5)P, we treated cells with BAF and Wm for 4 hr in the presence of HBSS, and we found that overexpression of WT, but not of catalytic dead MTMR3 (Figures 5J and 5K), could rescue LC3 vesicle numbers in Wm-treated cells. Conversely, increasing PI(3)P (by loading of exogenous PI(3)P di-C16) restored LC3 dots in cells depleted of PI(5)P by overexpression of active PI5P4K2α, β, and γ (Figures 5L and 5M). Thus, our results point toward a common mechanism of action for PI(3)P and PI(5)P during autophagy.

### PI(5)P and PI(3)P Share Common Effectors during Autophagosome Formation

**Figure 6:**
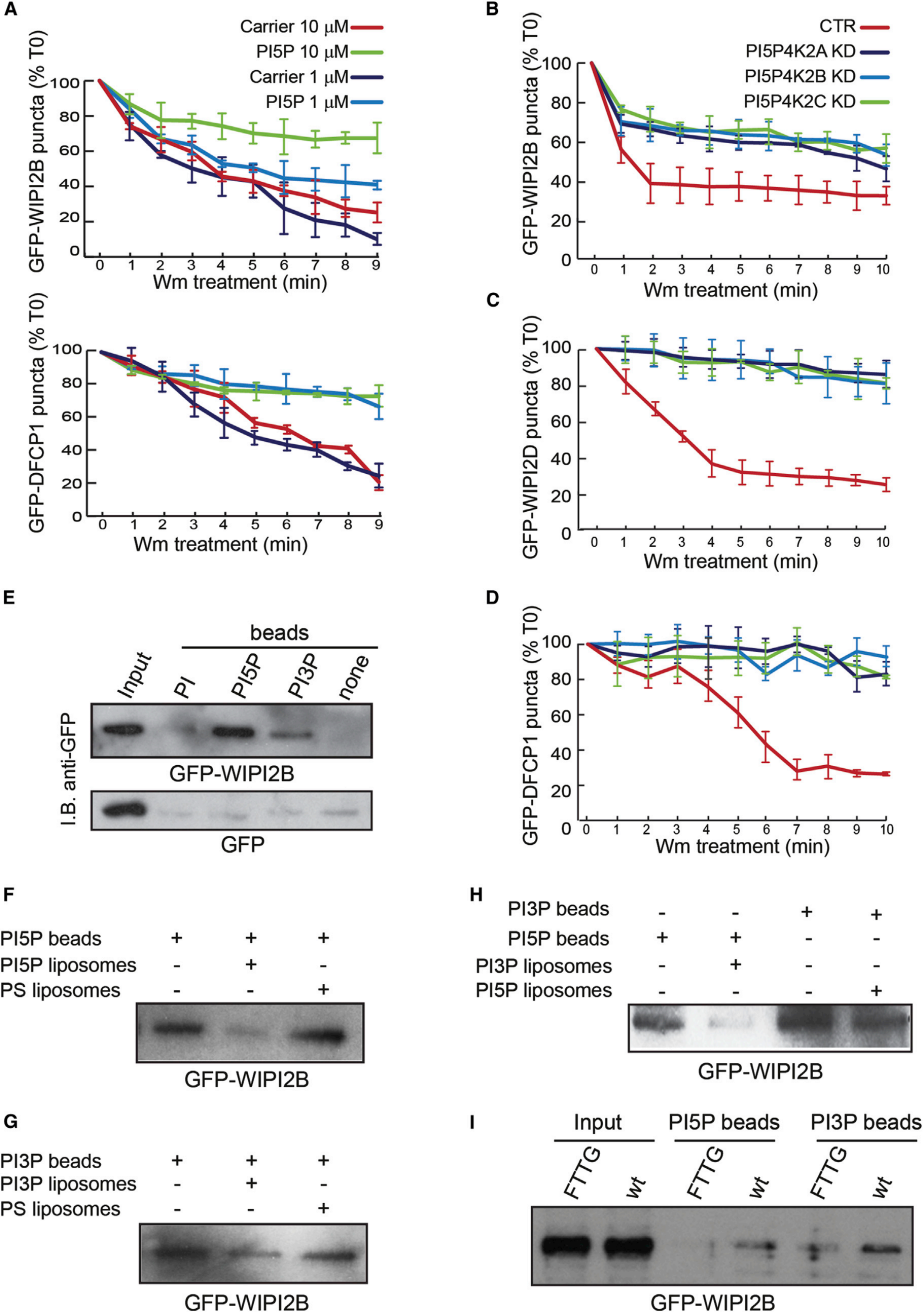
PI(5)P Recruits Proteins Required for the Initiation of Autophagosome Formation (A) HeLa cells transfected with GFP-WIPI2B or GFP-DFCP1 preloaded with indicated concentrations of PI(5)P for 1 hr, starved in HBSS for 1 hr, and then incubated with Wm in HBSS were tracked by time-lapse microscopy for 10 min after the addition of Wm. Quantification of WIPI2B or DFCP1 vesicles (percentage of those at the starting time [T0]) during the treatments are shown in the graphs. (B–D) HeLa cells treated with control, PI5P4K2A, 2B, and 2C siRNA were transfected with GFP-WIPI2B (B), GFP-WIPI2D (C), or GFP-DFCP1 (D); starved in HBSS (1 hr); and then incubated with Wm in HBSS. WIPI2 or DFCP1 structures were tracked and quantified as in (A). (E) Lysates from HeLa cells stably expressing GFP-WIPI2B were incubated with agarose beads coated with PI, PI(5)P, and PI(3)P, eluted with SDS-PAGE sample buffer, and recovered proteins were assessed by western blotting using antibodies against GFP. Uncoated beads and lysates from HeLa cells stably expressing GFP alone were used as internal controls. (F–H) Cell extracts from HeLa cells stably expressing GFP-WIPI2B were incubated for 3 hr with PI(5)P-containing liposomes (F and H) or PI(3)P-containing liposomes (G and H) before a pull-down experiment using the indicated beads. PS-containing liposomes were used as internal controls for both competition assays in (F and G). (I) Lysates from HeLa cells stably expressing GFP-WIPI2B WT and GFP-WIPI2B FTTG mutant were incubated with agarose beads coated with PI(5)P and PI(3)P, eluted with SDS-PAGE sample buffer, and recovered proteins were assessed by western blotting using antibodies against GFP. See also Figure S6.

Since PI(5)P can rescue autophagy in PI(3)P-depleted cells, we tested if PI(5)P regulates autophagosome formation similarly to PI(3)P, through the recruitment of the WIPI2 and DFCP1 proteins. GFP-WIPI2 and DFCP1 puncta, which disappeared in starved cells treated with Wm, remained when such Wm-treated cells were preloaded with PI(5)P (Figures 6A and S6A), and the membrane association of GFP-WIPI2 and GFP-DFCP1 was dramatically impaired by Wm, but was Wm-insensitive in PI5P4K2 knockdown cells (Figures 6B–6D and S6B). Thus, WIPI2 and DFCP1 membrane binding can be preserved by PI(5)P in Wm-treated cells.

We examined whether WIPI2 proteins bind to PI(5)P using extracts from cells expressing GFP-tagged WIPI2B and WIPI2D, which we then precipitated with beads coated with different PIs (Figures 6E and S6C). WIPI2 proteins were associated with PI(5)P beads, but not with PI or uncoated beads (Figures 6E and S6C). This association seemed to be specific because the PI(5)P binding was inhibited when cell extracts were preincubated with PI(5)P-containing but not PS-containing liposomes (Figure 6F). Similar effects also were observed for the binding of WIPI2B to PI(3)P beads and for the competition of PI(3)P-containing liposomes with PI(3)P beads (Figures 6E and 6G). WIPI2B protein pull-down by the PI(3)P beads was strongly affected by preincubation with PI(5)P-containing liposomes (Figure 6H). Likewise, WIPI2B protein pull-down by the PI(5)P beads was strongly affected by preincubation with PI(3)P-containing liposomes (Figure 6H). Furthermore, a WIPI2 mutant that was predicted not to bind to PI(3)P (Dooley et al., 2014) had similarly reduced binding to PI(5)P (Figure 6I). Thus, PI(5)P binds a PI(3)P effector involved in autophagosome biogenesis; we tested if PI(5)P was required for PI(3)P-independent autophagy.

### Autophagy Activation following Glucose Starvation Is Dependent upon PI(5)P, but Not PI(3)P

**Figure 7:**
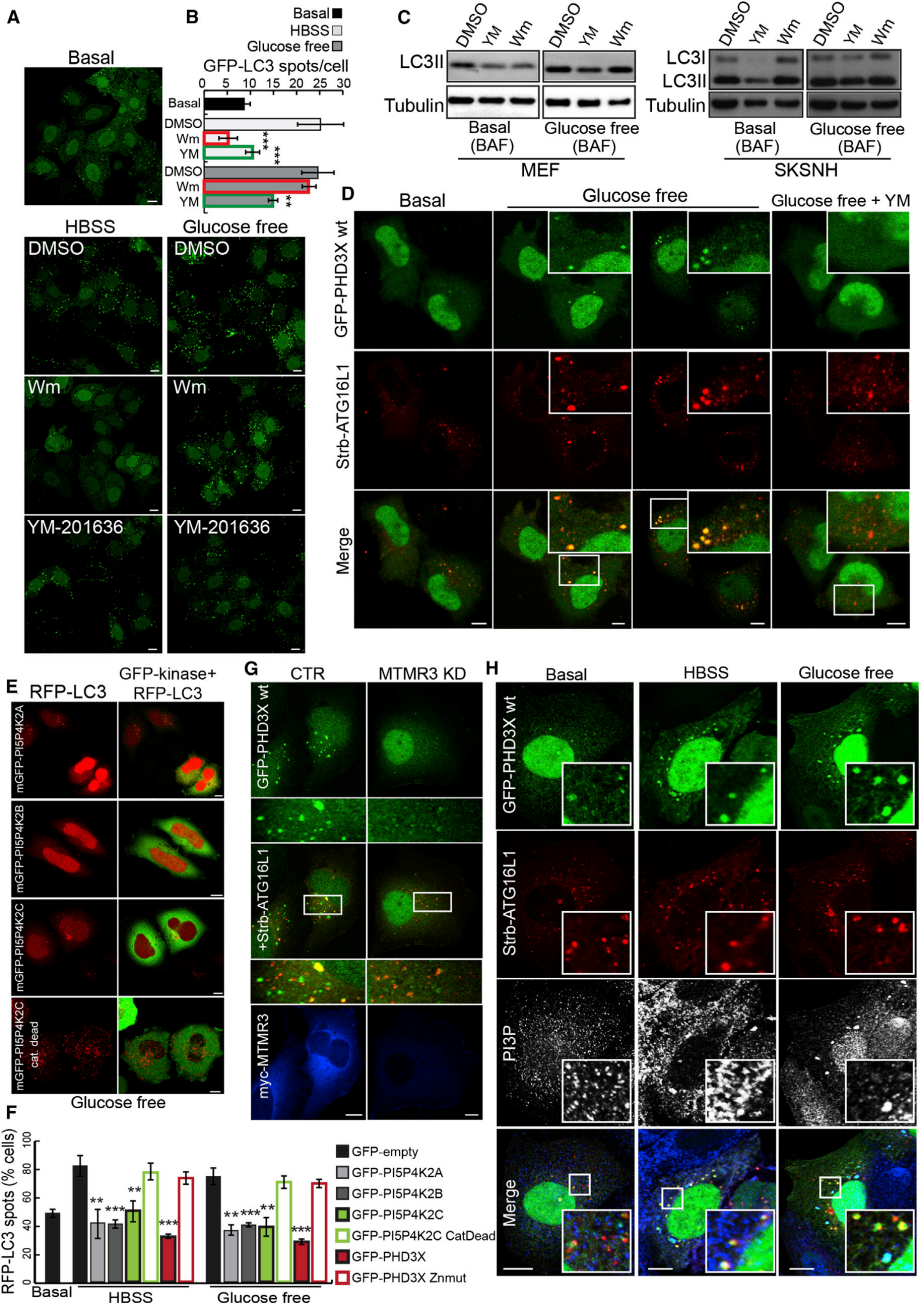
PI(5)P Is a Master Regulator of Autophagy in Response to Glucose Starvation (A) HeLa cells stably expressing GFP-LC3 were shifted to starvation media (HBSS or glucose-free DMEM) for 4 hr (in the presence of 200 nM Wm or 100 nM YM-201636). (B) Quantification of numbers of GFP-LC3 vesicles per cell in HeLa cells stably expressing GFP-LC3 treated as in (A) is shown in the graph (mean ± SEM). (C) Western blot analysis of LC3-II and tubulin levels in MEF and SKNSH cells were treated as in (A) in complete media or glucose-free media in the presence of 100 nM YM-201636, 200 nM Wm, and 400 nM BAF for 4 hr. (D) HeLa cells transfected with GFP-PHD3X and Strawberry-ATG16L1 for 16 hr were left in complete media (basal) or glucose-free media (glucose free) for 4 hr in the presence or absence of 100 nM YM-201636, then fixed and imaged on a confocal microscope. (E and F) HeLa cells transfected with GFP-PI5P4K2A, 2B, 2C, or PI5P4K2C catalytic dead and RFP-LC3 for 30 hr (E) were starved for glucose for 4 hr, then fixed and imaged on a confocal microscope. Bar, 10 μm. (F) Quantification of cells (percentage of total) showing more than ten autophagic vesicles (RFP-LC3 vesicles) in the different conditions from (F) is shown in the graph; n = 200 cells (mean ± SEM). (G) HeLa cells treated with CTR or MTMR3 siRNA for 5 days were transfected for the last 16 hr with GFP-PHD3X, Strb-ATG16L1, and myc-MTMR3 WT. Cells were starved for glucose for 4 hr, then fixed, stained with anti-myc antibodies, and imaged on a confocal microscope. Bar, 10 μm. (H) HeLa cells transfected for 16 hr with GFP-PHD3X and Strb-ATG16L1 were starved for 4 hr in HBSS or glucose-free media, then fixed, stained for PI(3)P antibodies, and imaged on a confocal microscope. Bar, 10 μm. See also Figure S7.

Since we found that PI(5)P can sustain autophagy in Wm-treated cells, we considered that PI(5)P may regulate autophagy pathways where PI(3)P is dispensable, such as glucose-starvation-induced autophagy (McAlpine et al., 2013), a hypothesis strengthened by our observation that HBSS or glucose starvation increase the phosphorylation of PIKfyve at residues where this enhances its catalytic activity (Er et al., 2013; Hill et al., 2010; Liu et al., 2013; Figure S7A). Glucose deprivation in HeLa cells stably expressing GFP-LC3 increased the number of autophagosomes in DMSO- and Wm-treated cells, but not when PIKfyve was inhibited by YM-201636 treatment (Figures 7A and 7B). Conversely, both Wm and YM-201636 strongly affected but did not completely ablate autophagosome formation under amino-acid/serum starvation (HBSS, Figures 7A and 7B). We confirmed that YM-201636 reduced LC3-II levels in both glucose-starved MEFs and SKNSH cells, and that this phenomenon was not seen with Wm (Figure 7C). Consistent with a specific requirement for PI(5)P for autophagy induction during glucose withdrawal, we observed a significant YM-201636-sensitive increase in the number of ATG16-positive autophagosomes containing PI(5)P in glucose-depleted cells (Figure 7D). When PI(5)P was sequestered by GFP-PHD3X overexpression or removed by overexpression of active PI5P4K2α, β, and γ, the appearance of LC3 dots following glucose starvation was abolished (Figures 7E, 7F, and S7B). Consistent with the different effects of Wm treatment under nutrient starvation, we did not detect an increased number of PI(3)P-containing autophagosomes (using the GFP-FYVE2X probe or anti-PI(3)P antibodies) in cells depleted of glucose compared to HBSS media (Figures S7C and S7D).

Supporting the idea that PI(3,5)P2 contributes to the generation of PI(5)P during nutrient starvation, we observed increased colocalization of GFP-ML1N^∗^2 with RFP-LC3 under glucose and amino-acid/serum starvation (Figure S7E). Indeed, MTMR3 knockdown significantly reduced PI(5)P on autophagosomes (Figure 7G), inhibited autophagosome formation in glucose-starved cells (where PI(3)P is dispensable), and this effect could be reversed by adding back PI(5)P (Figures S7F and S7G). Collectively, our results argue that PI(5)P generated by PIKfyve and MTMR3 is the relevant lipid species for autophagosome generation during glucose starvation. Resveratrol induces autophagy in noncanonical manner, independent of Beclin1 and partially resistant to Wm (Mauthe et al., 2011). The autophagy stimulation of resveratrol was impaired upon PI(5)P depletion due to overexpression of PI5P4K2α, β, and γ (but not the catalytic-dead mutant of PI5P4K2γ or GFP-empty vector) (Figure S7H).

When we labeled cells in basal, HBSS, or glucose starvation for PI(3)P (with an antibody) and for PI(5)P (with GFP-PHD3X), it was notable that the glucose-starved cells had fewer PI(3)P- and more PI(5)P-containing ATG16L1 structures, some of which appeared devoid of PI(3)P (Figure 7H), consistent with a dominant role for PI(5)P during glucose starvation. Our findings suggest that both PI(5)P and PI(3)P are important regulators of autophagy induced by HBSS. However, PI(5)P synthesis, but not PI(3)P synthesis, is required for autophagy induction by glucose withdrawal.

## Discussion

The present work identifies a role for PI(5)P as a regulator of autophagosome biogenesis. PI(5)P synthesis is required for autophagosome formation and this effect is similar to PI(3)P, as both lipids regulate recruitment of the WIPI2 and DFCP1 proteins and ATG5-ATG12 conjugation. PI(5)P and kinases acting on PI(5)P (particularly PI5P4K2γ) are associated with autophagosomes and autophagosome precursors, suggesting that local alterations in the levels of this lipid are important for regulating autophagy. While we cannot exclude that alterations in PI(5)P may impact autophagosome biogenesis through its metabolites, the observations that PI(5)P binds to WIPI2, impacts PI(3)P-related phenotypes like ATG5-ATG12 conjugation, and can rescue autophagy in Wm-treated HBSS-starved cells strongly argue that it can serve as an alternative to PI(3)P. It is worth bearing in mind that PI(5)P’s presence in cells is ∼100 times lower than those of the most abundant Pis, PI(4,5)P2 and PI(4)P (Sarkes and Rameh, 2010). The major source of PI(4,5)P2, which we previously have shown to be a positive regulator of autophagy (Moreau et al., 2012), is PI(4)P in resting cells (Rameh et al., 1997; Whiteford et al., 1997). Thus, reductions of PI(5)P levels hardly affect PI(4,5)P2.

The ability of PI(5)P to regulate autophagy in cells depleted of PI(3)P suggests that PI(5)P may account for the previously enigmatic concept of PI(3)P-independent, noncanonical autophagy. While we cannot exclude that a small amount of cellular PI(3)P may be generated via VPS34-independent routes, as suggested recently (Devereaux et al., 2013; McAlpine et al., 2013; Zhou et al., 2010), we consciously used acute depletion of PI(3)P using Wm to reduce the likelihood of such minor pathways having overt effects (compared to using genetic approaches, which would be necessarily more chronic and would allow redundant pathways to become more apparent). Indeed, Wm completely ablated the presence of WIPI2 and DFCP1 vesicles in our experiments. Since these effects and the concomitant ATG5-ATG12 conjugation and autophagosome formation were completely rescued by elevating PI(5)P in Wm-treated cells, we suggest that PI(5)P can account for the previously mysterious phenomenon of PI(3)P-independent autophagy. While this would be compatible with the similar binding of PI(3)P and PI(5)P to WIPI proteins (Baskaran et al., 2012; Jeffries et al., 2004), it is possible that the two lipids may have some nonredundant properties in the autophagy context. Indeed, this appeared to be the case, as we found PI(5)P (and its precursor PI(3,5)P2) localized on nascent and mature autophagosomes during glucose starvation, and this is highly dependent on the activity of the type III PtdInsP 5-kinase PIKfyve. Furthermore, glucose-starvation-induced autophagy appeared to be dependent on PI(5)P and not PI(3)P. The Wm resistance of glucose-starvation-induced autophagy was seen in HeLa cells, SKNSH cells, and MEFs. While we do not know why this is the case, given that one would expect VPS34 to be activated by upstream kinases under these conditions, one may speculate that it is not sufficient simply to activate the enzyme, but one also needs the enzyme to be in the right place, and perhaps the mechanism governing the correct localization of VPS34 to autophagosome precursors may be inefficient in glucose-starved cells.

Our observations may have therapeutic potential, because PIKfyve and PI5P4K2 enzymes are drug-treatable targets (Davis et al., 2013; Jefferies et al., 2008), and our data indicate that suppression of PI5P4K2’s activity increased the clearance of disease-associated autophagic substrates. Autophagy induction via other signaling effectors has benefits in a wide range of neurodegenerative diseases caused by aggregate-prone intracytoplasmic proteins, like Huntington’s and Parkinson’s diseases (Harris and Rubinsztein, 2011). Thus, PI5P4K2’s inhibition may provide a tractable therapeutic target for neurodegenerative diseases.

## Experimental Procedures

### Addition of Exogenous Lipids to Cells

Unlabeled PI, PI(5)P, PI(3)P, and PI (4)P di-C16; BODIPY-labeled PI(5)P and PI(3)P di-C6; and carrier (Echelon) were reconstituted in H2O:tert-BuOH (9:1) solution. After 1 min bath sonication, carrier and lipids were combined at a 1:1 ratio for 10 min at room temperature. The mixture of lipids and carrier was diluted in media and used for 1–2 hr incubations on cells. The final concentrations used were 0.1-1-10 μM. For the negative control; DMEM was combined with carrier only and added to the cells.

### Lipid Beads Pull-Down Assay

HeLa cells (stably expressing GFP alone or GFP-WIPI2B or transiently expressing WIPI2D) were suspended in lipid-binding buffer (20 mM Tris-HCl, 150 mM NaCl, and 1 mM EDTA [pH 7.5]). The cells were passed ten times through a G25 needle and sonicated on ice. After insoluble debris was removed by high-speed centrifugation at 13,000 × *g* for 1 hr at 4°C, a 50 μl slurry of PI, PI(3)P, PI(5)P, or unbound beads (Echelon Bioscience) was added to the tube and incubated for 4 hr at 4°C under rotary agitation. The beads were washed five times with lipid-wash buffer (10 mM HEPES [pH 7.4], 150 mM NaCl, 0.25% NP40), and the bound proteins were subjected to immunoblotting. For competitive inhibition with liposomes, cell extracts were incubated on ice with 50 μM PS/PI(5)P, PS/PI(3)P liposomes for 3 hr before the addition of PI(5)P or PI(3)P beads. Preincubation of the cell lysates with liposomes containing only PS was used as a control with the same negative net charge.

### SR-SIM

Samples were processed for conventional fluorescence microscopy and mounted on high-precision size 1.5 coverslips (Carl Zeiss). Coverslips were mounted with ProLong Gold antifade medium (P36934, Life Technologies), which was left to cure for 3 days at room temperature to produce samples with a consistent refractive index. SR-SIM was performed using an Elyra PS1 instrument (Carl Zeiss). Samples were examined on the microscope using a 63× 1.4NA plan-apo Carl Zeiss objective lens and Immersol 518F (23°C) immersion oil. Image acquisition was carried out using ZEN 2012 Elyra edition software, in which data sets were collected with five grating phases, five rotations, and sufficient z positions spaced 110 nm apart to form an approximately 2 μm deep volume of raw SR-SIM data. Optimal grating frequencies were selected for each wavelength used. Structured illumination postprocessing was performed in ZEN using parameters determined by automated analysis of the data sets. Reconstructed images were then corrected for spherical and chromatic aberrations using channel alignment information, which was created using a 3D array of multispectral beads previously imaged with the same instrument settings. The average final image resolution was calculated to be 110 nm in x and y dimensions and 240 nm in the z dimension, which represents a 2-fold lateral and axial improvement in resolution compared to conventional microscopy. Final visualization and video production was performed in Volocity 6.3 software using isosurface rendering of selected cropped regions of the data sets.

### Statistics

Protein levels were expressed as arbitrary units and numbers of vesicles or aggregate formation were expressed as percentages from three independent experiments carried out in triplicate, and the error bars denote SEM. Significance levels for comparisons between two groups were determined with two-tailed Student’s t test (Microsoft Excel); ^∗^p < 0.05; ^∗∗^p < 0.01; ^∗∗∗^p < 0.001.

## Supplemental Information

Document S1. Supplemental Experimental Procedures, Figures S1–S7, and Table S1

Movie S1. PHD3X and LC3-Positive Structures Analyzed by SR-SIM Related to Figure 1. HeLa cells transfected with GFP-PHD3X and RFP-LC3 for 16 hr were starved in HBSS for 1 hr, processed for conventional fluorescence microscopy, and mounted on high-precision size 1.5 coverslips. Superresolution structured illumination microscopy (SR-SIM) was performed using an Elyra PS1 instrument (Carl Zeiss Ltd.) and image acquisition. Structured illumination postprocessing and 3D reconstruction were carried out using ZEN 2012 Elyra edition software. Final visualization and video production were performed in Volocity 6.3 software using iso-surface rendering of selected cropped regions of the data sets. Note that this rendering means that vesicles positive for green and red do not look yellow, but have green and red on the surface.

Movie S2. PHD3X and ATG16L1-Positive Structures Analyzed by SR-SIM Related to Figure 2. HeLa cells transfected with GFP-PHD3X and Strb-ATG16L1 for 16 hr were starved in HBSS for 1 hr, processed for conventional fluorescence microscopy, and mounted on high-precision size 1.5 coverslips. Superresolution structured illumination microscopy (SR-SIM) was performed using an Elyra PS1 instrument (Carl Zeiss Ltd.) and image acquisition, structured illumination postprocessing, and 3D reconstruction were carried out using ZEN 2012 Elyra edition software. Final visualization and video production were performed in Volocity 6.3 software using iso-surface rendering of selected cropped regions of the data sets. Note that this rendering means that vesicles positive for green and red do not look yellow, but have green and red on the surface.

Document S2. Article plus Supplemental Information
