# Supplementary material for: PI(5)P Regulates Autophagosome Biogenesis
Source: Mol Cell. 2015 Jan 22;57(2):219–34. doi: 10.1016/j.molcel.2014.12.007 (PMC4306530; doi:10.1016/j.molcel.2014.12.007)
Supplement: Document S1. Supplemental Experimental Procedures, Figures S1–S7, and Table S1 [file mmc1.pdf]

Molecular Cell, Volume *57*

Supplemental Information

PI(5)P Regulates Autophagosome Biogenesis

Mariella Vicinanza, Viktor I. Korolchuk, Avraham Ashkenazi,
Claudia Puri, Fiona M. Menzies, Jonathan H. Clarke, and David C. Rubinsztein

Supplemental Data

Figure S1

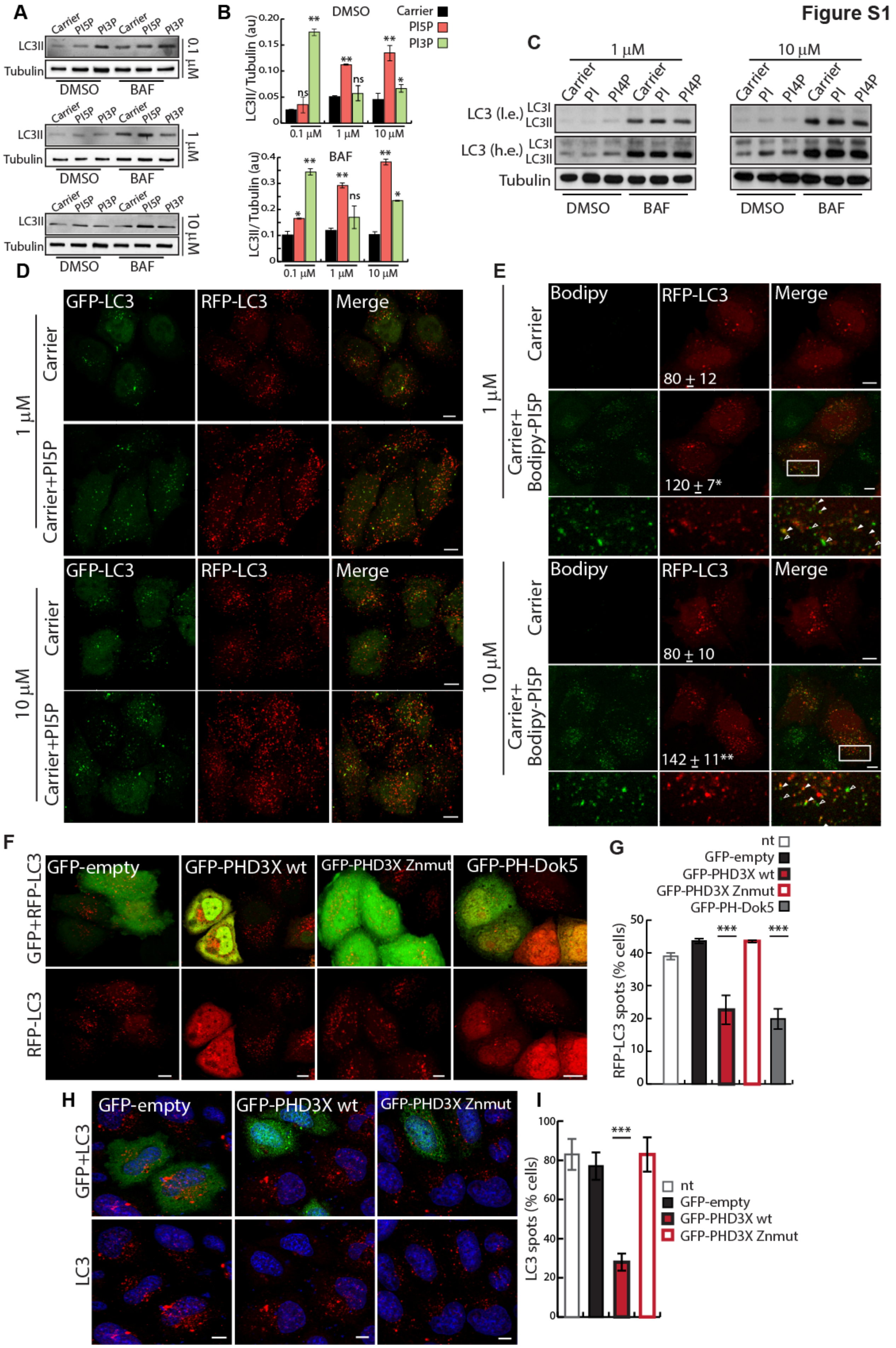

Figure S1: Maniplation of PI(5)P levels affects autophagosome numbers, Related to Figure 1. (**A**) Western blot analysis of LC3-II and tubulin levels in HeLa cells after 1h treatment with carrier alone or in combination with PI(5)P or PI(3)P di-C16 at indicated concentrations, in the absence and presence of 400 nM bafilomycin A1 (BAF, treated in combination with lipids for 1 h). (**B)**. Quantification of the band intensities from three experiments represented as LC3-II/tubulin ratio is shown in the graph; (mean±s.e.m). (**C**) Representative blots of LC3-II and tubulin levels in HeLa cells after 1h treatment with carrier alone or in combination with PI and PI(4)P di-C16 at indicated concentrations, in presence of 400 nM bafilomycin A1 (BAF, treated in combination with lipids for 1 h). Note that LC3-I is detectable with high exposure (h.e.). (**D**) HeLa cells stably expressing GFP-mRFP-LC3 were loaded with indicated concentrations of PI(5)P during which they were left in starvation media (HBSS). (**E**) HeLa cells transfected with RFP-LC3 were exposed to indicated concentrations of BODIPY-labelled-PI(5)P for 1h in starvation media (HBSS) and followed by live cell imaging for 10 min. For the negative control, medium was combined with carrier only and added to the cells. Numbers of RFP-LC3 structures per cell were quantified using the Analyse Particles plugin in ImageJ in at least 10 cells per condition from two independent experiments. (mean±s.e.m.). (**F,G**) HeLa cells transfected for 30 h with GFP-empty, GFP-PHD3X, GFP-PHD3X Znmut or GFP-PH-Dok5 combined with RFP-LC3, and were incubated in starvation media (HBSS) and fixed after 1 h. (**H,I**) HeLa cells transfected for 30 h with GFP-empty, GFP-PHD3X, GFP-PHD3X Znmut were then grown in HBSS for 1 h, after which the cells were fixed in methanol and immunostained for endogenous LC3 and anti-GFP. (**G,I**) Quantification of cells (% of total) showing more than 10 autophagic vesicles (LC3 or RFP-LC3 vesicles) in the different conditions is shown in the graph. nt, not transfected cells, n = 200 cells. (mean±s.e.m., t-test compared to GFP-transfected cells). Bar 10 μm.

Figure S2

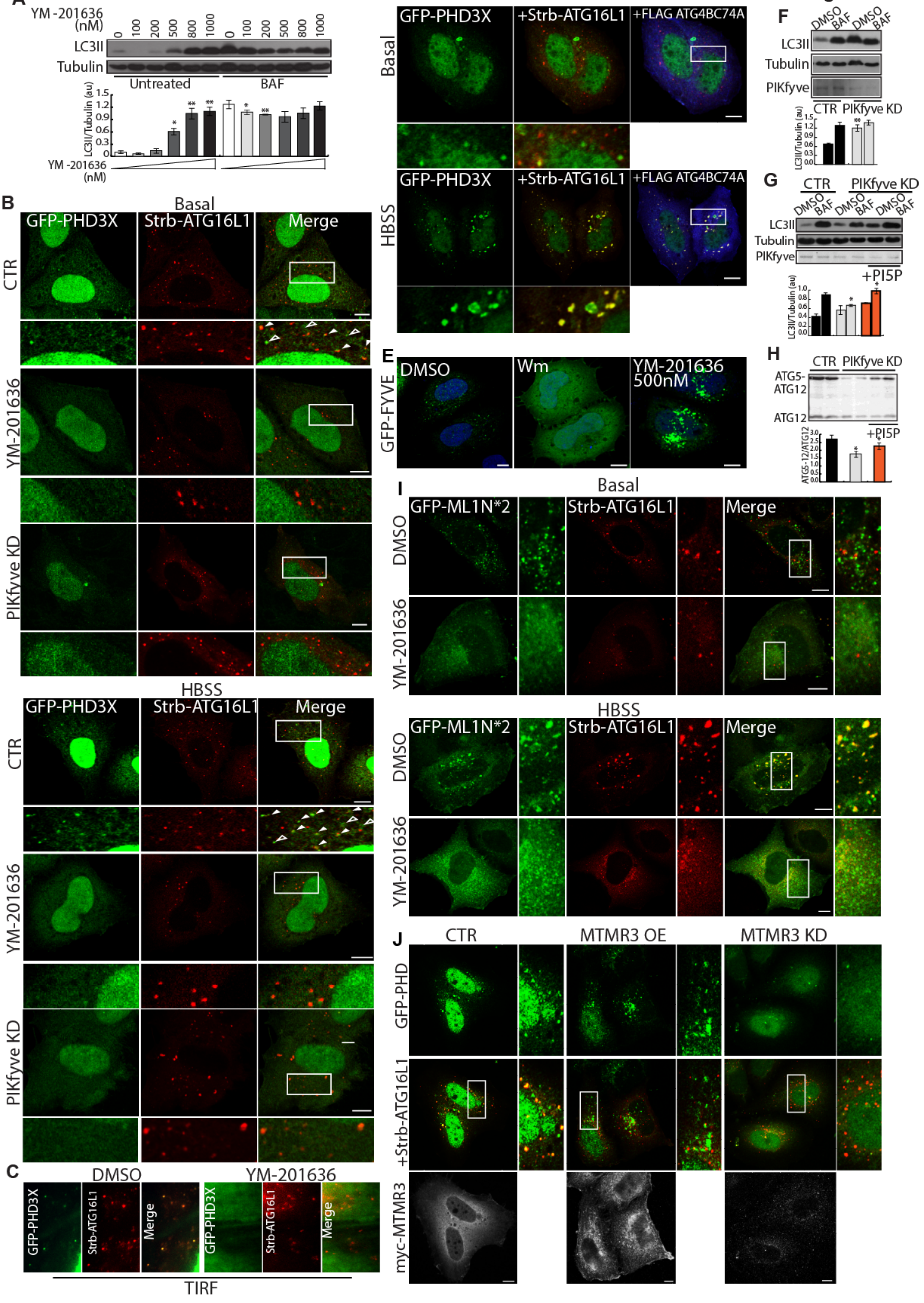

A
YM-201636 (nM)
0 100 200 500 800 1000 0 100 200 500 800 1000
LC3II
Tubulin
Untreated
BAF
LC3II/Tubulin (au)
YM-201636 (nM)
B
Basal
GFP-PHD3X
Strb-ATG16L1
Merge
CTR
YM-201636
PIKfyve KD
HBSS
C
DMSO
YM-201636
TIRF
D
GFP-PHD3X
+Strb-ATG16L1
+FLAG ATG4BC74A
Basal
HBSS
E
GFP-FYVE
DMSO
Wm
YM-201636 500nM
F
DMSO BAF DMSO BAF
LC3II
Tubulin
PIKfyve
CTR
PIKfyve KD
G
CTR
PIKfyve KD
+PI5P
H
ATG5-ATG12
ATG12
ATG5-12/ATG12
I
Basal
GFP-ML1N*2
DMSO
HBSS
J
CTR
MTMR3 OE
MTMR3 KD
GFP-PHD
+Strb-ATG16L1
myc-MTMR3

Figure S2: PIKfyve provides PI(5)P localized to autophagosomes, Related to Figure 2. (**A**) HeLa cells were treated with DMSO or increasing concentrations of YM-201636 for 24 h in the presence or absence of 400 nM BAF. Samples were prepared for SDS-PAGE and immunoblotted for LC3 and tubulin. The levels of LC3-II were normalised to tubulin and quantified (mean±s.e.m.). (**B**) HeLa cells transfected with GFP-PHD3X and Strawberry-ATG16L1 for 16 h were left in complete media (basal) or starvation media (HBSS) for 1h in presence or absence of 100 nM YM-201636, then fixed and imaged on confocal microscope. For PIKfyve silencing, cells were treated with PIKfyve siRNA for 5 days and transfected on day 4. (**C**) PI(5)P localization on ATG16L1-positive structures close to the plasma-membrane was analysed by TIRF imaging of GFP-PHD3X. Inhibition of PIKfyve activity by 100 nM YM-201636 caused loss of GFP-PHD3X dots. (**D**) HeLa cells transfected with flag-tagged ATG4BC74A, GFP-PHD3X and Strawberry-ATG16L1 for 16 h were left in complete media (basal) or starvation media (HBSS) for 1h, then fixed, stained with anti-flag antibodies and imaged on confocal microscope. (**E**) HeLa cells transfected with GFP-FYVE (a PI(3)P probe) for 16 h were treated with DMSO, 200nM Wm or 500 nM YM-201636 for 2 h. Wm treatment ablates the GFP-FYVE signal on vesicles, as a control. (**F-H**) HeLa cells were treated with control (CTR) or PIKfyve targeting siRNA for 5 days and treated with or without 200 nM BAF for 16 h in complete tissue culture medium (**F**) or for 2h in HBSS medium (**G**) or transfected with HA-ATG12 and ATG5 (**H**). In (**G**) and (**H**) cells were loaded with exogenous PI(5)P (10 μM) for 2h. Cells were subjected to western blot analysis with anti-LC3-II and anti-tubulin antibodies (LC3-II levels relative to tubulin were quantified) (**F-G)** and anti-HA antibody (to detect free ATG12 and the ATG5-ATG12 complex) **(H).** (**I**) HeLa cells transfected with GFP-ML1N*2 (tandem repeat of N-terminal segment of mucolipin1) and Strawberry-ATG16L1 for 16 h were left in complete media (basal) or starvation media (HBSS) for 1h in presence or absence of 100 nM YM-201636, then fixed and imaged on confocal microscope. Bar 10 μm. (**J**) HeLa cells transfected for 5 days with two rounds of control or MTMR3 siRNA were transfected with GFP-PHD3X, Strb-ATG16L1 and myc-tagged MTMR3 for last 12 h, starved for 1h in HBSS media and then fixed, labelled with anti-myc antibodies and imaged on confocal microscope. For MTMR3 over-expression, cells were transfected for 30 h. Bar 10 μm.

Figure S3

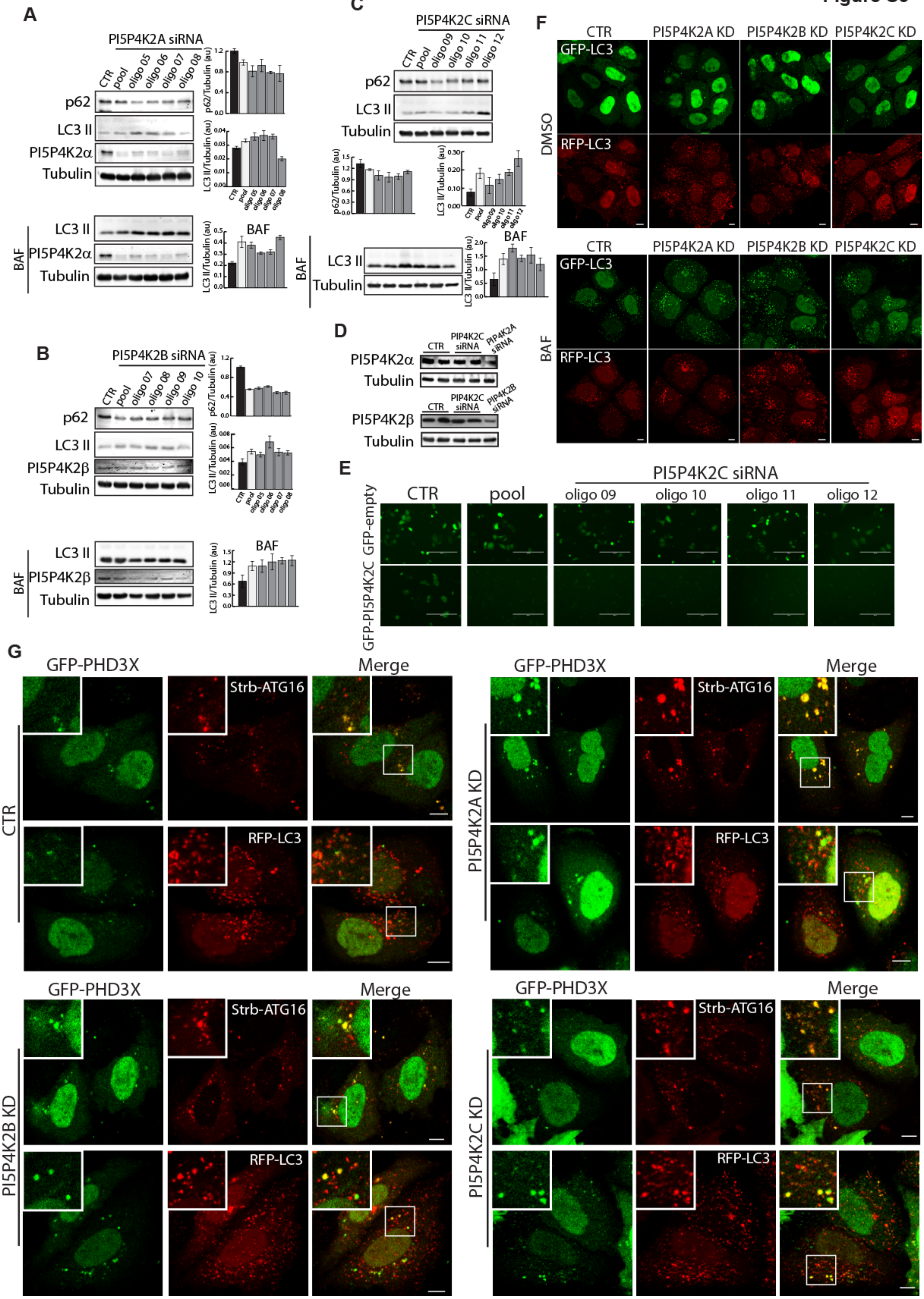

A
PI5P4K2A siRNA
CTR
pool
oligo 05
oligo 06
oligo 07
oligo 08
p62
LC3 II
PI5P4K2α
Tubulin
BAF
p62/Tubulin (au)
LC3 II/Tubulin (au)
B
PI5P4K2B siRNA
CTR
pool
oligo 07
oligo 08
oligo 09
oligo 10
PI5P4K2β
C
PI5P4K2C siRNA
oligo 09
oligo 10
oligo 11
oligo 12
D
PIP4K2C siRNA
PIP4K2A siRNA
PIP4K2B siRNA
E
GFP-empty
GFP-PI5P4K2C
F
PI5P4K2A KD
PI5P4K2B KD
PI5P4K2C KD
GFP-LC3
RFP-LC3
DMSO
G
GFP-PHD3X
Strb-ATG16
Merge
RFP-LC3
PI5P4K2A KD
PI5P4K2B KD
PI5P4K2C KD

Figure S3: PI5P4K2s knockdown affects PI(5)P levels on autophagosomes and clearance of autophagic substrates, Related to Figure 3. The specificity of PI5P4K2s KD effects on the levels of LC3-II and p62, were confirmed by deconvolution of PI5P4K2A, 2B and 2C siRNA smartpools. (**A-E**) HeLa cells were transfected with a set of 4 siRNA (pool) or individual siRNA oligonucleotides for 5 days and treated with or without 200 nM BAF for 16 h in complete tissue culture medium. Cells were immunoblotted and LC3-II and p62 levels relative to tubulin were quantified (graphs on the right). (**D**) PI5P4K2C silencing did not affect PI5P4K2A and PI5P4K2B levels. (**E**) The efficiency of PI5P4K2C knock-down was visualized by disappearance of GFP-tagged kinases using a fluorescence microscope (Evos FL imaging system) since a specific antibody was not available. Bar 200 μm. Note, decreased levels p62 in PI5P4K2s KD cells supports a functional role for PI(5)P and PI5P4K2s in autophagic substrate degradation. (**F**) HeLa cells stably expressing GFP–mRFP–LC3 were transfected for 5 days with two rounds of control, PI5P4K2A, 2B and 2C siRNA, during which they were either left untreated or treated with 200 nM BAF for the last 16 h. Cells were then fixed and analysed on a confocal microscope. (**G**) HeLa cells transfected with two rounds of control, PI5P4K2A, 2B and 2C siRNA for 5 days and with GFP-PHD3X, and RFP-LC3 or Strb-ATG16L1 for the last 16 h were incubated in HBSS for 1h and observed under a live cell confocal microscope. Bar 10 μm.

A

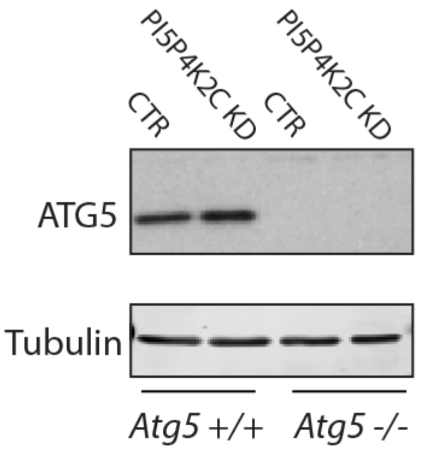

B

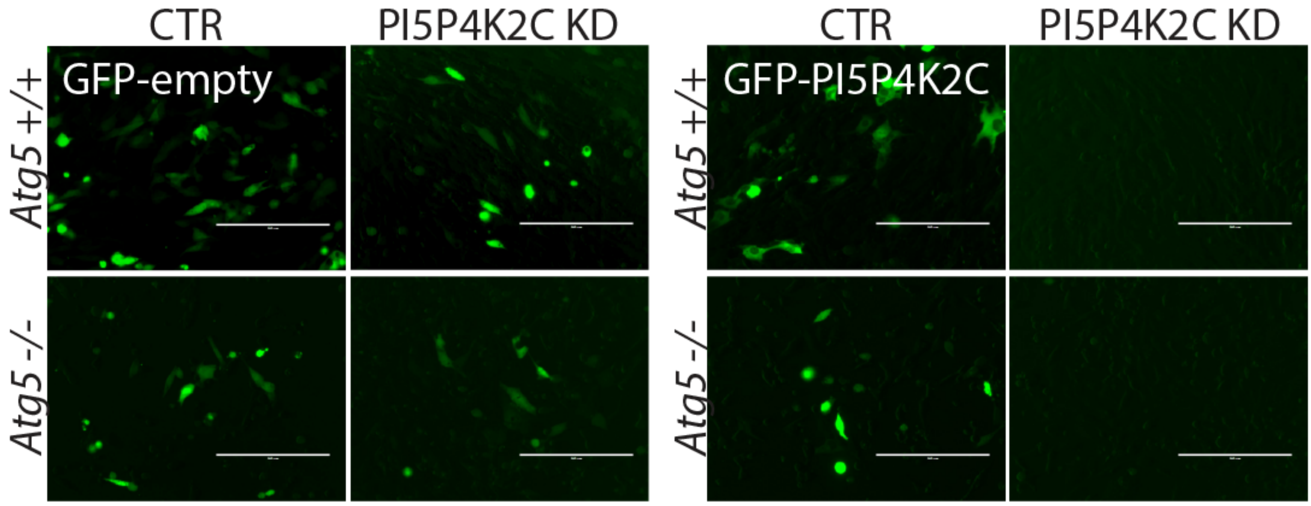

Figure S4: Increased PI(5)P levels accelerate autophagic substrate clearance, Related to Figure 4. *Atg5* $^{+/+}$ and *Atg5* $^{-/-}$ MEF cells were treated with PI5P4K2C siRNAs (oligo 10+oligo12) for 5 days and co-transfected with EGFP-httQ74 for the last 48 h. Western blot analysis of ATG5 levels are shown in (**A**) and efficiency of PI5P4K2C knock-down was visualized by disappearance of GFP-tagged kinases using a fluorescence microscope (Evos FL imaging system) (**B**). Bar 200 μm.

Figure S5

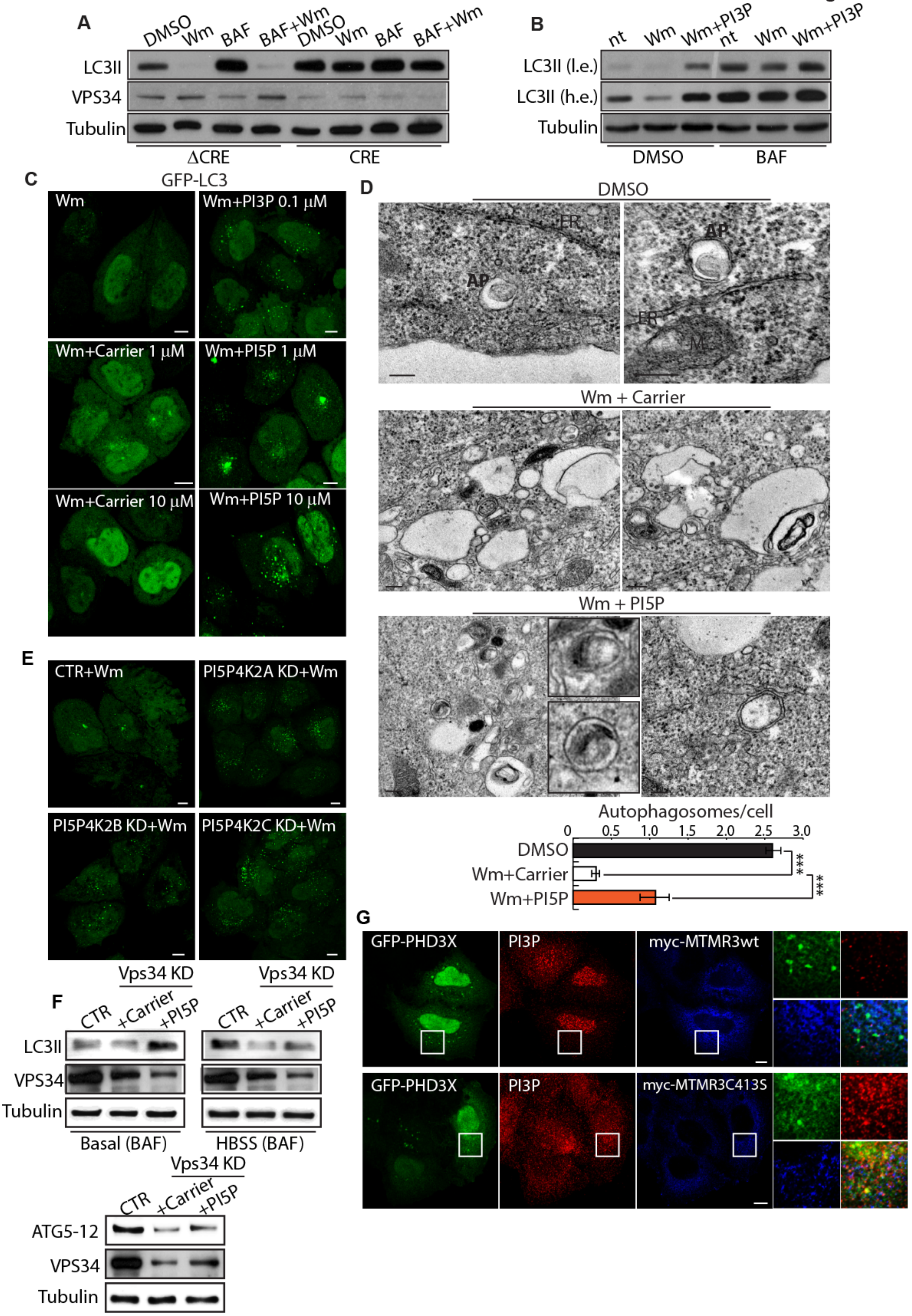
A
DMSO
Wm
BAF
BAF+Wm
DMSO
Wm
BAF
BAF+Wm
LC3II
VPS34
Tubulin
ΔCRE
CRE
B
nt
Wm
Wm+PI3P
nt
Wm
Wm+PI3P
LC3II (l.e.)
LC3II (h.e.)
Tubulin
DMSO
BAF
C
GFP-LC3
Wm
Wm+PI3P 0.1 μM
Wm+Carrier 1 μM
Wm+PI5P 1 μM
Wm+Carrier 10 μM
Wm+PI5P 10 μM
D
DMSO
ER
AP
AP
ER
M
Wm + Carrier
Wm + PI5P
Autophagosomes/cell
0
0.5
1.0
1.5
2.0
2.5
3.0
DMSO
Wm+Carrier
Wm+PI5P
***
***
E
CTR+Wm
PI5P4K2A KD+Wm
PI5P4K2B KD+Wm
PI5P4K2C KD+Wm
F
Vps34 KD
Vps34 KD
CTR
+Carrier
+PI5P
CTR
+Carrier
+PI5P
LC3II
VPS34
Tubulin
Basal (BAF)
HBSS (BAF)
Vps34 KD
CTR
+Carrier
+PI5P
ATG5-12
VPS34
Tubulin
G
GFP-PHD3X
PI3P
myc-MTMR3wt
GFP-PHD3X
PI3P
myc-MTMR3C413S

Figure S5: PI(5)P rescues autophagosome formation in Wm-treated cells, Related to Figure 5. (**A**) *Vps34*Flox/Flox MEFs were infected with lentiviruses either expressing full-length Cre recombinase (CRE) or not (Δ) for 12 days. Cells were incubated in HBSS in presence or absence of 200 nM Wm. Western blot analysis of LC3-II, tubulin and VPS34 levels are shown. (**B**) HeLa cells were loaded for 2 h with carrier alone, or combined with 0.1 μM PI(3)P, in presence of 200 nM Wm and DMSO or BAF in HBSS. (**C**) HeLa cells stably expressing GFP–LC3 were pre-treated with 200 nM Wm for 2h in complete medium, then loaded for 1 h with carrier alone, or combined with PI(5)P or PI(3)P at indicated concentrations, in presence of 200 nM Wm and then shifted to HBSS medium in the presence of Wm for 2 h and imaged on a confocal microscope. (**D**) HeLa cells were loaded for 2 h with carrier alone, or combined with 10 μM PI(5)P, in presence of 200 nM Wm in HBSS and processed for Electron-microscopy. Morphometrical analysis was performed counting autophagosome structures in 40 random cells profile for each condition in two independent experiments. AP, autophagosome, ER, endoplasmic reticulum, M, mitochondria. (***$p<0.001$, t-test), Bar 300nm. (**E**) HeLa cells stably expressing GFP–LC3 transfected for 5 days with two rounds of control, PI5P4K2A, 2B and 2C siRNA were pre-treated with 200 nM Wm for 2 h in complete medium and then shifted to HBSS medium in the presence of Wm for 2 h and imaged on a confocal microscope. Bar 10 μm. (**F**) HeLa cells transfected for 3 days with one round of control and VPS34 siRNA were loaded for 2h with carrier alone, or combined with 10 μM PI(5)P in presence of 400 nM BAF in complete medium or HBSS. Western blot analysis of LC3-II, tubulin, ATG12 and VPS34 protein levels are shown. (**G**) HeLa cells were transfected with GFP-PHD3X and myc-MTMR3wt or myc-MTMR3C314S, incubated for 1h in HBSS and processed for immunofluorescence analysis and labelling of PI(3)P (see Supplemental Experimental Procedures).

Figure S6

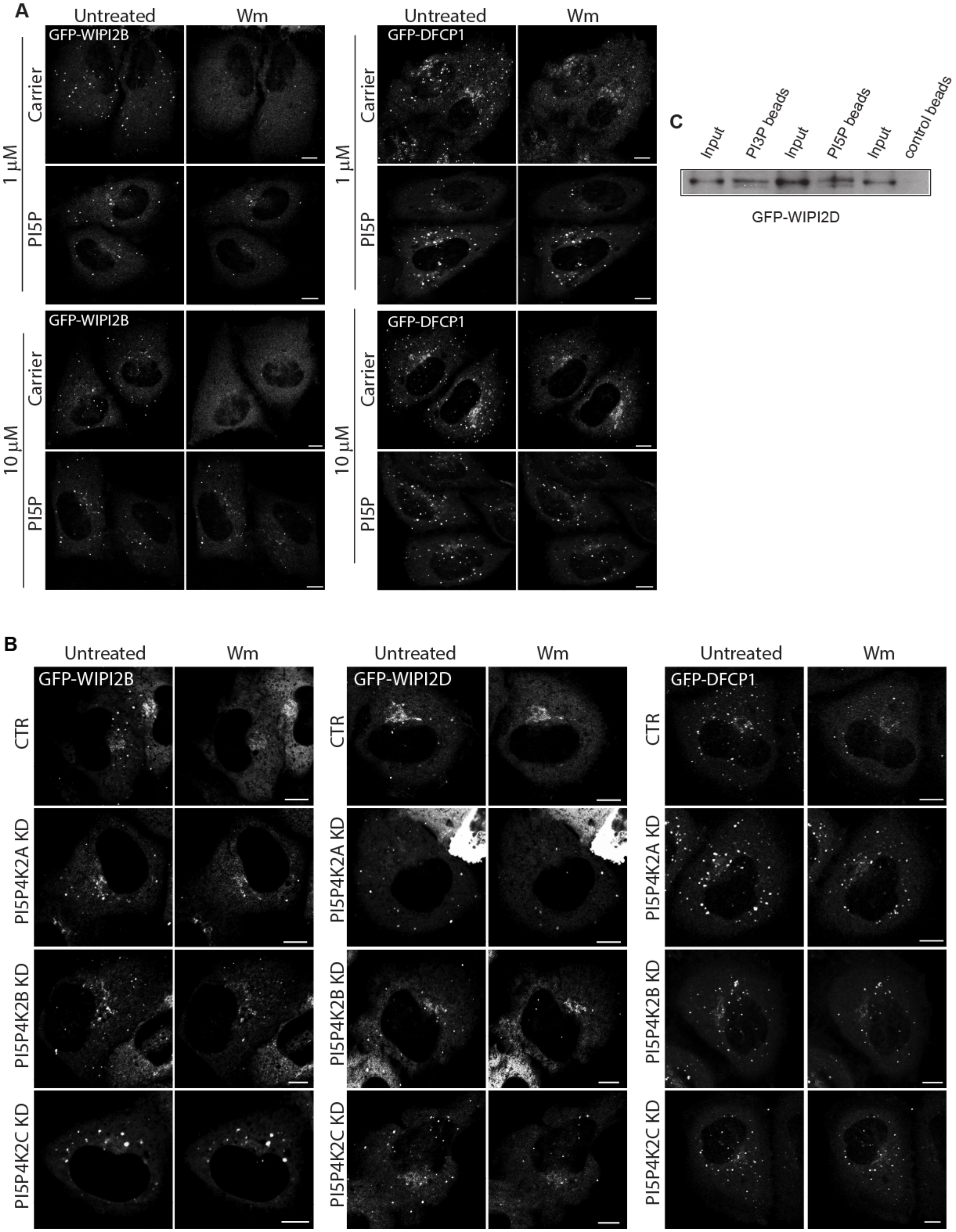
A
Untreated
Wm
GFP-WIPI2B
Carrier
PI5P
1 μM
10 μM
GFP-DFCP1
B
GFP-WIPI2B
GFP-WIPI2D
GFP-DFCP1
CTR
PI5P4K2A KD
PI5P4K2B KD
PI5P4K2C KD
C
Input
PI3P beads
Input
PI5P beads
Input
control beads
GFP-WIPI2D

Figure S6: PI(5)P stabilizes WIPI2 and DFCP1 on membranes in Wm-treated cells, Related to Figure 6. (**A**) HeLa cells transfected with GFP-WIPI2B or GFP-DFCP1 were loaded for 1 h with carrier alone or combined with PI(5)P at indicated concentrations and incubated with 200 nM Wm in HBSS media and structures tracked by time-lapse microscopy for 10 minutes after the addition of Wm. (**B**) HeLa cells transfected for 5 days with two rounds of control, PI5P4K2A, 2B and 2C siRNA were transfected with GFP-WIPI2B, GFP-WIPI2D and GFP-DFCP1 for the last 16 h, starved in HBSS media for 1h and incubated with 200 nM Wm in HBSS. Bar 10 μm. (**C**) Lysates from HeLa cells transiently expressing GFP-WIPI2D were incubated with agarose beads coated with PI, PI(5)P and PI(3)P, eluted with SDS-PAGE sample buffer and recovered proteins were assessed by western blotting using antibodies against GFP.

Figure S7

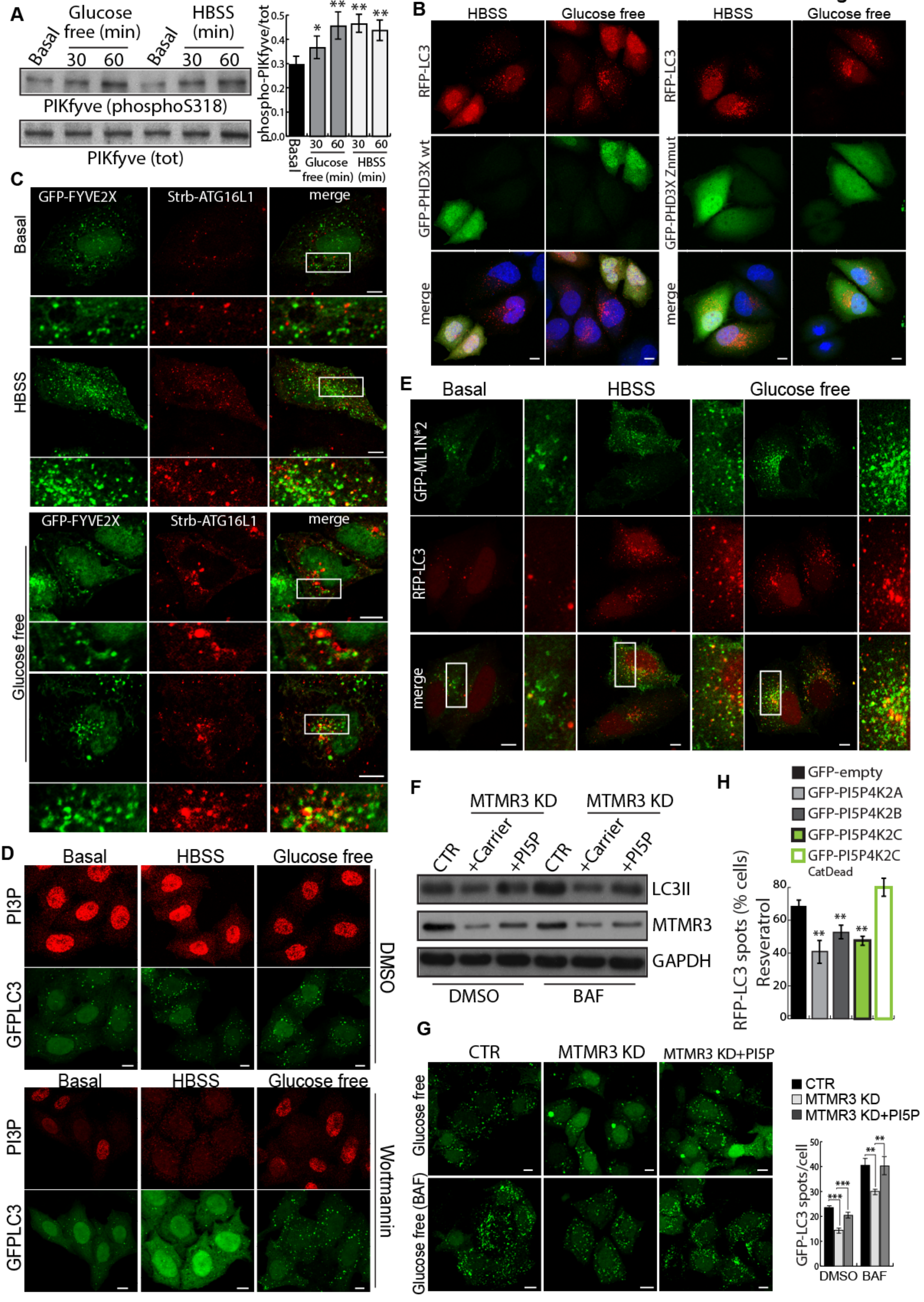
A
Basal
Glucose free (min)
30 60
Basal
HBSS (min)
30 60
PIKfyve (phosphoS318)
PIKfyve (tot)
phospho-PIKfyve/tot
Glucose free (min)
HBSS (min)
B
HBSS
Glucose free
RFP-LC3
GFP-PHD3X wt
GFP-PHD3X Znmut
merge
C
GFP-FYVE2X
Strb-ATG16L1
merge
Basal
HBSS
Glucose free
D
Basal
HBSS
Glucose free
PI3P
GFPLC3
DMSO
Wortmannin
E
GFP-ML1N*2
RFP-LC3
F
MTMR3 KD
CTR
+Carrier
+PI5P
LC3II
MTMR3
GAPDH
DMSO
BAF
G
CTR
MTMR3 KD
MTMR3 KD+PI5P
Glucose free (BAF)
GFP-LC3 spots/cell
H
GFP-empty
GFP-PI5P4K2A
GFP-PI5P4K2B
GFP-PI5P4K2C
GFP-PI5P4K2C CatDead
RFP-LC3 spots (% cells)
Resveratrol

Figure S7: PI(5)P is required for autophagosome formation during glucose starvation, Related to Figure 7. (**A**) MEF cells were incubated for indicated times in glucose free or HBSS media and Western blot analysis of PIKfyve phosphorylation was performed. Quantification of three independent experiments in shown (**p<0.01, t-test)**.** (**B**) HeLa cells transfected for 30 h with GFP-PHD3X and GFP-PHD3X Znmut combined with RFP-LC3 then were incubated in glucose-depleted media (Glucose free) and fixed after 4 h. (**C**) HeLa cells transfected with GFP-FYVE (a PI(3)P probe) combined with Strb-ATG16L1 for 16 h were incubated in HBSS or glucose-depleted media (Glucose free) and fixed after 4 h. (**D**) HeLa stably expressing GFP-LC3 were incubated in HBSS or glucose-depleted media (Glucose free), in presence or absence of 200 nM Wm, fixed after 4 h and labelled with PI(3)P antibodies. (**E**) HeLa cells transfected with GFP-ML1N*2 (tandem repeat of N-terminal segment of mucolipin1) and RFP-LC3 for 16 h were left in complete media (basal), starvation media (HBSS) or Glucose free DMEM (Glucose free) for 4h, then fixed and imaged on confocal microscope. Bar 10 μm. (**F**) Human neuroblastoma cells (SKSNH) cells were treated with MTMR3 siRNA for 5 days and loaded with carrier alone or combined with 10 μM PI(5)P in presence or absence of BAF in glucose free media. Western blot analysis of LC3-II, tubulin and MTMR3 is shown. (**G**) HeLa GFP-LC3 cells were treated with MTMR3 siRNA for 5 days and loaded with carrier alone or combined with 10 μM PI(5)P in presence or absence of BAF in glucose-free media. Quantification of numbers of autophagic vesicles per cell (GFP-LC3 vesicles) is shown in the graph (mean±s.e.m.). (**H**) HeLa cells transfected with GFP-PI5P4K2A, 2B, 2C or PI5P4K2C catalytic-dead and RFP-LC3 for 30 h were treated with 64 μM Resveratrol for 3 h and then fixed. Quantification of cells (% of total) showing more than 10 autophagic vesicles (RFP-LC3 vesicles) is shown in the graph. *n* = 100 cells. (mean±s.e.m.).

Table S1. siRNA oligo sequences used in this study, related to Figure 3, 4, 5, 6, 7, S2, S3, S4, S5, S6, S7.

name	sequence
PIP4K**2A** oligo05	gcccgauggucuuccguaa
PIP4K**2A** oligo06	gaacaucgacgucuaugga
PIP4K**2A** oligo07	gcauguaccggcuuaaugu
PIP4K**2A** oligo08	gaaaugcacaacauccuga
PIP4K**2B** oligo07	gacaaucaucucuucaaua
PIP4K**2B** oligo08	cccagccgcuuuaaguuua
PIP4K**2B** oligo09	ccucaaggguucuacgguu
PIP4K**2B** oligo10	gccagaaagugaagcuauu
PIP4K**2C** oligo09	gaaccuccgugaucgauuu
PIP4K**2C** oligo10	gcaguaugcuaagcgauuc
PIP4K**2C** oligo11	gaagagagauguggaguuu
PIP4K**2C** oligo11	gaagaaagcagcucaugca
**PIKfyve** oligo16	ucugagccauccugguuua
**PIKfyve** oligo15	gauggacguuggcuggauu
**PIKfyve** oligo14	gagaugaguaugcgcugua
**PIKfyve** oligo13	ggcacaagcuauagcaauu
**MTMR3** oligo06	gaccaaacguggacaguuc
**MTMR3** oligo07	gggcaggcauugagauaca
**MTMR3** oligo08	gcaaaguuaucagguguca
**MTMR3** oligo09	ugaaugccgagauauauuu
**VPS34** oligo09	caccaaagcucaucgacaa
**VPS34** oligo10	auagauagcucccaaauua
**VPS34** oligo11	gaacaacgguuucgcucuu
**VPS34** oligo12	gagauguacuugaacguaa

Supplemental Experimental Procedures

Plasmids. GFP-PHD3X and GFP-PHD3X Znmut were kindly provided by B. Payrastre (Inserm/UPS UMR 1048 - I2MC, Tolouse), GFP-PH-Dok5 by J. A. Nunes (Centre de Recherche en Cancerologie de Marseille), GFP-WIPI2B and GFP-WIPI2D by T. Proikas-Cezanne (Eberhard Karls University Tübingen), GFP-DFCP1 by N.T. Ktistakis (Babraham Institute, Cambridge, England, UK), ATG12-HA and ATG5 by N. Mizushima (The Tokyo Metropolitan Institute of Medical Science Japan), GFP-FYVE(EEA1) by H Stenmark (University of Oslo, Norway), GFP-ML1N*2 by H. Xu (University of Michigan, USA), myc-MTMR3wt and myc-MTMR3C413S by T. Noda (Osaka University) pmStrawberry-ATG16L1,peGFP-httQ74 and FLAG-ATG4BC74A have been described elsewhere (Cadwell et al., 2008; Korolchuk et al., 2011; Moreau et al., 2012), . mGFP-PI5P4K2A, mGFP-PI5P4K2B, mGFP-P5IP4K2C and PI5P4K2C-mGFP (N-term) were generated as previously described (Clarke et al., 2008). A kinase-dead mutant of mGFP-PI5P4K2C was generated by site-directed mutagenesis using the primer pairs 5'-CAGCTGAAGATCATGAAATACAGCCTTCTGCTAGGCATCC-3' (forward) and 5'-CCTAGCAGAAGGCTGTATTTCATGATCTTCAGCTGCACTA-3' (reverse) to convert the conserved ATP-binding residue Asp280 into a lysine. PI5P4K2C(D280K) was expressed as recombinant protein and assayed radiometrically to confirm loss of activity (data not shown). pCMV-PI5P4K2C (untagged) plasmid was from Origene.

WIPI2B FTTG mutants were generated by mutagenesis of the wild-type (FRRG) WIPI2B construct using QuikChange Site-Directed Mutatagenesis kit (Agilent Technologies), the primer sequence was GGACAAAAACTCTTTGAGTTTACGACAGGAGTAAAGAGGTGCGTG as described in(Dooley et al., 2014).

siRNA. ON-TARGETplus SMARTpool siRNA and individual siRNA oligonucleotides against human PI5P4K2A, PI5P4K2B, PI5P4K2C, MTMR3, PIKFyve, hVps34 and non-targeting SMARTpool siRNA (D-001810-04) were purchased from Dharmacon. PI5P4K2C knockdown in MEF cells was carried out using oligo 10+oligo 12 that match sequences in mouse PI5P4K2C. Oligos sequences are listed in Supplementary Table 1.

Cell lines and treatments. Human cervical carcinoma (HeLa) cells, human neuroblastoma (SKNSH) cells, human embryonic kidney (HEK293) cells, African green monkey kidney (COS7) cells, autophagy-related protein 5 (Atg5)-deficient ($Atg5^{-/-}$) and wild-type ($Atg5^{+/+}$) mouse embryonic fibroblast (MEF) cell lines (Kuma et al., 2004), were grown at 37°C in DMEM medium (Sigma) supplemented with 10% FBS, 100 U ml^{-1} penicillin/streptomycin, 2 mM l-glutamine and 1 mM sodium pyruvate (basal media), under 5% CO_2. HeLa cells stably expressing GFP-LC3 and HeLa cells stably expressing GFP–mRFP–LC3 were cultured in basal media supplemented with 500 µg/ml G418 (Sigma) (Kimura et al., 2007) or 200 µg/ml hygromycin B (Sigma), respectively. Stable HeLa cells clones expressing GFP-empty or GFP-WIPI2B were selected and cultured in basal media supplemented with 500 µg/ml G418 (Sigma). Vps34 Flox/Flox MEF were kindly provided by P.Codogno (INSERM U1151-CNRS UMR 8253, Paris). Control Vps34Flox/Flox (Control) and *Vps34-/-* (KO) cells were generated by infecting cells with a lentivirus, produced as previously described (Moffat et al., 2006) carrying or not (control) a catalytically active Cre recombinase. The cells were seeded at 1–2 × 10^5 per well in six-well plates, and transfection was performed with TransIT 2020 Mirus (for DNA) or LipofectAMINE 2000 (for siRNA and double transfections with DNA and siRNA) reagents (Invitrogen), using the manufacturer's protocol. ATG16L1-mStrawberry was transfected at 0.3µg per well, pEGFP-httQ74 at 0.5 µg and the other constructs at 1µg. Cells were transfected for 16 h for localization experiments and 30 h for overexpression experiments. Final siRNA concentrations of 50 or 100nM were used for silencing with two rounds of knockdown for 5 days. In some experiments, cells were starved in Hanks balanced salt solution (HBSS) media (Invitrogen) or treated for 1-4 h with 400 nM or 16 h with 200 nM bafilomycin A (Sigma), for 2-4 h with 200 nM Wortmannin (Sigma), for 1-4 h with different concentrations of YM-201636 (Cayman Chemical) or for 3h with 64 µM Resveratrol (Sigma).

HBSS contains 1g/l of D-glucose; DMEM contains 4.5 g/l glucose; and DMEM without glucose has 0g/l glucose. Glucose starvation was carried by culturing cells in DMEM lacking glucose (Invitrogen, #11966) with 10% dialyzed FBS and glutamine for 4 h.

Preparation of liposomes. Thin films were generated following dissolution of the lipids in a 2:1 (v/v) chloroform/methanol mixture and then dried under a stream of argon gas, while they were rotated. Lipids were mixed at the appropriate ratios to preserve the same net charge on liposomes by controlling the 1-palmitoyl-2-oleoyl-sn-glycero-3-phospho-L-serine (PS) molar fraction. The charges were assumed to be: PS -1; PI(3)P or PI(5)P -3. The final compositions in mole percentage of the different liposome population were: PI(3)P-containing liposomes: 15% of dipalmitoyl phosphatidylinositol 3-phosphate (PI(3)P), 30% PS, 55% of 1-palmitoyl-2-oleoyl-sn-glycero-3-phosphocholine (PC); PI(5)P-containing liposomes: 15% of dipalmitoyl phosphatidylinositol 5-phosphate (PI(5)P), 30% PS, 55% PC; PS-containing liposomes: 75% PS, 25% PC. The films were lyophilized overnight, and the containers were sealed with argon gas to prevent oxidation and stored at −20 °C. Before an experiment, the films were suspended in the lysis buffer used for the lipid binding experiment and vortexed for 1.5 min. The lipid suspension underwent five cycles of freezing and thawing followed by extrusion through polycarbonate membranes with 1 and 0.1 μm diameter pores 21 times to create large unilamellar vesicles.

Western blot analysis. Cells were lysed in Laemmli buffer and protein samples were boiled for 5–7 min at 100°C, separated by SDS-PAGE, transferred onto PVDF membranes, subjected to western blot analysis, and, finally visualized using an ECL enhanced chemiluminescence detection kit (GE Healthcare), or with direct infrared fluorescence detection on an Odyssey Infrared Imaging System. The primary antibodies used include anti- tubulin (1:5,000; Sigma Aldrich), GAPDH (1:3000, Abcam), anti-GFP (1:2000, Clontech), anti-ATG5 (1:5,000; Sigma Aldrich), anti-ATG12 (1:1000, Cell Signaling), anti-LC3 (1:4,000; Novus Biologicals), anti-HA (1:1000, Covance), anti-p62 (1:1000, MBL), anti-PI5P4K2A (1:500, Abcam), anti-PI5P4K2B (1:500, Abcam), anti-PIKfyve and anti-phospho-PIKfyve (1:500, Abcam), anti-MTMR3 (1:500, Cell Signaling), anti-VPS34 (1:1000, Cell Signaling).

Immunofluorescence. Cells were grown on coverslips at 25% confluency were fixed in 4% paraformaldehyde (for endogenous WIP1-2, 1:100 Abcam, for myc tag, 1:500 Abcam, for flag tag, 1:500 Sigma Aldrich) or methanol (for endogenous LC3, 1:100 Novus Biologicals and anti-GFP, 1:400 Abcam) for 5 min and then permeabilized with 0.1% Triton. 1% BSA in PBS was used for blocking and primary and secondary antibodies. A Zeiss LSM710 confocal microscope was used for fluorescent confocal analysis. All confocal images were taken with a 63X oil-immersion lens. ImageJ (number of vesicles analysis) were used for further analysis and processing of confocal images.

Staining of PI(3)P for immunofluorescence

The staining of PtdIns(3)P was performed as described by Hammond et al. (2009). Briefly, cells were fixed in 2% paraformaldehyde and permeabilized with 20 μM digitonin in buffer A (20 mM Pipes pH 6.8, 137 mM NaCl, 2.7 mM KCl). Then cells were blocked with buffer A supplemented with 5% (v/v) FBS and 50 mM NH4Cl. Anti-PtdIns(3)P antibodies from Echelon (for 1 h, 1:300) and secondary antibodies were applied in buffer A with 5% FBS. Cells underwent post-fixation for 5 min in 2% paraformaldehyde, washed with PBS containing 50 mM NH4Cl, washed once with water and then mounted with Mowiol.

TIRF microscopy. HeLa cells were seeded on 35-mm MatTek glass-bottomed Petri dishes at a density of about 1.5 × 10^5 cells per dish. Cells were transfected for 16 h with relevant reagents, after which they were cultured in HBSS for a further 2 h, and treated with 100 nM YM-201636 for the last 1 h and fixed. Imaging was performed on a Zeiss TIRF 3 system using an αPlan-Fluor 100× 1.45 numerical aperture oil-immersion lens under the control of the AxioVision software. Laser lines at 488 and 561 nm were used for GFP–PHD3X and Strawberry-ATG16L1 excitation, respectively.

Electron microscopy. HeLa cells were loaded for 2 h with carrier alone, or combined with 10 μM PI(5)P, in presence of 200 nM Wm in HBSS and then fixed with a mixture of 2% paraformaldehyde and 2% glutaraldehyde in cacodylate buffer 0.1M (pH. 7.4) for 1 h, at room temperature. Cells were then post-fixed in Osmium tetroxide 1% in cacodylate buffer 0.1M (pH. 7.4) and processed for Epon embedding. Epon

sections were cut using REICHERT ultracut S and analysed with Philips CM100 electron microscope. Morphometrical analysis was performed counting autophagosome structures in 40 random cells profile for each condition in two independent experiments.

Autophagy analyses. Measurement methods for the levels of endogenous LC3-II/tubulin ratios as a read-out for autophagosome numbers have been previously described (Sarkar et al., 2009). To quantify LC3-positive vesicles, cells were transfected with an RFP-LC3 plasmid or were fixed and stained with an anti-LC3II antibody. We determined the percentages of cells with more than 10 LC3-positive vesicles. All experiments were carried out in triplicate with at least 200 cells counted per slide; the scorer was blinded to treatment. Automated microscope counting of total autophagosomes (in HeLa stable expressing GFP-LC3) or autolysosomes labelled with a pH-sensitive mRFP-GFP-LC3 (also called tfLC3) was carried out using a Thermo Scientific Cellomics ArrayScan VTI HCS reader and the Spot Detector Bioapplication protocol, as described (Sarkar et al., 2009). With tfLC3, GFP- (and mRFP-) positive puncta represent autophagosomes before lysosomal fusion, whereas mRFP-positive puncta (that lack GFP fluorescence) represent autolysosomes (as the GFP is more rapidly quenched by the low pH(Kimura et al., 2007)). At least 1000 cells per condition in three independent experiments were used for quantification. Alternatively, total numbers of GFP-RFP and RFP-only positive puncta per cell were quantified from confocal images using ImageJ and the Analyse Particles plugin (a constant threshold for all of the images within each experiment was applied). At least 20 cells per condition in three independent experiments were used for quantification.

ATG5-ATG12 conjugation was detected in cells transfected with HA-ATG12 and ATG5, lysed and subjected to western blot analysis with anti-HA antibody, so we could assess free ATG12 and the ATG5-ATG12 complex formed by the transfected constructs. We determined the ratio of ATG5-ATG12 versus free ATG12.

EGFP-httQ74 aggregation was detected by direct immunofluorescence. The proportion of transfected cells with aggregates was scored (approx. 500 cells per coverslip). Experiments were performed blinded and in triplicate in at least three independent experiments. Statistics for aggregation assays were calculated as odds ratios (the ratio of cells containing aggregates in each condition).

References

Cadwell, K., Liu, J.Y., Brown, S.L., Miyoshi, H., Loh, J., Lennerz, J.K., Kishi, C., Kc, W., Carrero, J.A., Hunt, S.*, et al.* (2008). A key role for autophagy and the autophagy gene Atg16l1 in mouse and human intestinal Paneth cells. Nature *456*, 259-263.

Clarke, J.H., Emson, P.C., and Irvine, R.F. (2008). Localization of phosphatidylinositol phosphate kinase IIgamma in kidney to a membrane trafficking compartment within specialized cells of the nephron. Am J Physiol Renal Physiol *295*, F1422-1430.

Dooley, H.C., Razi, M., Polson, H.E., Girardin, S.E., Wilson, M.I., and Tooze, S.A. (2014). WIPI2 links LC3 conjugation with PI3P, autophagosome formation, and pathogen clearance by recruiting Atg12-5-16L1. Mol Cell *55*, 238-252.

Hammond, G.R., Schiavo, G., and Irvine, R.F. (2009). Immunocytochemical techniques reveal multiple, distinct cellular pools of PtdIns4P and PtdIns(4,5)P(2). Biochem J *422*, 23-35.

Kimura, S., Noda, T., and Yoshimori, T. (2007). Dissection of the autophagosome maturation process by a novel reporter protein, tandem fluorescent-tagged LC3. Autophagy *3*, 452-460.

Korolchuk, V.I., Saiki, S., Lichtenberg, M., Siddiqi, F.H., Roberts, E.A., Imarisio, S., Jahreiss, L., Sarkar, S., Futter, M., Menzies, F.M.*, et al.* (2011). Lysosomal positioning coordinates cellular nutrient responses. Nat Cell Biol *13*, 453-460.

Kuma, A., Hatano, M., Matsui, M., Yamamoto, A., Nakaya, H., Yoshimori, T., Ohsumi, Y., Tokuhisa, T., and Mizushima, N. (2004). The role of autophagy during the early neonatal starvation period. Nature *432*, 1032-1036.

Moffat, J., Grueneberg, D.A., Yang, X., Kim, S.Y., Kloepfer, A.M., Hinkle, G., Piqani, B., Eisenhaure, T.M., Luo, B., Grenier, J.K.*, et al.* (2006). A lentiviral RNAi library for human and mouse genes applied to an arrayed viral high-content screen. Cell *124*, 1283-1298.

Moreau, K., Ravikumar, B., Puri, C., and Rubinsztein, D.C. (2012). Arf6 promotes autophagosome formation via effects on phosphatidylinositol 4,5-bisphosphate and phospholipase D. J Cell Biol *196*, 483-496.
