# Supplementary figures and images for: PI(5)P Regulates Autophagosome Biogenesis
Source: Mol Cell. 2015 Jan 22;57(2):219–34. doi: 10.1016/j.molcel.2014.12.007 (PMC4306530; doi:10.1016/j.molcel.2014.12.007)

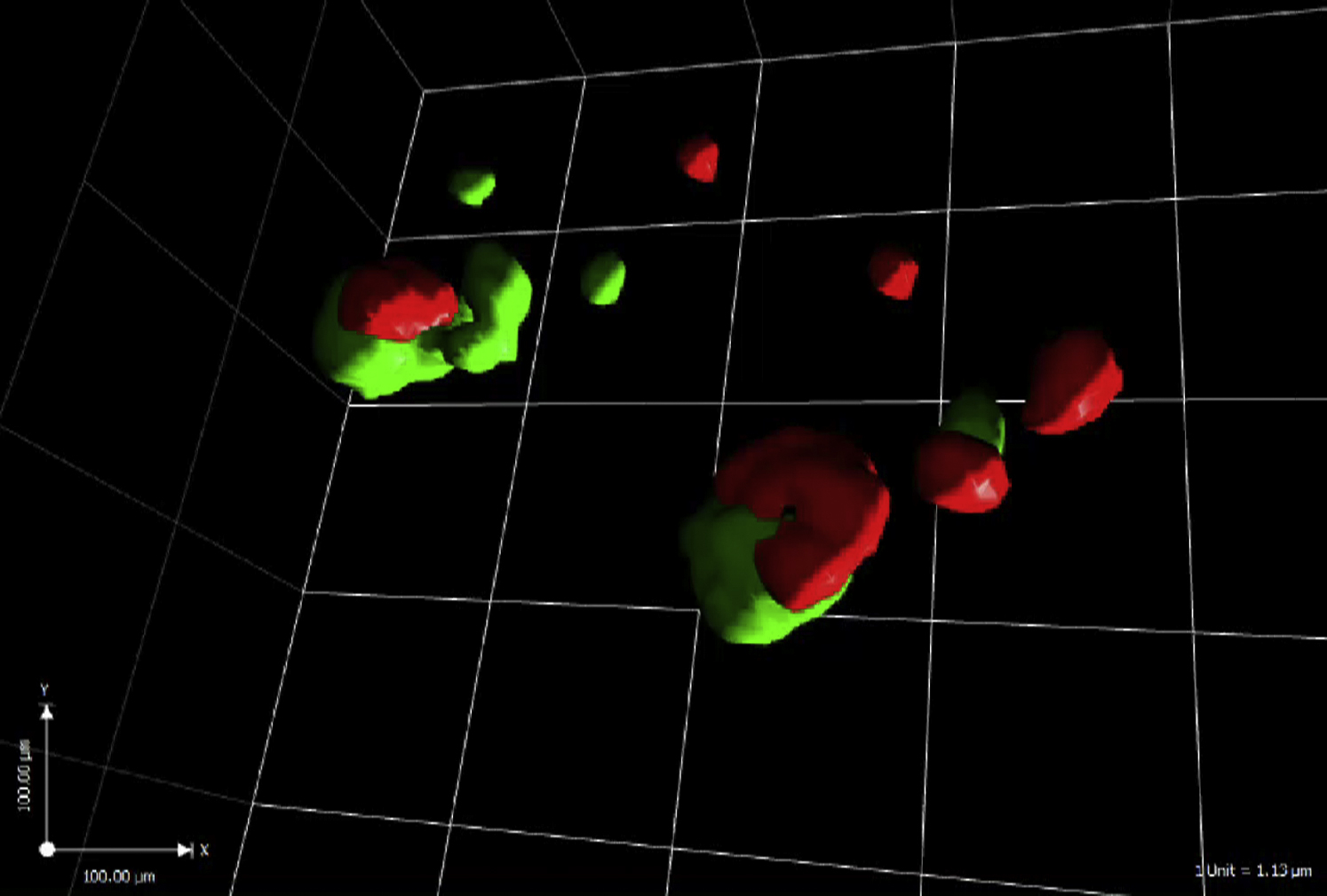

Supplement: Movie S1. PHD3X and LC3-Positive Structures Analyzed by SR-SIM — Related to Figure 1. HeLa cells transfected with GFP-PHD3X and RFP-LC3 for 16 hr were starved in HBSS for 1 hr, processed for conventional fluorescence microscopy, and mounted on high-precision size 1.5 coverslips. Superresolution structured illumination microscopy (SR-SIM) was performed using an Elyra PS1 instrument (Carl Zeiss Ltd.) and image acquisition. Structured illumination postprocessing and 3D reconstruction were carried out using ZEN 2012 Elyra edition software. Final visualization and video production were performed in Volocity 6.3 software using iso-surface rendering of selected cropped regions of the data sets. Note that this rendering means that vesicles positive for green and red do not look yellow, but have green and red on the surface. [file mmc2.jpg]

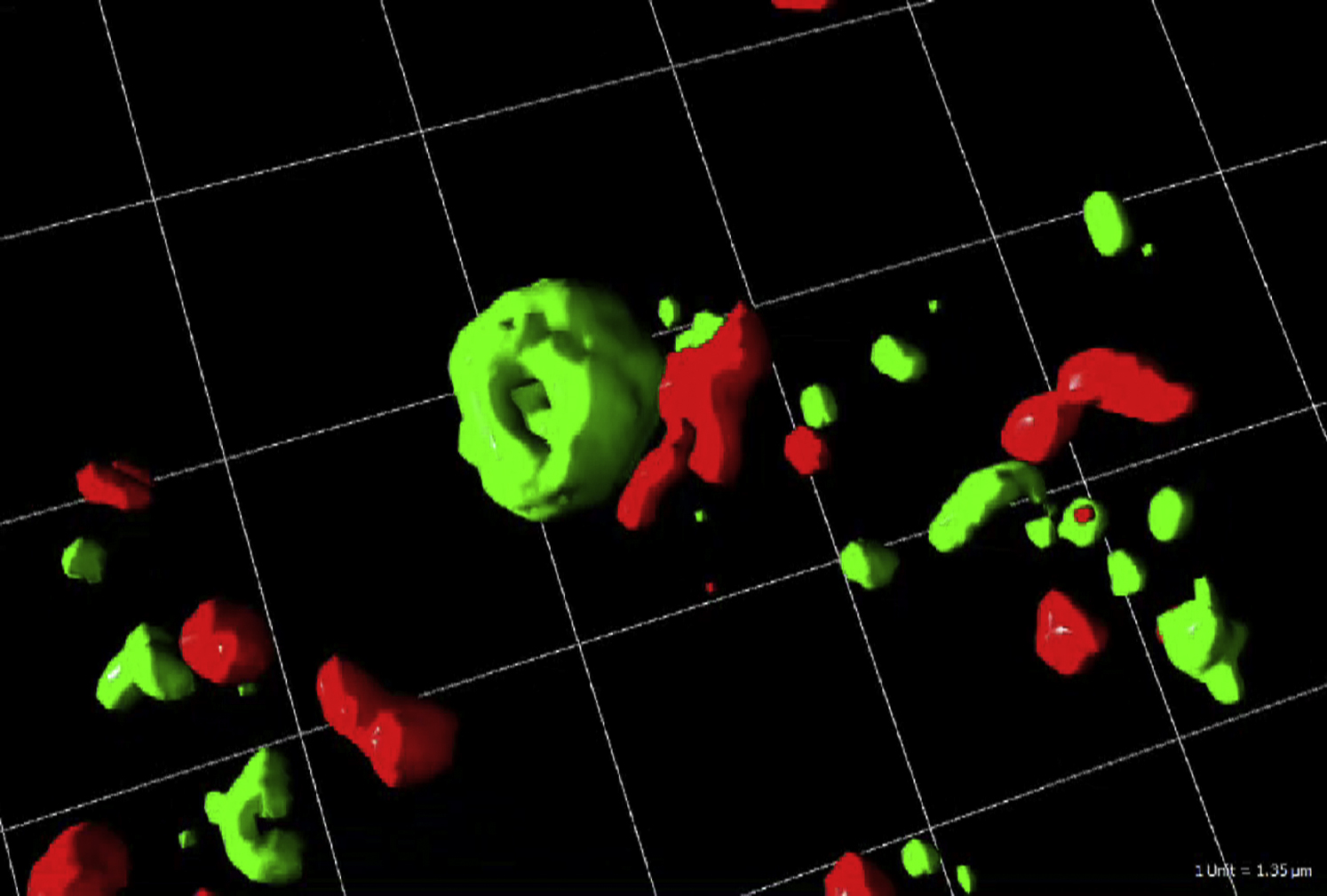

Supplement: Movie S2. PHD3X and ATG16L1-Positive Structures Analyzed by SR-SIM — Related to Figure 2. HeLa cells transfected with GFP-PHD3X and Strb-ATG16L1 for 16 hr were starved in HBSS for 1 hr, processed for conventional fluorescence microscopy, and mounted on high-precision size 1.5 coverslips. Superresolution structured illumination microscopy (SR-SIM) was performed using an Elyra PS1 instrument (Carl Zeiss Ltd.) and image acquisition, structured illumination postprocessing, and 3D reconstruction were carried out using ZEN 2012 Elyra edition software. Final visualization and video production were performed in Volocity 6.3 software using iso-surface rendering of selected cropped regions of the data sets. Note that this rendering means that vesicles positive for green and red do not look yellow, but have green and red on the surface. [file mmc3.jpg]
