# Supplementary material for: PI(5)P Regulates Autophagosome Biogenesis
Source: Mol Cell. 2015 Jan 22;57(2):219–34. doi: 10.1016/j.molcel.2014.12.007 (PMC4306530; doi:10.1016/j.molcel.2014.12.007)
Supplement: Document S2. Article plus Supplemental Information [file mmc4.pdf]

Molecular Cell

Article

PI(5)P Regulates Autophagosome Biogenesis

Graphical Abstract

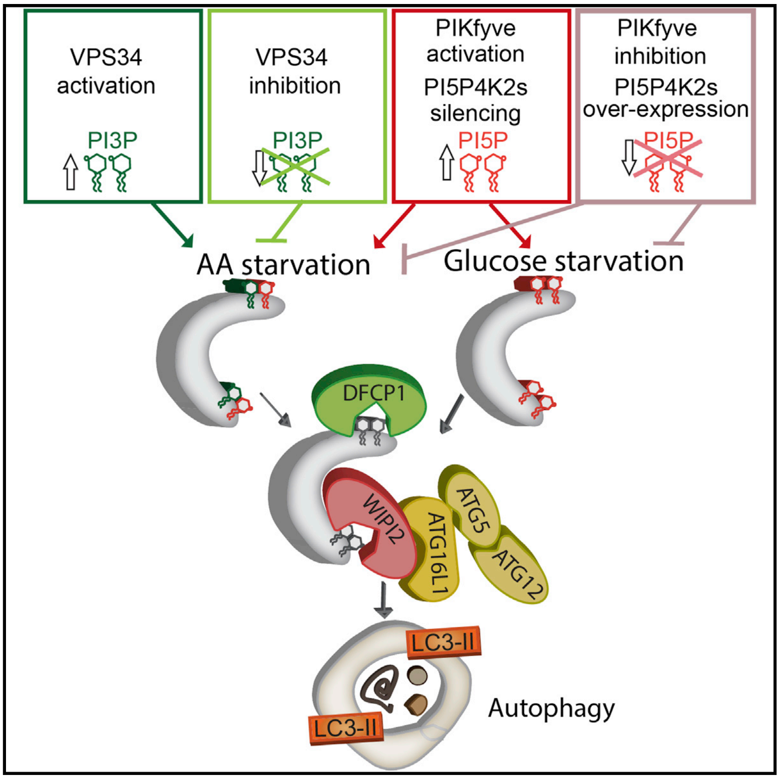

Highlights

- PI(5)P positively regulates autophagy
- PI(5)P is associated with autophagy effectors that bind PI(3)P
- PI(5)P sustains noncanonical autophagy in PI(3)P-depleted cells
- PI(5)P is essential for VPS34-independent, glucose-starvation-induced autophagy

Authors

Mariella Vicinanza, Viktor I. Korolchuk, ..., Jonathan H. Clarke, David C. Rubinsztein

Correspondence

dcr1000@cam.ac.uk

In Brief

PI(3)P, the product of VPS34, regulates canonical autophagy; however, mammalian cells can produce autophagosomes through enigmatic noncanonical VPS34-independent pathways. Vicinanza et al. show that PI(5)P can regulate autophagy, even in cells where VPS34 is compromised and acts via PI(3)P effectors. This provides a mechanistic explanation for forms of noncanonical autophagy.

Vicinanza et al., 2015, Molecular Cell *57*, 219–234
January 22, 2015 ©2015 The Authors
http://dx.doi.org/10.1016/j.molcel.2014.12.007

CellPress

PI(5)P Regulates Autophagosome Biogenesis

Mariella Vicinanza,[1] Viktor I. Korolchuk,[1,3] Avraham Ashkenazi,[1] Claudia Puri,[1] Fiona M. Menzies,[1] Jonathan H. Clarke,[2] and David C. Rubinsztein[1,*]
[1]Department of Medical Genetics, Cambridge Institute for Medical Research, Wellcome/MRC Building, Cambridge Biomedical Campus, Hills Road, Cambridge CB2 0XY, UK
[2]Department of Pharmacology, University of Cambridge, Tennis Court Road, Cambridge CB2 1PD, UK
[3]Present address: Institute for Cell and Molecular Biosciences, Campus for Ageing and Vitality, Newcastle University, Newcastle upon Tyne NE4 5PL, UK
*Correspondence: dcr1000@cam.ac.uk
http://dx.doi.org/10.1016/j.molcel.2014.12.007
This is an open access article under the CC BY license (http://creativecommons.org/licenses/by/3.0/).

SUMMARY

Phosphatidylinositol 3-phosphate (PI(3)P), the product of class III PI3K VPS34, recruits specific autophagic effectors, like WIPI2, during the initial steps of autophagosome biogenesis and thereby regulates canonical autophagy. However, mammalian cells can produce autophagosomes through enigmatic noncanonical VPS34-independent pathways. Here we show that PI(5)P can regulate autophagy via PI(3)P effectors and thereby identify a mechanistic explanation for forms of noncanonical autophagy. PI(5)P synthesis by the phosphatidylinositol 5-kinase PIKfyve was required for autophagosome biogenesis, and it increased levels of PI(5)P, stimulated autophagy, and reduced the levels of autophagic substrates. Inactivation of VPS34 impaired recruitment of WIPI2 and DFCP1 to autophagic precursors, reduced ATG5-ATG12 conjugation, and compromised autophagosome formation. However, these phenotypes were rescued by PI(5)P in VPS34-inactivated cells. These findings provide a mechanistic framework for alternative VPS34-independent autophagy-initiating pathways, like glucose starvation, and unravel a cytoplasmic function for PI(5)P, which previously has been linked predominantly to nuclear roles.

INTRODUCTION

Macroautophagy (henceforth autophagy) is a cellular process that delivers damaged organelles, invasive bacteria, and long-lived or aggregate-prone proteins to lysosomes for degradation (Boya et al., 2013). These substrates are engulfed along with bulk cytoplasm by double-membraned, cup-shaped phagophores, which form autophagosomes after their edges extend and fuse. After autophagosome-lysosome fusion, the resulting degradation products are recycled back to the cytosol and are reused to enhance cell survival during nutrient deprivation. Autophagy impacts the pathogenesis of diverse diseases, including neurodegenerative conditions, cancers, and infectious diseases (Nixon, 2013).

Many proteins (ATGs) regulate the membrane remodelling and trafficking events in autophagy, but comparatively little is known about the roles of lipids and their metabolizing enzymes in this process (Nakatogawa et al., 2009). Phosphoinositides (PIs) are low-abundance lipids that are interconverted by highly regulated sets of PI kinases and phosphatases. The interconvertibility of PIs enables rapid local changes in the identity of intracellular compartments to dynamically recruit effector proteins to specific membranes at the right time. The class III phosphatidylinositol 3-kinase (also known as VPS34) and its product phosphatidylinositol 3-phosphate (PI(3)P) are critical for autophagosome formation (Boya et al., 2013; Nakatogawa et al., 2009). Local increases of PI(3)P recruits proteins associated with autophagy initiation, such as DFCP1 (double FYVE-containing protein 1) and the WIPI proteins (WD-repeat protein interacting with PI), of which WIPI2 has been characterized as an effector of autophagy (Polson et al., 2010).

While autophagy has been classically considered to be PI(3)P-dependent, noncanonical VPS34-independent autophagy has been reported (Codogno et al., 2012; Plowey et al., 2008; Scarlatti et al., 2008; Zhu et al., 2007), as autophagosomes are seen in T lymphocytes and sensory neurons from *Vps34*$^{-/-}$ mice (Zhou et al., 2010) and in glucose-starved cells treated with the VPS34 inhibitor Wortmannin (Wm) (McAlpine et al., 2013). However, it is unclear whether these phenomena may be explicable by VPS34-independent source(s) of PI(3)P (Devereaux et al., 2013) or by other PIs.

PI(5)P remains the most enigmatic of the PIs due to its low abundance (Shisheva, 2013). The pathways regulated by PI(5)P in mammalian cells and its effectors/binding proteins are still poorly understood. So far these include chromatin organization in the nuclei, bacteria invasion, and cytoskeletal remodelling (Shisheva, 2013). Here, we describe a role for PI(5)P as a regulator of autophagosome biogenesis that can also regulate autophagy when VPS34 is inhibited.

RESULTS

PI(5)P Regulates Autophagosome Biogenesis

Autophagosome numbers can be measured by assessing phosphatidylethanolamine-conjugated ATG8/LC3 (LC3-II) levels

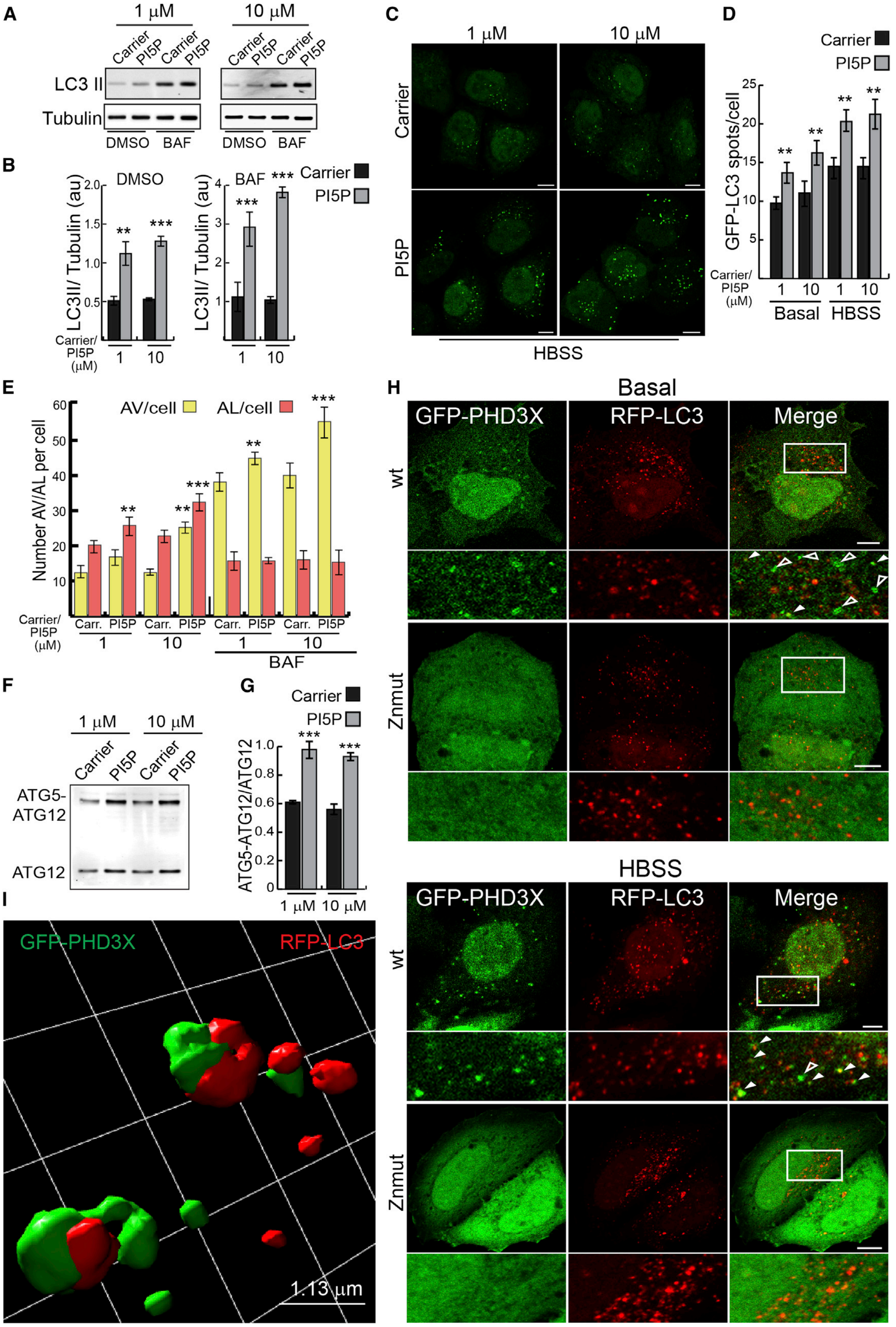

(legend on next page)

versus a loading control (e.g., tubulin), or by scoring the number of LC3-positive vesicles, since LC3-II is specifically targeted to autophagosomal membranes (Kabeya et al., 2000). In the same way that PI(3)P alone is sufficient to stimulate autophagy in mammalian cells (Petiot et al., 2000), we found that addition of exogenous PI(5)P di-C16 significantly increased LC3-II levels and LC3-positive autophagic vesicle numbers (Figures 1A–1D). While LC3-II levels correlate with autophagosome numbers, these can increase due to enhanced formation or decreased degradation (Rubinsztein et al., 2009). Consistent with a role in autophagosome synthesis, PI(5)P increased LC3-II levels, in an apparently dose-dependent manner, when we blocked LC3-II clearance by inhibiting autophagosome-lysosome fusion using Bafilomycin A1 (BAF) (Figures 1A and 1B and Figures S1A and S1B available online), and resulted in increased numbers of GFP-LC3 puncta (autophagosomes) in both nutrient-replete media and Hank's balanced salt solution (HBSS) (amino-acid/serum starvation and 1 g/l D-glucose, compared to 4.5 g/l in Dulbecco's modified Eagle's medium [DMEM]) (Figures 1C and 1D). The effects of PI(3)P (a positive control) were stronger at 0.1 μM compared to PI(5)P, while the effects of PI(5)P were more noticeable at 1 and 10 μM (Figures S1A and S1B). PI and PI(4)P did not obviously stimulate autophagy, and PI(4)P loading of cells treated with BAF suggested possible inhibition of autophagy (Figure S1C). PI(5)P loading in cells stably expressing mRFP-GFP tandem fluorescent-tagged LC3 (see Supplemental Experimental Procedures) increased the numbers of autophagosomes and autolysosomes (Figures 1E and S1D). Furthermore, addition of PI(5)P increased the conjugation of ATG5 with ATG12 (Figures 1F and 1G), a critical event in phagophore biogenesis that appears to be regulated by PI(3)P (Ravikumar et al., 2008).

Exogenous PI(5)P was delivered to autophagic structures, as we observed fluorescent BODIPY-labeled PI(5)P on RFP-LC3 puncta (Figure S1E). BODIPY-labeled PI(5)P loading of living cells increased the number of RFP-LC3 dots dose dependently (Figure S1E). One difficulty in studying the intracellular localization of endogenous PI(5)P is the uncertain specificity of PI(5)P bioprobes. While the plant homeodomain (PHD) of ING2 (Bua et al., 2013; Gozani et al., 2003) and the pleckstrin homology (PH) domain of Dok proteins (Guittard et al., 2009, 2010) show strong preference for PI(5)P and have been used for intracellular localization and manipulation of PI(5)P (Viaud et al., 2014a), we cannot exclude that they do not bind other lipids to some extent. With this caveat in mind, we detected GFP-tagged PHD3X (three tandem repeats of PHD of ING2) (Bua et al., 2013; Gozani et al., 2003; Guittard et al., 2010) localization at the nucleus and plasma membrane (as previously described) and on discrete puncta, 25% of which were RFP-LC3 positive (Figure 1H). When cells were starved, GFP-PHD3X-positive structures increased from 4–6 to >12 spots per cell, 50% of which colocalized with RFP-LC3 (Figure 1H). A PHD mutant defective in PI(5)P binding (PHD3X Znmut) (Bua et al., 2013) failed to localize on discrete structures (Figure 1H). Superresolution structured illumination microscopy (SR-SIM) confirmed the confocal localization of PHD3X on LC3-positive vesicles/autophagosomes (Figure 1I; Movie S1).

Overexpression of GFP-PHD3X, which sequesters intracellular PI(5)P, dramatically decreased the percentage of cells with more than 10 LC3 dots (both endogenous LC3 and RFP-tagged LC3 were assessed), compared to GFP-empty vector or GFP-PHD3X Znmut (Figures S1F–S1I), an effect mimicked by overexpression of GFP-PH-Dok-5, an alternative PI(5)P binding module (Guittard et al., 2010; Figures S1F and S1G). As we could not exclude that some of the effects of these probes may be due to binding to other lipids, we next manipulated intracellular PI(5)P levels by targeting enzymes relevant for its biogenesis and turnover.

PI(5)P Synthesized by PIKfyve Regulates Autophagosome Formation

Type III PtdInsP 5-kinase PIKfyve appears to regulate PI(5)P biosynthesis (Figure 2A; Sbrissa et al., 1999), as reduced PI(5)P levels are seen in *PIKfyve* hypomorph and heterozygous mice and in cells silenced by small interfering RNA (siRNA), overexpressing a dominant-negative mutant or treated with pharmacological inhibitor of the kinase (Ikonomov et al., 2011; Sbrissa et al., 2002, 2012; Zolov et al., 2012). Low doses (100 nM) of the PIKfyve inhibitor YM-201636 (Sbrissa et al., 2012) decreased LC3-II levels (Figures 2B, 2C, and S2A), the number of ATG16L1

Figure 1. PI(5)P Regulates Autophagosome Biogenesis

(A and B) Western blot analysis of LC3-II and tubulin levels and quantification of LC3-II/tubulin ratio in HeLa cells treated with carrier alone, or in combination with PI(5)P di-C16 at indicated concentrations for 1 hr, in the absence and presence of 400 nM BAF (treated in combination with lipids). Note that LC3-I is often very faint compared with LC3-II in HeLa cells under the protein extraction conditions we used (see Figure S1C). However, this was not a problem because one should relate LC3-II to tubulin (mean ± SEM).

(C and D) HeLa cells stably expressing GFP-LC3 were treated as in (A) and then left in complete media (basal) or starvation media (HBSS) for 2 hr, then fixed and analyzed on a Cellomics ArrayScan system. Quantification of numbers of GFP-LC3 vesicles per cell in the different conditions is shown in (D) (mean ± SEM).

(E) HeLa cells stably expressing GFP-mRFP-LC3 were treated as in (A) and analyzed on a Cellomics ArrayScan system. Quantification of numbers of autophagic vesicles (AV) or autolysosomes (AL) per cell in the different conditions is shown in the graph (mean ± SEM).

(F and G) Western blot analysis of free ATG12 and ATG5-ATG12 complex levels with anti-HA antibody in HeLa cells transfected with HA-ATG12 and ATG5 and loaded with exogenous lipids. We quantified the ratio of ATG5-ATG12 versus free ATG12 by direct infrared fluorescence detection on an Odyssey Infrared Imaging System to assess ATG5-ATG12 conjugation efficiency (mean ± SEM).

(H) HeLa cells transfected with GFP-PHD3X or GFP-PHD3X Znmut and RFP-LC3 for 16 hr were left in complete media (basal) or starvation media (HBSS) for 1 hr, then fixed and imaged on a confocal microscope. Empty arrowheads indicate PHD3X single-labeled structures, while filled arrowheads indicate PHD3X-LC3- and PHD3X-ATG16-positive structures. Bar, 10 μm.

(I) HeLa cells transfected with GFP-PHD3X and RFP-LC3 for 16 hr were left in HBSS for 1 hr, then fixed and imaged on Elyra superresolution microscope. Final visualization was performed in Volocity 6.3 software using isosurface rendering of selected cropped regions of the data sets. Note that this rendering means that vesicles positive for green and red do not look yellow but have green and red on the surface. Representative cropped regions from different cells are shown. Bar, 1.13 μm. See also Figure S1.

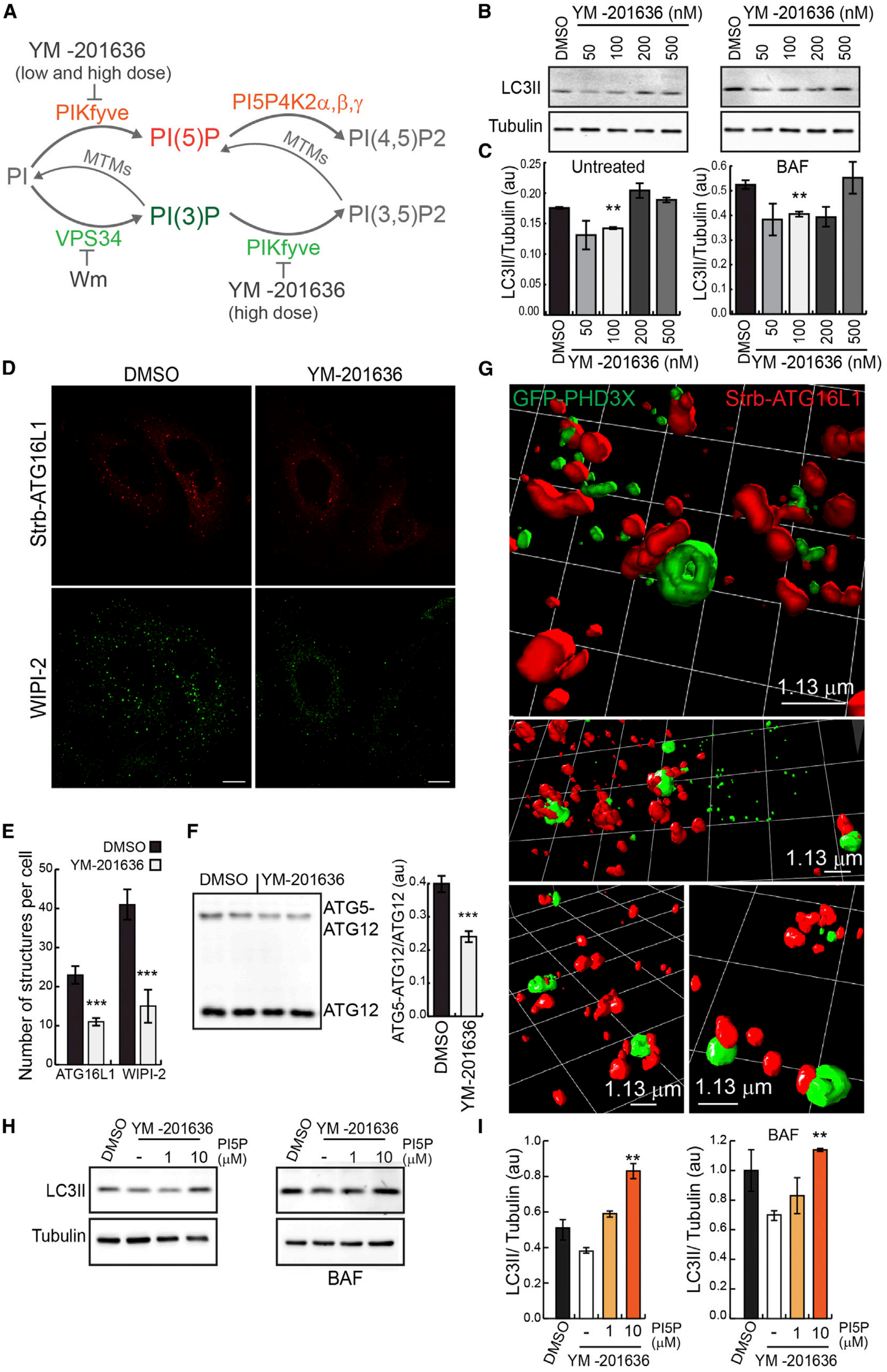

(legend on next page)

(autophagosome precursor) and WIPI-2 vesicles (Figures 2D and 2E), and ATG5-ATG12 conjugation (Figure 2F).

Autophagosomes may contain PI(5)P from early stages of their biogenesis, since GFP-PHD3X was associated with mStrawberry-ATG16L1 structures (which label phagophores or prephagophore structures) in both nutrient-replete and starved conditions (50% and 70% colocalization, respectively) (Figure S2B). A 3D analysis of PHD3X-labeled structures assessed by super-resolution structured illumination microscopy (SR-SIM) revealed PI(5)P on ATG16L1-positive vesicles in autophagy-stimulating conditions (amino-acid starvation, HBSS media) (Figure 2G; Movie S2). Low doses of YM-201636 (100 nM) or PIKfyve silencing selectively depleted GFP-PHD3X on ATG16L1 vesicles, consistent with a specific reduction of PI(5)P on autophagosome precursors (Figures S2B and S2C). To further validate the idea that PI(5)P is present on early autophagic membranes, we expressed the GFP-PHD3X probe, in cells where phagophores accumulated and autophagosome completion was impaired by the overexpression of proteolytic-activity-deficient mutant of ATG4B (ATG4BC74A), that prevents LC3 lipidation and inhibits autophagosome formation (Fujita et al., 2008). ATG4BC74A increased the numbers of ATG16L1 structures labeled with GFP-PHD3X, in both basal and starvation conditions (Figure S2D), with some forming large ring-shaped structures.

PIKfyve is also the primary kinase responsible for production of PI(3,5)P2, a key regulator of early-to-late endosome membrane trafficking (Shisheva, 2013; Figure 2A). Note that higher concentrations and longer treatment with YM-201636, which inhibits PI(3)P conversion to PI(3,5)P2, causing elevation of PI(3)P (de Lartigue et al., 2009; Zolov et al., 2012), do not reduce autophagosome numbers (Figures 2B, 2C, and S2A), possibly due to defects in the endosomal/lysosomal compartment that block autophagosome degradation (Figures 2A, S2A, and S2E). Consistent with this, a similar block in autophagosome degradation is seen with PIKfyve siRNA knockdown (Figure S2F). To parse out the effects of impaired autophagosome formation and degradation, we treated cells with BAF for 2 hr in the presence of HBSS (an autophagy stimulus), and we found less autophagosome formation in the PIKfyve knockdown cells (Figure S2G), consistent with reduced ATG5-ATG12 conjugation after PIKfyve knockdown (as seen with short-term low-dose YM-201636 treatment) (Figure S2H). Importantly, the addition of exogenous PI(5)P di-C16 to cells treated with low concentrations of YM-201636 (Figures 2H and 2I) or treated with PIKfyve siRNA (Figures S2G and S2H) significantly rescued LC3-II levels and ATG5-ATG12 conjugation, arguing that PI(5)P is the relevant signaling molecule in PIKfyve-dependent autophagosome biogenesis.

Several in vivo studies suggest that PIKfyve may indirectly control PI(5)P levels by producing PI(3,5)P2, which is then transformed into PI(5)P by 3-phosphatases of the myotubularin family (Figure 2A; Oppelt et al., 2013, 2014; Viaud et al., 2014b; Zolov et al., 2012). If this is the case, then the production of PI(5)P is likely localized to internal membranes where PI(3,5)P2 is located. We investigated the localization of PI(3,5)P2 using the tandem repeats of the N-terminal domain of mucolipin1 (ML1N*2), recently described as a reliable and specific probe for PI(3,5)P2 (Li et al., 2013). GFP-ML1N*2 associated with early autophagosomal structures labeled with mStrawberry-ATG16L1 under nutrient-starvation conditions (80% of ATG16 structures contain GFP-ML1N*2 during starvation versus 20% in complete media), and this association was sensitive to YM-201636 treatment (Figure S2I). Consistent with PI(3,5)P2 being a precursor of PI(5)P, we found that overexpression of myotubularin-related phosphatase 3 (MTMR3), one of the myotubularin PI 3-phosphatase enzymes previously implicated in PI(5)P production along with PIKfyve (Oppelt et al., 2013, 2014; Viaud et al., 2014b; Zolov et al., 2012), increased numbers of GFP-PHD3X structures, while its knockdown had the reverse effect (Figure S2J) during nutrient starvation (HBSS). Various myotubularin family members have been linked to autophagy, mainly in the regulation of PI3P metabolism (Taguchi-Atarashi et al., 2010; Vergne et al., 2009; Zou et al., 2012). Thus, myotubularins other than MTMR3 may also regulate PI(5)P levels during autophagy induction, like PI(3)P. PI(3,5)P2 localization during starvation resembles what we found for PI(5)P, and raises the possibility that PI(3,5)P2 may contribute as a precursor for the PI(5)P pool required for autophagy (Figure 2A). PI(5)P and its precursor PI(3,5)P2 localize on autophagosome precursors, and inhibition of their synthesis results in autophagy inhibition. Our data provide clear evidence that PI(5)P synthesis occurs during early stages of autophagosome formation and is modulated by nutrient state.

Kinases that Convert PI(5)P to PI(4,5)P2 Regulate Autophagy

The major route for PI(5)P removal is attributed to type II PI5PK kinases (phosphatidylinositol 5-phosphate 4-kinases, PI5P4K2s) (Figure 2A; Clarke et al., 2010; Shisheva, 2013; Viaud et al., 2014a). Mammalian genomes contain three genes, PI5P4K2A,

Figure 2. PI(5)P Synthesized by PIKfyve Regulates Autophagosome Formation

(A) Schematic representation of pathways for PI(5)P and PI(3)P synthesis/turnover and drugs targeting enzymes involved in these pathways.

(B and C) Western blot analysis of LC3-II and tubulin levels and quantification of LC3-II/tubulin ratio in HeLa cells treated with DMSO or increasing concentrations of YM-201636 for 2 hr in the presence or absence of BAF (mean ± SEM).

(D and E) HeLa cells transiently transfected with Strawberry-ATG16L1 were treated with YM-201636 (100 nM, 2 hr) in HBSS, then fixed and stained for endogenous WIPI-2. Bar, 10 μm. (E) Quantification of ATG16L1 and WIPI2 structures per cell, n = 20 cells (mean ± SEM; n = 3 independent experiments; ***$p < 0.001$, t test).

(F) Western blot analysis of free ATG12 and ATG5-ATG12 complex levels with anti-HA antibody in HeLa cells transfected with HA-ATG12 and ATG5 and treated with YM-201636 (100 nM, 2 hr) (mean ± SEM).

(G) HeLa cells transfected with GFP-PHD3X and Strawberry-ATG16L1 for 16 hr were left in HBSS for 1 hr, then fixed and imaged on Elyra superresolution microscope. Final visualization was performed as previously described. Bar, 1.13 μm.

(H and I) Western blot analysis of LC3-II and tubulin levels and quantification of LC3-II/tubulin ratio in cells incubated with YM-201636 (100 nM, 2 hr) and loaded with exogenous PI(5)P for the last 1 hr in the presence or absence of BAF (mean ± SEM). See also Figure S2.

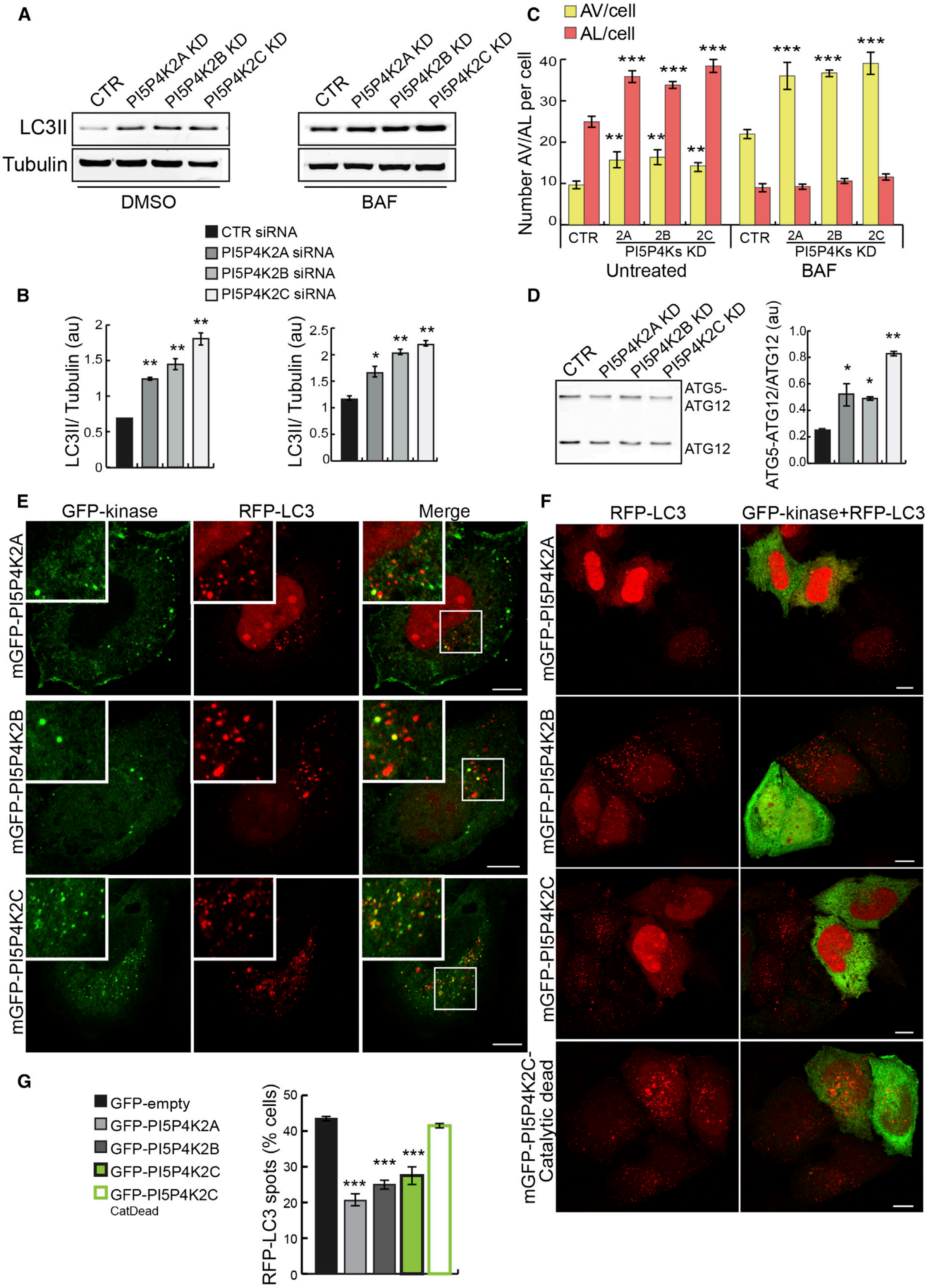

(legend on next page)

B, and C, encoding three type II PI(5)P 4-kinase isoforms, alpha (PI5P4K2α), beta (PI5P4K2β), and gamma (PI5P4K2γ), respectively. Silencing of the three PI5P4K2s, which increase cellular PI(5)P levels (Sarkes and Rameh, 2010; Wilcox and Hinchliffe, 2008), increased autophagosome formation (increased levels of LC3-II in the presence of BAF) (Figures 3A, 3B, and S3A–S3E), autophagosome and autolysosome numbers (in cells stably expressing mRFP-GFP tandem fluorescent-tagged LC3) (Figures 3C and S3F), and ATG5-ATG12 conjugation (Figure 3D). PI5P4K2A, B, and C silencing resulted in more obvious localization of GFP-PHD3X on autophagosome precursors and mature autophagosomes (ATG16L1 and RFP-LC3-positive structures) (Figure S3G). Consistent with the distribution of PI(5)P (Figure 1H), PI5P4K2α, β, and γ localized to different extents on autophagosomes (RFP-LC3 structures, Figure 3E), with PI5P4K2γ showing the most prominent association with autophagosomal structures. The different PI5P4K2 isoforms appeared to have different efficacies. While this may be due to different knockdown efficiencies, we noted that PI5P4K2C, whose knockdown had the strongest effect, was also most obviously associated with autophagosomes. Overexpression of active GFP-tagged PI5P4K2α, β, and γ, which attenuate signaling from PI(5)P (by consuming PI(5)P to produce PI(4,5)P2), impaired autophagy (Figures 3F and 3G), while a GFP-tagged PI5P4K2γ-inactive mutant did not (Figures 3F and 3G). These data indicate a function for PI(5)P in the induction of autophagy.

PI5P4K2s Alter the Levels of Autophagy Substrates

PI(5)P loading significantly increased LC3-II levels (in the absence and presence of BAF) in both human neuroblastoma (SKNSH) and mouse embryonic fibroblasts (MEFs) (Figures 4A and 4B). We investigated the effects of PI5P4K2s knockdown and overexpression on the levels of diverse autophagic substrates, including the ubiquitin-binding adaptor protein p62 and a mutant form of huntingtin associated with Huntington's disease (EGFP-httQ74) (Ravikumar et al., 2002). PI5P4K2s knockdown decreased the levels of p62 (Figures S3A–S3C) and the numbers of cells with mutant huntingtin aggregates (Figures 4C and 4D), which correlate with mutant protein levels (Narain et al., 1999), like other autophagy inducers (Williams et al., 2008), while PI5P4K2C overexpression had the opposite effect (Figures 4C–4E). While PI5P4K2C silencing decreased the proportion of cells with EGFP-httQ74 aggregates and the opposite was seen with PI5P4K2C overexpression, these effects were only seen in wild-type (WT, $Atg5^{+/+}$) MEFs, and not in autophagy-deficient cells ($Atg5^{-/-}$), suggesting these PI5P4K2C effects are autophagy dependent (Figures 4F, 4G, S4A, and S4B).

PI(5)P Sustains Autophagy in Cells Depleted of PI(3)P

We tested whether PI(5)P could sustain autophagy in cells depleted of PI(3)P with the VPS34 inhibitor Wm. We validated the specificity of Wm for VPS34 activity during autophagy induction by treating VPS34-null MEFs (Figure S5A) and HeLa or GFP-LC3 HeLa stable cell lines with Wm for 2 hr in HBSS (Figures S5B and S5C). Consistent with previous studies, Wm inhibited ATG5-ATG12 conjugation, LC3 lipidation, and prevented autophagosome formation (Blommaart et al., 1997; Ravikumar et al., 2008; Figures S5A–S5D; Figures 5A–5D). VPS34-null MEFS are insensitive to Wm compared to their WT counterparts (Figure S5A), and autophagy inhibition caused by Wm in HBSS-treated cells was rescued by PI(3)P supplementation (Figures S5B and S5C). VPS34-null cells did not have obviously decreased autophagy in HBSS (Figure S5A) like Wm-treated cells, suggesting that long-term VPS34 depletion may be compensated for by alternative pathways or by additional defects in autophagosome degradation (Devereaux et al., 2013). PI(3)P also regulates proteins crucial for sorting to vacuoles or lysosomes (Schink et al., 2013), and long-term VPS34 inhibition can impair endocytic trafficking (Petiot et al., 2003; Siddhanta et al., 1998) and cause vacuolation of late endosomal compartments (Futter et al., 2001) and mistrafficking of cathepsin D from late endosomes to lysosomes (Row et al., 2001).

To test whether PI(5)P was able to sustain autophagy in cells depleted of PI(3)P, we used acute Wm treatment and loaded cells with exogenous PI(5)P di-C16. Under these conditions, the requirement for the generation of PI(3)P appeared to be abrogated, as we detected LC3 lipidation (Figures 5A and 5B), increased LC3 vesicles under starvation (Figures 5C and S5C), increased double-membraned autophagosomes by electron microscopy (Figure S5D), and ATG5-ATG12 conjugation (Figure 5D), even in the presence of Wm. Similarly, increasing endogenous PI(5)P levels by PI5P4K2s knockdown resulted in an increase in LC3-II levels (Figures 5E and 5F), ATG5-ATG12 conjugation (Figures 5G and 5H), and numbers of LC3 vesicles (Figures 5I and S5E) that were resistant to Wm.

As a genetic alternative to Wm, we performed a short-term siRNA knockdown of VPS34, which does dramatically impact autophagy in HBSS (in contrast to longer-term knockdowns or Cre-mediated gene excision in the MEFs) (Figure S5A). In such VPS34-siRNA-treated cells, PI(5)P was able to rescue autophagy and ATG5-ATG12 conjugation. (Figure S5F).

We also manipulated PI(5)P and PI(3)P levels by overexpressing MTMR3, which would decrease PI(3)P levels but increase PI(5)P levels (Figures 2A and S5G). MTMR3 overexpression (but not MTMR3C413S catalytic mutant overexpression) in

Figure 3. PI5P4K2s Localize on Autophagosomes and Regulate Autophagy

(A and B) Western blot analysis of LC3-II and tubulin levels and quantification of LC3-II/tubulin ratio in HeLa cells transfected for 5 days with two rounds of control, PI5P4K2A, 2B, or 2C siRNA either left untreated or treated with BAF (200 nM, 16 hr) (mean ± SEM).

(C) HeLa cells stably expressing GFP-mRFP-LC3 treated as in (A) were analyzed on a Cellomics ArrayScan system as previously described (mean ± SEM).

(D) Western blot analysis of free ATG12 and ATG5-ATG12 complex levels with anti-HA antibody in HeLa cells treated with control, PI5P4K2A, 2B, and 2C siRNA and transfected with HA-ATG12 and ATG5 for the last 16 hr (mean ± SEM).

(E and F) HeLa cells transfected with GFP-PI5P4K2A, 2B, 2C, or catalytic-dead PI5P4K2C along with RFP-LC3 for 16 hr (E) or 30 hr (F) were fixed and imaged on a confocal microscope. Bar, 10 μm.

(G) Quantification of cells (percentage of total) showing more than ten autophagic vesicles (RFP-LC3 vesicles) in the different conditions from (F) is shown in the graph; n = 200 cells (mean ± SEM). See also Figure S3.

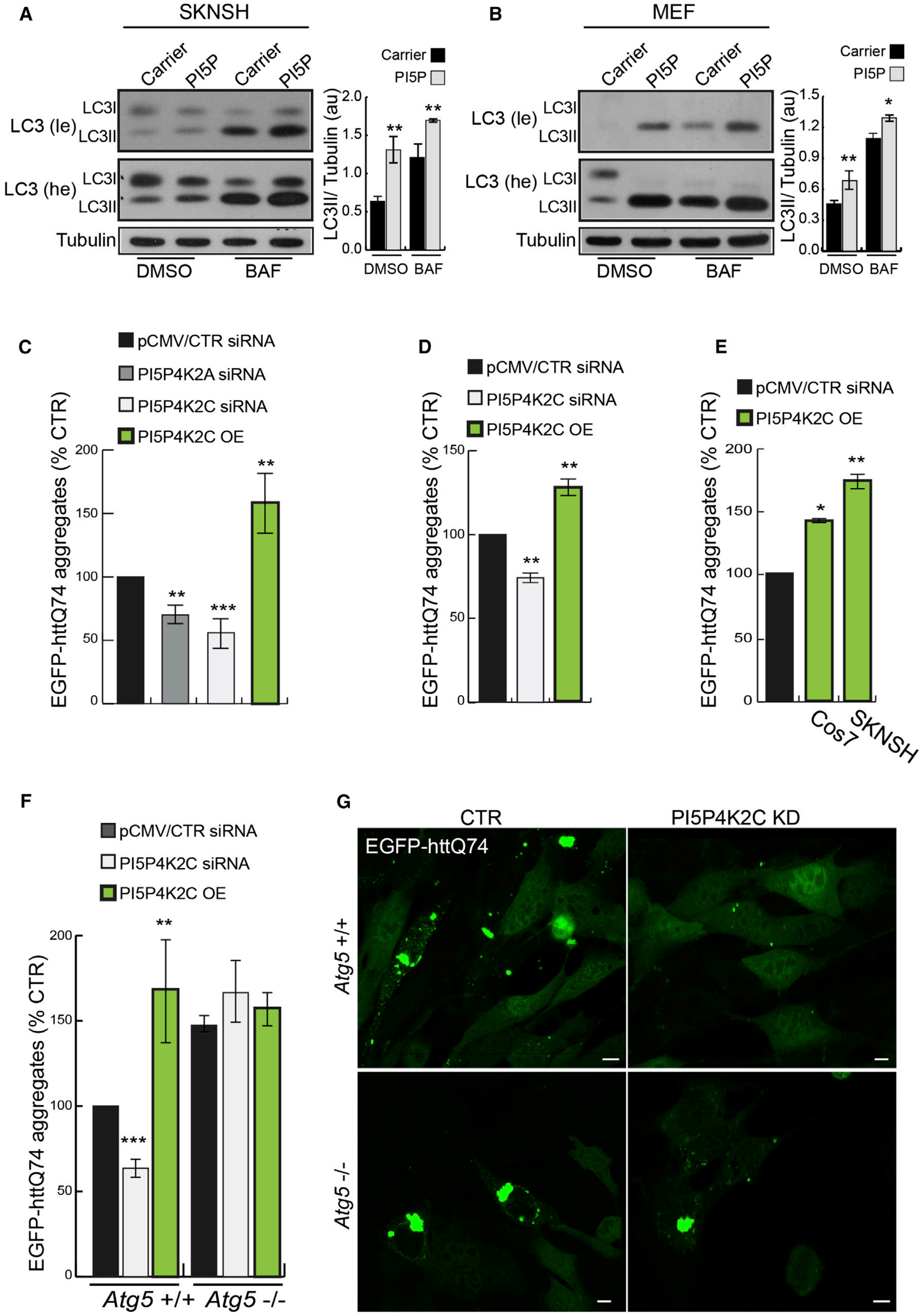

(legend on next page)

GFP-LC3 HeLa cells incubated in HBSS impaired autophagosome formation (BAF for 4 h), as previously reported (Taguchi-Atarashi et al., 2010; Figures 5J and 5K). This effect is likely due to the effects of MTMR3 on PI(3)P catabolism, which would be expected to blunt the HBSS-induced autophagy-stimulating levels of this lipid. To measure the effect of MTMR3 overexpression in a context where the role of PI(3)P can be excluded (Figure 2A), to allow us to largely focus our attention on the ability of this enzyme to generate PI(5)P, we treated cells with BAF and Wm for 4 hr in the presence of HBSS, and we found that overexpression of WT, but not of catalytic dead MTMR3 (Figures 5J and 5K), could rescue LC3 vesicle numbers in Wm-treated cells. Conversely, increasing PI(3)P (by loading of exogenous PI(3)P di-C16) restored LC3 dots in cells depleted of PI(5)P by overexpression of active PI5P4K2α, β, and γ (Figures 5L and 5M). Thus, our results point toward a common mechanism of action for PI(3)P and PI(5)P during autophagy.

PI(5)P and PI(3)P Share Common Effectors during Autophagosome Formation

Since PI(5)P can rescue autophagy in PI(3)P-depleted cells, we tested if PI(5)P regulates autophagosome formation similarly to PI(3)P, through the recruitment of the WIPI2 and DFCP1 proteins. GFP-WIPI2 and DFCP1 puncta, which disappeared in starved cells treated with Wm, remained when such Wm-treated cells were preloaded with PI(5)P (Figures 6A and S6A), and the membrane association of GFP-WIPI2 and GFP-DFCP1 was dramatically impaired by Wm, but was Wm-insensitive in PI5P4K2 knockdown cells (Figures 6B–6D and S6B). Thus, WIPI2 and DFCP1 membrane binding can be preserved by PI(5)P in Wm-treated cells.

We examined whether WIPI2 proteins bind to PI(5)P using extracts from cells expressing GFP-tagged WIPI2B and WIPI2D, which we then precipitated with beads coated with different PIs (Figures 6E and S6C). WIPI2 proteins were associated with PI(5)P beads, but not with PI or uncoated beads (Figures 6E and S6C). This association seemed to be specific because the PI(5)P binding was inhibited when cell extracts were preincubated with PI(5)P-containing but not PS-containing liposomes (Figure 6F). Similar effects also were observed for the binding of WIPI2B to PI(3)P beads and for the competition of PI(3)P-containing liposomes with PI(3)P beads (Figures 6E and 6G). WIPI2B protein pull-down by the PI(3)P beads was strongly affected by preincubation with PI(5)P-containing liposomes (Figure 6H). Likewise, WIPI2B protein pull-down by the PI(5)P beads was strongly affected by preincubation with PI(3)P-containing liposomes (Figure 6H). Furthermore, a WIPI2 mutant that was predicted not to bind to PI(3)P (Dooley et al., 2014) had similarly reduced binding to PI(5)P (Figure 6I). Thus, PI(5)P binds a PI(3)P effector involved in autophagosome biogenesis; we tested if PI(5)P was required for PI(3)P-independent autophagy.

Autophagy Activation following Glucose Starvation Is Dependent upon PI(5)P, but Not PI(3)P

Since we found that PI(5)P can sustain autophagy in Wm-treated cells, we considered that PI(5)P may regulate autophagy pathways where PI(3)P is dispensable, such as glucose-starvation-induced autophagy (McAlpine et al., 2013), a hypothesis strengthened by our observation that HBSS or glucose starvation increase the phosphorylation of PIKfyve at residues where this enhances its catalytic activity (Er et al., 2013; Hill et al., 2010; Liu et al., 2013; Figure S7A). Glucose deprivation in HeLa cells stably expressing GFP-LC3 increased the number of autophagosomes in DMSO- and Wm-treated cells, but not when PIKfyve was inhibited by YM-201636 treatment (Figures 7A and 7B). Conversely, both Wm and YM-201636 strongly affected but did not completely ablate autophagosome formation under amino-acid/serum starvation (HBSS, Figures 7A and 7B). We confirmed that YM-201636 reduced LC3-II levels in both glucose-starved MEFs and SKNSH cells, and that this phenomenon was not seen with Wm (Figure 7C). Consistent with a specific requirement for PI(5)P for autophagy induction during glucose withdrawal, we observed a significant YM-201636-sensitive increase in the number of ATG16-positive autophagosomes containing PI(5)P in glucose-depleted cells (Figure 7D). When PI(5)P was sequestered by GFP-PHD3X overexpression or removed by overexpression of active PI5P4K2α, β, and γ, the appearance of LC3 dots following glucose starvation was abolished (Figures 7E, 7F, and S7B). Consistent with the different effects of Wm treatment under nutrient starvation, we did not detect an increased number of PI(3)P-containing autophagosomes (using the GFP-FYVE2X probe or anti-PI(3)P antibodies) in cells depleted of glucose compared to HBSS media (Figures S7C and S7D).

Supporting the idea that PI(3,5)P2 contributes to the generation of PI(5)P during nutrient starvation, we observed increased colocalization of GFP-ML1N*2 with RFP-LC3 under glucose and amino-acid/serum starvation (Figure S7E). Indeed, MTMR3 knockdown significantly reduced PI(5)P on autophagosomes (Figure 7G), inhibited autophagosome formation in glucose-starved cells (where PI(3)P is dispensable), and this effect could be reversed by adding back PI(5)P (Figures S7F and S7G). Collectively, our results argue that PI(5)P generated by PIKfyve

Figure 4. PI5P4K2A and PI5P4K2C Regulate Accumulation of Mutant Huntingtin Exon 1

(A and B) Western blot analysis of LC3-II and tubulin levels and quantification of LC3-II/tubulin ratio in SKNSH and MEFs treated for 2 hr with carrier alone or in combination with PI(5)P di-C16 (10 μM) in the absence and presence of BAF (mean ± SEM).

(C) HeLa cells treated with PI5P4K2A or PI5P4K2C siRNA for 5 days or with pCMV-PI5P4K2C overexpression construct for 48 hr were transfected with EGFP-httQ74 for the last 48 hr. Percentage of cells with EGFP-positive aggregates is shown in the graph; n = 500 cells (mean ± SEM). Cells expressing control vector typically have 25% of aggregates and we set the control at 100% to enable comparison and statistics from independent experiments.

(D and E) Percentage of cells with EGFP-positive aggregates was scored in HEK293 cells treated with PI5P4K2C siRNA for 5 days and cotransfected with EGFP-httQ74 for the last 48 hr (D), or in COS7 and SKNSH cells (E), transfected with PI5P4K2C plasmid together with EGFP-httQ74 for the last 48 hr.

(F and G) WT (*Atg5*+/+) or autophagy-deficient cells lacking the key autophagy gene *Atg5* (*Atg5*−/−) MEFs were treated with PI5P4K2C siRNA (5 days) or PI5P4K2C plasmid (48 hr) together with EGFP-httQ74 for 48 hr. The percentages of transfected cells with EGFP-httQ74 aggregates were assessed and shown in the graph; n = 500 cells (mean ± SEM). Representative confocal images are shown in (G). See also Figures S3 and S4.

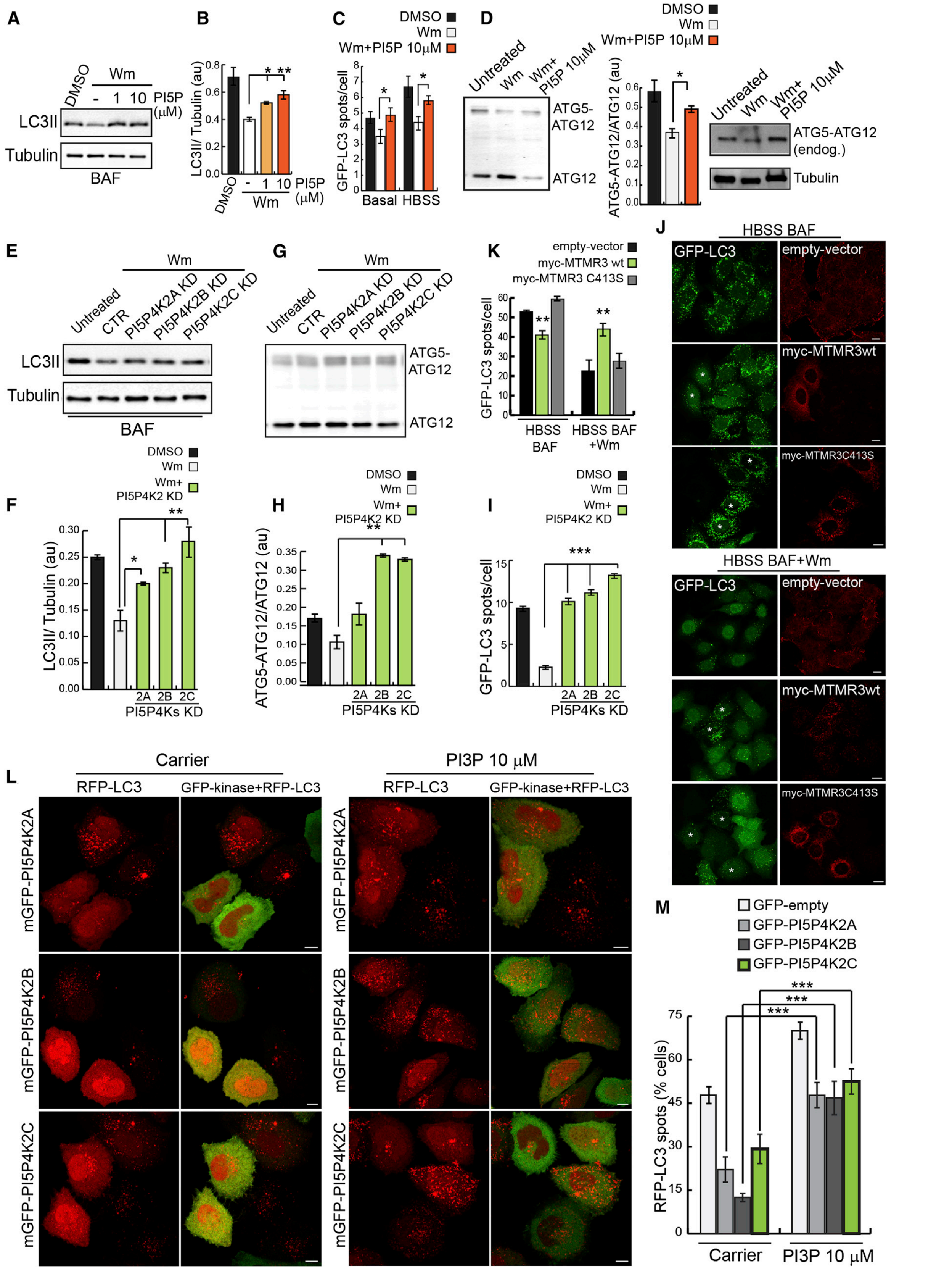

(legend on next page)

and MTMR3 is the relevant lipid species for autophagosome generation during glucose starvation. Resveratrol induces autophagy in noncanonical manner, independent of Beclin1 and partially resistant to Wm (Mauthe et al., 2011). The autophagy stimulation of resveratrol was impaired upon PI(5)P depletion due to overexpression of PI5P4K2α, β, and γ (but not the catalytic-dead mutant of PI5P4K2γ or GFP-empty vector) (Figure S7H).

When we labeled cells in basal, HBSS, or glucose starvation for PI(3)P (with an antibody) and for PI(5)P (with GFP-PHD3X), it was notable that the glucose-starved cells had fewer PI(3)P- and more PI(5)P-containing ATG16L1 structures, some of which appeared devoid of PI(3)P (Figure 7H), consistent with a dominant role for PI(5)P during glucose starvation. Our findings suggest that both PI(5)P and PI(3)P are important regulators of autophagy induced by HBSS. However, PI(5)P synthesis, but not PI(3)P synthesis, is required for autophagy induction by glucose withdrawal.

DISCUSSION

The present work identifies a role for PI(5)P as a regulator of autophagosome biogenesis. PI(5)P synthesis is required for autophagosome formation and this effect is similar to PI(3)P, as both lipids regulate recruitment of the WIPI2 and DFCP1 proteins and ATG5-ATG12 conjugation. PI(5)P and kinases acting on PI(5)P (particularly PI5P4K2γ) are associated with autophagosomes and autophagosome precursors, suggesting that local alterations in the levels of this lipid are important for regulating autophagy. While we cannot exclude that alterations in PI(5)P may impact autophagosome biogenesis through its metabolites, the observations that PI(5)P binds to WIPI2, impacts PI(3)P-related phenotypes like ATG5-ATG12 conjugation, and can rescue autophagy in Wm-treated HBSS-starved cells strongly argue that it can serve as an alternative to PI(3)P. It is worth bearing in mind that PI(5)P's presence in cells is ~100 times lower than those of the most abundant Pis, PI(4,5)P2 and PI(4)P (Sarkes and Rameh, 2010). The major source of PI(4,5)P2, which we previously have shown to be a positive regulator of autophagy (Moreau et al., 2012), is PI(4)P in resting cells (Rameh et al., 1997; Whiteford et al., 1997). Thus, reductions of PI(5)P levels hardly affect PI(4,5)P2.

The ability of PI(5)P to regulate autophagy in cells depleted of PI(3)P suggests that PI(5)P may account for the previously enigmatic concept of PI(3)P-independent, noncanonical autophagy. While we cannot exclude that a small amount of cellular PI(3)P may be generated via VPS34-independent routes, as suggested recently (Devereaux et al., 2013; McAlpine et al., 2013; Zhou et al., 2010), we consciously used acute depletion of PI(3)P using Wm to reduce the likelihood of such minor pathways having overt effects (compared to using genetic approaches, which would be necessarily more chronic and would allow redundant pathways to become more apparent). Indeed, Wm completely ablated the presence of WIPI2 and DFCP1 vesicles in our experiments. Since these effects and the concomitant ATG5-ATG12 conjugation and autophagosome formation were completely rescued by elevating PI(5)P in Wm-treated cells, we suggest that PI(5)P can account for the previously mysterious phenomenon of PI(3)P-independent autophagy. While this would be compatible with the similar binding of PI(3)P and PI(5)P to WIPI proteins (Baskaran et al., 2012; Jeffries et al., 2004), it is possible that the two lipids may have some nonredundant properties in the autophagy context. Indeed, this appeared to be the case, as we found PI(5)P (and its precursor PI(3,5)P2) localized on nascent and mature autophagosomes during glucose starvation, and this is highly dependent on the activity of the type III PtdInsP 5-kinase PIKfyve. Furthermore, glucose-starvation-induced autophagy appeared to be dependent on PI(5)P and not PI(3)P. The Wm resistance of glucose-starvation-induced autophagy was seen in HeLa cells, SKNSH cells, and MEFs. While we do not know why this is the case, given that one would expect VPS34 to be activated by upstream kinases under these conditions, one may speculate that it is not sufficient simply to activate the enzyme, but one also needs the enzyme to be in the right place, and perhaps the mechanism governing the correct localization of VPS34 to autophagosome precursors may be inefficient in glucose-starved cells.

Figure 5. PI(5)P Triggers Autophagy in the Absence of VPS34 Activation

(A and B) Western blot analysis of LC3-II and tubulin levels and quantification of LC3-II/tubulin ratio in HeLa cells pretreated with Wm (200 nM, 2 hr), then loaded with 10 μM PI(5)P for 1 hr (in the presence of Wm and BAF) (mean ± SEM).

(C) Quantification of numbers of GFP-LC3 vesicles in HeLa cells stably expressing GFP-LC3 treated as in (A) and then shifted to starvation media (HBSS) or complete media (Basal) for 2 hr (in the presence of Wm) (mean ± SEM).

(D) HeLa cells transfected with HA-ATG12 and ATG5 treated with 200 nM Wm as in (A) and subjected to western blot analysis with anti-HA antibody to detect free ATG12 and the ATG5-ATG12 complex (mean ± SEM). ATG5-ATG12 conjugation was checked for endogenous proteins in the same conditions using an anti-ATG12 antibody (bottom).

(E and F) Western blot analysis of LC3-II and tubulin levels and quantification of LC3-II/tubulin ratio in HeLa cells transfected with control, PI5P4K2A, 2B, and 2C siRNA and treated with Wm (200 nM, 2 hr in the presence of BAF) (mean ± SEM).

(G and H) Western blot analysis of free ATG12 and ATG5-ATG12 complex levels with anti-HA antibody in HeLa cells treated with control, PI5P4K2A, 2B, and 2C siRNA, transfected with HA-ATG12 and ATG5 for last 16 hr and treated with Wm for 2 hr (mean ± SEM).

(I) HeLa cells stably expressing GFP-LC3 treated with control, PI5P4K2A, 2B, and 2C siRNA, were pretreated with Wm for 2 hr in complete medium and then shifted in HBSS media for 2 hr (in the presence of Wm) (mean ± SEM).

(J and K) HeLa cells stably expressing GFP-LC3 were transfected for 30 hr with myc-tagged empty vector, myc-MTMR3WT, and myc-MTMR3C413S, incubated for 4 hr in HBSS in the presence of BAF and in the presence or absence of Wm. Cells were fixed, stained with anti-myc antibodies, and imaged by confocal microscope. Asterisks indicate transfected cells. Quantification of numbers of GFP-LC3 vesicles per cell is shown in (K) (mean ± SEM).

(L and M) HeLa cells transfected with GFP-PI5P4K2A, 2B, 2C, and RFP-LC3 for 30 hr were loaded with 10 μM PI(3)P for 1 hr in complete medium, and then fixed and imaged on confocal microscope. Bar, 10 μm. Quantification of cells (percentage of total) showing more than 10 RFP-LC3 vesicles in the different conditions from (J) is shown in (M); n = 200 cells (mean ± SEM). See also Figure S5.

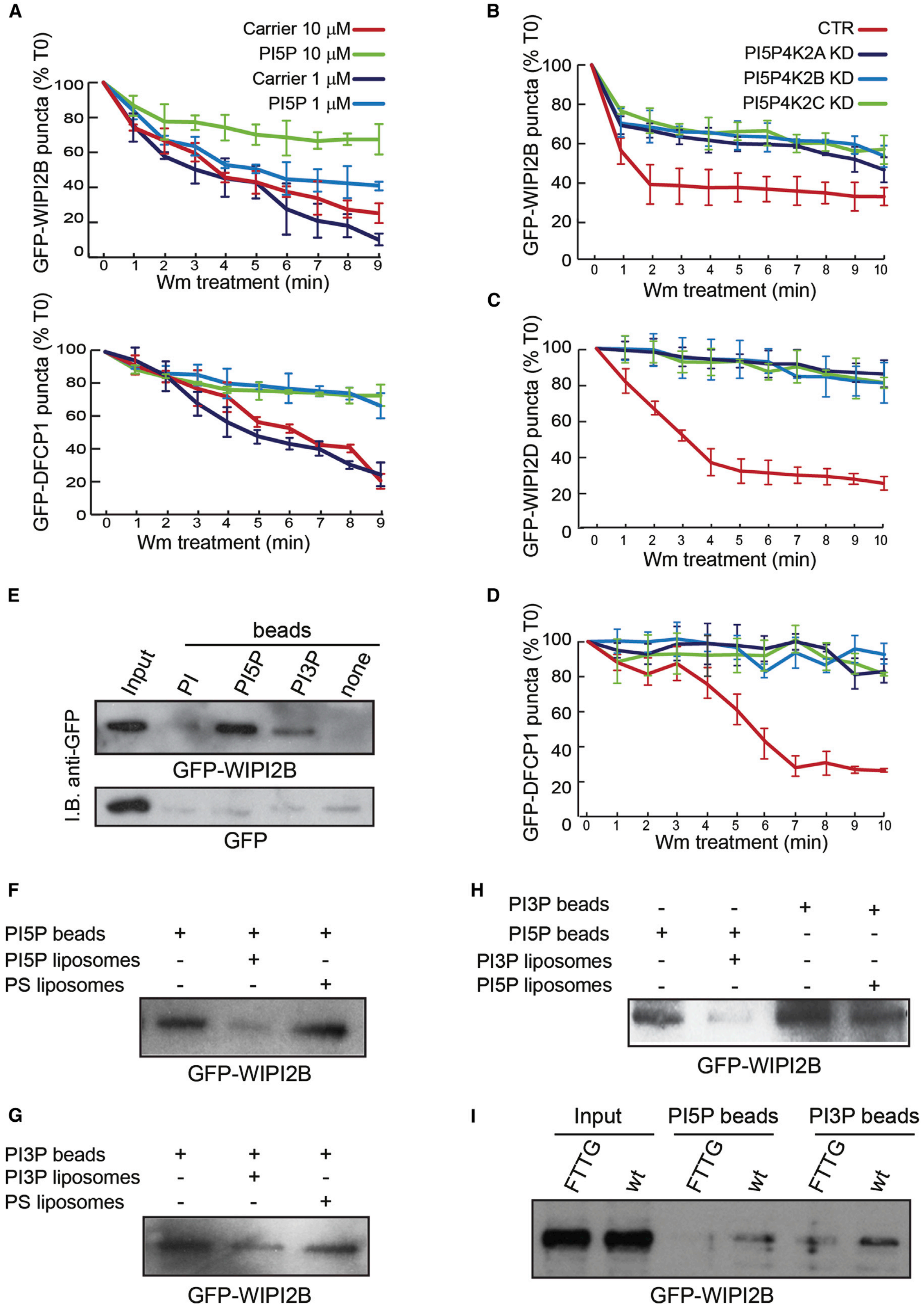

(legend on next page)

Our observations may have therapeutic potential, because PIKfyve and PI5P4K2 enzymes are drug-treatable targets (Davis et al., 2013; Jefferies et al., 2008), and our data indicate that suppression of PI5P4K2's activity increased the clearance of disease-associated autophagic substrates. Autophagy induction via other signaling effectors has benefits in a wide range of neurodegenerative diseases caused by aggregate-prone intracytoplasmic proteins, like Huntington's and Parkinson's diseases (Harris and Rubinsztein, 2011). Thus, PI5P4K2's inhibition may provide a tractable therapeutic target for neurodegenerative diseases.

EXPERIMENTAL PROCEDURES

Addition of Exogenous Lipids to Cells

Unlabeled PI, PI(5)P, PI(3)P, and PI (4)P di-C16; BODIPY-labeled PI(5)P and PI(3)P di-C6; and carrier (Echelon) were reconstituted in H2O:tert-BuOH (9:1) solution. After 1 min bath sonication, carrier and lipids were combined at a 1:1 ratio for 10 min at room temperature. The mixture of lipids and carrier was diluted in media and used for 1–2 hr incubations on cells. The final concentrations used were 0.1-1-10 μM. For the negative control; DMEM was combined with carrier only and added to the cells.

Lipid Beads Pull-Down Assay

HeLa cells (stably expressing GFP alone or GFP-WIPI2B or transiently expressing WIPI2D) were suspended in lipid-binding buffer (20 mM Tris-HCl, 150 mM NaCl, and 1 mM EDTA [pH 7.5]). The cells were passed ten times through a G25 needle and sonicated on ice. After insoluble debris was removed by high-speed centrifugation at 13,000 × *g* for 1 hr at 4°C, a 50 μl slurry of PI, PI(3)P, PI(5)P, or unbound beads (Echelon Bioscience) was added to the tube and incubated for 4 hr at 4°C under rotary agitation. The beads were washed five times with lipid-wash buffer (10 mM HEPES [pH 7.4], 150 mM NaCl, 0.25% NP40), and the bound proteins were subjected to immunoblotting. For competitive inhibition with liposomes, cell extracts were incubated on ice with 50 μM PS/PI(5)P, PS/PI(3)P liposomes for 3 hr before the addition of PI(5)P or PI(3)P beads. Preincubation of the cell lysates with liposomes containing only PS was used as a control with the same negative net charge.

SR-SIM

Samples were processed for conventional fluorescence microscopy and mounted on high-precision size 1.5 coverslips (Carl Zeiss). Coverslips were mounted with ProLong Gold antifade medium (P36934, Life Technologies), which was left to cure for 3 days at room temperature to produce samples with a consistent refractive index. SR-SIM was performed using an Elyra PS1 instrument (Carl Zeiss). Samples were examined on the microscope using a 63× 1.4NA plan-apo Carl Zeiss objective lens and Immersol 518F (23°C) immersion oil. Image acquisition was carried out using ZEN 2012 Elyra edition software, in which data sets were collected with five grating phases, five rotations, and sufficient z positions spaced 110 nm apart to form an approximately 2 μm deep volume of raw SR-SIM data. Optimal grating frequencies were selected for each wavelength used. Structured illumination postprocessing was performed in ZEN using parameters determined by automated analysis of the data sets. Reconstructed images were then corrected for spherical and chromatic aberrations using channel alignment information, which was created using a 3D array of multispectral beads previously imaged with the same instrument settings. The average final image resolution was calculated to be 110 nm in x and y dimensions and 240 nm in the z dimension, which represents a 2-fold lateral and axial improvement in resolution compared to conventional microscopy. Final visualization and video production was performed in Volocity 6.3 software using isosurface rendering of selected cropped regions of the data sets.

Statistics

Protein levels were expressed as arbitrary units and numbers of vesicles or aggregate formation were expressed as percentages from three independent experiments carried out in triplicate, and the error bars denote SEM. Significance levels for comparisons between two groups were determined with two-tailed Student's t test (Microsoft Excel); *$p < 0.05$; **$p < 0.01$; ***$p < 0.001$.

SUPPLEMENTAL INFORMATION

Supplemental Information includes Supplemental Experimental Procedures, seven figures, one table, and two movies and can be found with this article online at http://dx.doi.org/10.1016/j.molcel.2014.12.007.

ACKNOWLEDGMENTS

We are grateful for funding from a Wellcome Trust Principal Research Fellowship (095317/Z/11/Z to D.C.R.), a Wellcome Trust Strategic Award (100140/Z/12/Z), the NIHR Biomedical Research Centre in Dementia at Addenbrooke's Hospital, an MRC Confidence in Concepts grant (D.C.R.), and a FEBS Long-Term Fellowship (A.A.). We thank Maria Jimenez-Sanchez and Gautam Runwal for technical assistance (Cre recombinase lentivirus production and MEF infection, GFP-WIPI2B, and empty GFP stable HeLa cell lines), B. Payrastre (Inserm/UPS UMR 1048-I2MC, Tolouse) for providing GFP-PHD3X and GFP-PHD3X Znmut plasmids, J.A. Nunes (Centre de Recherche en Cancerologie de Marseille) for GFP-PH-Dok5 plasmid, T. Proikas-Cezanne (Eberhard Karls University Tübingen) for GFP-WIPI2B and GFP-WIPI2D plasmids, N.T. Ktistakis (Babraham Institute, Cambridge, UK) for GFP-DFCP1 plasmid, N. Mizushima (The Tokyo Metropolitan Institute of Medical Science Japan) for ATG12-HA and ATG5 plasmids, H. Stenmark (University of Oslo, Norway) for GFP-FYVE plasmid, and H. Xu (University of Michigan, USA) for GFP-ML1N*2.

Received: June 13, 2014
Revised: October 22, 2014
Accepted: November 25, 2014
Published: January 8, 2015

Figure 6. PI(5)P Recruits Proteins Required for the Initiation of Autophagosome Formation

(A) HeLa cells transfected with GFP-WIPI2B or GFP-DFCP1 preloaded with indicated concentrations of PI(5)P for 1 hr, starved in HBSS for 1 hr, and then incubated with Wm in HBSS were tracked by time-lapse microscopy for 10 min after the addition of Wm. Quantification of WIPI2B or DFCP1 vesicles (percentage of those at the starting time [T0]) during the treatments are shown in the graphs.

(B–D) HeLa cells treated with control, PI5P4K2A, 2B, and 2C siRNA were transfected with GFP-WIPI2B (B), GFP-WIPI2D (C), or GFP-DFCP1 (D); starved in HBSS (1 hr); and then incubated with Wm in HBSS. WIPI2 or DFCP1 structures were tracked and quantified as in (A).

(E) Lysates from HeLa cells stably expressing GFP-WIPI2B were incubated with agarose beads coated with PI, PI(5)P, and PI(3)P, eluted with SDS-PAGE sample buffer, and recovered proteins were assessed by western blotting using antibodies against GFP. Uncoated beads and lysates from HeLa cells stably expressing GFP alone were used as internal controls.

(F–H) Cell extracts from HeLa cells stably expressing GFP-WIPI2B were incubated for 3 hr with PI(5)P-containing liposomes (F and H) or PI(3)P-containing liposomes (G and H) before a pull-down experiment using the indicated beads. PS-containing liposomes were used as internal controls for both competition assays in (F and G).

(I) Lysates from HeLa cells stably expressing GFP-WIPI2B WT and GFP-WIPI2B FTTG mutant were incubated with agarose beads coated with PI(5)P and PI(3)P, eluted with SDS-PAGE sample buffer, and recovered proteins were assessed by western blotting using antibodies against GFP. See also Figure S6.

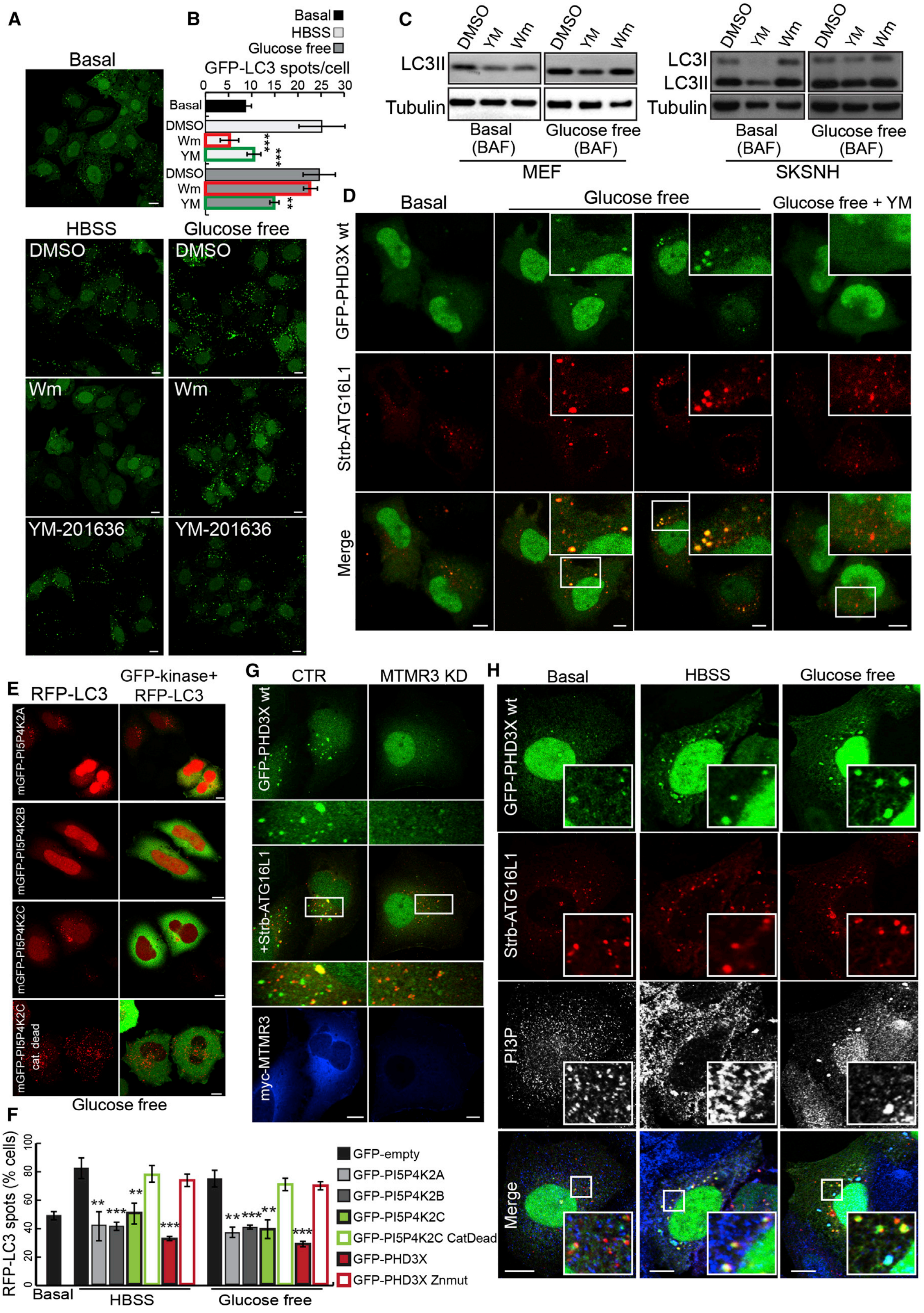

(legend on next page)

REFERENCES

Baskaran, S., Ragusa, M.J., Boura, E., and Hurley, J.H. (2012). Two-site recognition of phosphatidylinositol 3-phosphate by PROPPINs in autophagy. Mol. Cell *47*, 339–348.

Blommaart, E.F., Krause, U., Schellens, J.P., Vreeling-Sindelárová, H., and Meijer, A.J. (1997). The phosphatidylinositol 3-kinase inhibitors wortmannin and LY294002 inhibit autophagy in isolated rat hepatocytes. Eur. J. Biochem. *243*, 240–246.

Boya, P., Reggiori, F., and Codogno, P. (2013). Emerging regulation and functions of autophagy. Nat. Cell Biol. *15*, 713–720.

Bua, D.J., Martin, G.M., Binda, O., and Gozani, O. (2013). Nuclear phosphatidylinositol-5-phosphate regulates ING2 stability at discrete chromatin targets in response to DNA damage. Sci. Rep. *3*, 2137.

Clarke, J.H., Wang, M., and Irvine, R.F. (2010). Localization, regulation and function of type II phosphatidylinositol 5-phosphate 4-kinases. Adv. Enzyme Regul. *50*, 12–18.

Codogno, P., Mehrpour, M., and Proikas-Cezanne, T. (2012). Canonical and non-canonical autophagy: variations on a common theme of self-eating? Nat. Rev. Mol. Cell Biol. *13*, 7–12.

Davis, M.I., Sasaki, A.T., Shen, M., Emerling, B.M., Thorne, N., Michael, S., Pragani, R., Boxer, M., Sumita, K., Takeuchi, K., et al. (2013). A homogeneous, high-throughput assay for phosphatidylinositol 5-phosphate 4-kinase with a novel, rapid substrate preparation. PLoS ONE *8*, e54127.

de Lartigue, J., Polson, H., Feldman, M., Shokat, K., Tooze, S.A., Urbé, S., and Clague, M.J. (2009). PIKfyve regulation of endosome-linked pathways. Traffic *10*, 883–893.

Devereaux, K., Dall'Armi, C., Alcazar-Roman, A., Ogasawara, Y., Zhou, X., Wang, F., Yamamoto, A., De Camilli, P., and Di Paolo, G. (2013). Regulation of mammalian autophagy by class II and III PI 3-kinases through PI3P synthesis. PLoS ONE *8*, e76405.

Dooley, H.C., Razi, M., Polson, H.E., Girardin, S.E., Wilson, M.I., and Tooze, S.A. (2014). WIPI2 links LC3 conjugation with PI3P, autophagosome formation, and pathogen clearance by recruiting Atg12-5-16L1. Mol. Cell *55*, 238–252.

Er, E.E., Mendoza, M.C., Mackey, A.M., Rameh, L.E., and Blenis, J. (2013). AKT facilitates EGFR trafficking and degradation by phosphorylating and activating PIKfyve. Sci. Signal. *6*, ra45.

Fujita, N., Hayashi-Nishino, M., Fukumoto, H., Omori, H., Yamamoto, A., Noda, T., and Yoshimori, T. (2008). An Atg4B mutant hampers the lipidation of LC3 paralogues and causes defects in autophagosome closure. Mol. Biol. Cell *19*, 4651–4659.

Futter, C.E., Collinson, L.M., Backer, J.M., and Hopkins, C.R. (2001). Human VPS34 is required for internal vesicle formation within multivesicular endosomes. J. Cell Biol. *155*, 1251–1264.

Gozani, O., Karuman, P., Jones, D.R., Ivanov, D., Cha, J., Lugovskoy, A.A., Baird, C.L., Zhu, H., Field, S.J., Lessnick, S.L., et al. (2003). The PHD finger of the chromatin-associated protein ING2 functions as a nuclear phosphoinositide receptor. Cell *114*, 99–111.

Guittard, G., Gérard, A., Dupuis-Coronas, S., Tronchère, H., Mortier, E., Favre, C., Olive, D., Zimmermann, P., Payrastre, B., and Nunès, J.A. (2009). Cutting edge: Dok-1 and Dok-2 adaptor molecules are regulated by phosphatidylinositol 5-phosphate production in T cells. J. Immunol. *182*, 3974–3978.

Guittard, G., Mortier, E., Tronchère, H., Firaguay, G., Gérard, A., Zimmermann, P., Payrastre, B., and Nunès, J.A. (2010). Evidence for a positive role of PtdIns5P in T-cell signal transduction pathways. FEBS Lett. *584*, 2455–2460.

Harris, H., and Rubinsztein, D.C. (2011). Control of autophagy as a therapy for neurodegenerative disease. Nat. Rev. Neurol. *8*, 108–117.

Hill, E.V., Hudson, C.A., Vertommen, D., Rider, M.H., and Tavaré, J.M. (2010). Regulation of PIKfyve phosphorylation by insulin and osmotic stress. Biochem. Biophys. Res. Commun. *397*, 650–655.

Ikonomov, O.C., Sbrissa, D., Delvecchio, K., Xie, Y., Jin, J.P., Rappolee, D., and Shisheva, A. (2011). The phosphoinositide kinase PIKfyve is vital in early embryonic development: preimplantation lethality of PIKfyve-/- embryos but normality of PIKfyve+/- mice. J. Biol. Chem. *286*, 13404–13413.

Jefferies, H.B., Cooke, F.T., Jat, P., Boucheron, C., Koizumi, T., Hayakawa, M., Kaizawa, H., Ohishi, T., Workman, P., Waterfield, M.D., and Parker, P.J. (2008). A selective PIKfyve inhibitor blocks PtdIns(3,5)P(2) production and disrupts endomembrane transport and retroviral budding. EMBO Rep. *9*, 164–170.

Jeffries, T.R., Dove, S.K., Michell, R.H., and Parker, P.J. (2004). PtdIns-specific MPR pathway association of a novel WD40 repeat protein, WIPI49. Mol. Biol. Cell *15*, 2652–2663.

Kabeya, Y., Mizushima, N., Ueno, T., Yamamoto, A., Kirisako, T., Noda, T., Kominami, E., Ohsumi, Y., and Yoshimori, T. (2000). LC3, a mammalian homologue of yeast Apg8p, is localized in autophagosome membranes after processing. EMBO J. *19*, 5720–5728.

Li, X., Wang, X., Zhang, X., Zhao, M., Tsang, W.L., Zhang, Y., Yau, R.G., Weisman, L.S., and Xu, H. (2013). Genetically encoded fluorescent probe to visualize intracellular phosphatidylinositol 3,5-bisphosphate localization and dynamics. Proc. Natl. Acad. Sci. USA *110*, 21165–21170.

Liu, Y., Lai, Y.C., Hill, E.V., Tyteca, D., Carpentier, S., Ingvaldsen, A., Vertommen, D., Lantier, L., Foretz, M., Dequiedt, F., et al. (2013). Phosphatidylinositol 3-phosphate 5-kinase (PIKfyve) is an AMPK target participating in contraction-stimulated glucose uptake in skeletal muscle. Biochem. J. *455*, 195–206.

Mauthe, M., Jacob, A., Freiberger, S., Hentschel, K., Stierhof, Y.D., Codogno, P., and Proikas-Cezanne, T. (2011). Resveratrol-mediated autophagy requires WIPI-1-regulated LC3 lipidation in the absence of induced phagophore formation. Autophagy *7*, 1448–1461.

McAlpine, F., Williamson, L.E., Tooze, S.A., and Chan, E.Y. (2013). Regulation of nutrient-sensitive autophagy by uncoordinated 51-like kinases 1 and 2. Autophagy *9*, 361–373.

Figure 7. PI(5)P Is a Master Regulator of Autophagy in Response to Glucose Starvation

(A) HeLa cells stably expressing GFP-LC3 were shifted to starvation media (HBSS or glucose-free DMEM) for 4 hr (in the presence of 200 nM Wm or 100 nM YM-201636).

(B) Quantification of numbers of GFP-LC3 vesicles per cell in HeLa cells stably expressing GFP-LC3 treated as in (A) is shown in the graph (mean ± SEM).

(C) Western blot analysis of LC3-II and tubulin levels in MEF and SKNSH cells were treated as in (A) in complete media or glucose-free media in the presence of 100 nM YM-201636, 200 nM Wm, and 400 nM BAF for 4 hr.

(D) HeLa cells transfected with GFP-PHD3X and Strawberry-ATG16L1 for 16 hr were left in complete media (basal) or glucose-free media (glucose free) for 4 hr in the presence or absence of 100 nM YM-201636, then fixed and imaged on a confocal microscope.

(E and F) HeLa cells transfected with GFP-PI5P4K2A, 2B, 2C, or PI5P4K2C catalytic dead and RFP-LC3 for 30 hr (E) were starved for glucose for 4 hr, then fixed and imaged on a confocal microscope. Bar, 10 μm. (F) Quantification of cells (percentage of total) showing more than ten autophagic vesicles (RFP-LC3 vesicles) in the different conditions from (F) is shown in the graph; n = 200 cells (mean ± SEM).

(G) HeLa cells treated with CTR or MTMR3 siRNA for 5 days were transfected for the last 16 hr with GFP-PHD3X, Strb-ATG16L1, and myc-MTMR3 WT. Cells were starved for glucose for 4 hr, then fixed, stained with anti-myc antibodies, and imaged on a confocal microscope. Bar, 10 μm.

(H) HeLa cells transfected for 16 hr with GFP-PHD3X and Strb-ATG16L1 were starved for 4 hr in HBSS or glucose-free media, then fixed, stained for PI(3)P antibodies, and imaged on a confocal microscope. Bar, 10 μm. See also Figure S7.

Moreau, K., Ravikumar, B., Puri, C., and Rubinsztein, D.C. (2012). Arf6 promotes autophagosome formation via effects on phosphatidylinositol 4,5-bisphosphate and phospholipase D. J. Cell Biol. *196*, 483–496.

Nakatogawa, H., Suzuki, K., Kamada, Y., and Ohsumi, Y. (2009). Dynamics and diversity in autophagy mechanisms: lessons from yeast. Nat. Rev. Mol. Cell Biol. *10*, 458–467.

Narain, Y., Wyttenbach, A., Rankin, J., Furlong, R.A., and Rubinsztein, D.C. (1999). A molecular investigation of true dominance in Huntington's disease. J. Med. Genet. *36*, 739–746.

Nixon, R.A. (2013). The role of autophagy in neurodegenerative disease. Nat. Med. *19*, 983–997.

Oppelt, A., Lobert, V.H., Haglund, K., Mackey, A.M., Rameh, L.E., Liestøl, K., Schink, K.O., Pedersen, N.M., Wenzel, E.M., Haugsten, E.M., et al. (2013). Production of phosphatidylinositol 5-phosphate via PIKfyve and MTMR3 regulates cell migration. EMBO Rep. *14*, 57–64.

Oppelt, A., Haugsten, E.M., Zech, T., Danielsen, H.E., Sveen, A., Lobert, V.H., Skotheim, R.I., and Wesche, J. (2014). PIKfyve, MTMR3 and their product PtdIns5P regulate cancer cell migration and invasion through activation of Rac1. Biochem. J. *461*, 383–390.

Petiot, A., Ogier-Denis, E., Blommaart, E.F., Meijer, A.J., and Codogno, P. (2000). Distinct classes of phosphatidylinositol 3′-kinases are involved in signaling pathways that control macroautophagy in HT-29 cells. J. Biol. Chem. *275*, 992–998.

Petiot, A., Faure, J., Stenmark, H., and Gruenberg, J. (2003). PI3P signaling regulates receptor sorting but not transport in the endosomal pathway. J. Cell Biol. *162*, 971–979.

Plowey, E.D., Cherra, S.J., 3rd, Liu, Y.J., and Chu, C.T. (2008). Role of autophagy in G2019S-LRRK2-associated neurite shortening in differentiated SH-SY5Y cells. J. Neurochem. *105*, 1048–1056.

Polson, H.E., de Lartigue, J., Rigden, D.J., Reedijk, M., Urbé, S., Clague, M.J., and Tooze, S.A. (2010). Mammalian Atg18 (WIPI2) localizes to omegasome-anchored phagophores and positively regulates LC3 lipidation. Autophagy *6*, 506–522.

Rameh, L.E., Tolias, K.F., Duckworth, B.C., and Cantley, L.C. (1997). A new pathway for synthesis of phosphatidylinositol-4,5-bisphosphate. Nature *390*, 192–196.

Ravikumar, B., Duden, R., and Rubinsztein, D.C. (2002). Aggregate-prone proteins with polyglutamine and polyalanine expansions are degraded by autophagy. Hum. Mol. Genet. *11*, 1107–1117.

Ravikumar, B., Imarisio, S., Sarkar, S., O'Kane, C.J., and Rubinsztein, D.C. (2008). Rab5 modulates aggregation and toxicity of mutant huntingtin through macroautophagy in cell and fly models of Huntington disease. J. Cell Sci. *121*, 1649–1660.

Row, P.E., Reaves, B.J., Domin, J., Luzio, J.P., and Davidson, H.W. (2001). Overexpression of a rat kinase-deficient phosphoinositide 3-kinase, Vps34p, inhibits cathepsin D maturation. Biochem. J. *353*, 655–661.

Rubinsztein, D.C., Cuervo, A.M., Ravikumar, B., Sarkar, S., Korolchuk, V., Kaushik, S., and Klionsky, D.J. (2009). In search of an "autophagomometer". Autophagy *5*, 585–589.

Sarkes, D., and Rameh, L.E. (2010). A novel HPLC-based approach makes possible the spatial characterization of cellular PtdIns5P and other phosphoinositides. Biochem. J. *428*, 375–384.

Sbrissa, D., Ikonomov, O.C., and Shisheva, A. (1999). PIKfyve, a mammalian ortholog of yeast Fab1p lipid kinase, synthesizes 5-phosphoinositides. Effect of insulin. J. Biol. Chem. *274*, 21589–21597.

Sbrissa, D., Ikonomov, O.C., and Shisheva, A. (2002). Phosphatidylinositol 3-phosphate-interacting domains in PIKfyve. Binding specificity and role in PIKfyve. Endomenbrane localization. J. Biol. Chem. *277*, 6073–6079.

Sbrissa, D., Ikonomov, O.C., Filios, C., Delvecchio, K., and Shisheva, A. (2012). Functional dissociation between PIKfyve-synthesized PtdIns5P and PtdIns(3,5)P2 by means of the PIKfyve inhibitor YM201636. Am. J. Physiol. Cell Physiol. *303*, C436–C446.

Scarlatti, F., Maffei, R., Beau, I., Codogno, P., and Ghidoni, R. (2008). Role of non-canonical Beclin 1-independent autophagy in cell death induced by resveratrol in human breast cancer cells. Cell Death Differ. *15*, 1318–1329.

Schink, K.O., Raiborg, C., and Stenmark, H. (2013). Phosphatidylinositol 3-phosphate, a lipid that regulates membrane dynamics, protein sorting and cell signalling. BioEssays *35*, 900–912.

Shisheva, A. (2013). PtdIns5P: news and views of its appearance, disappearance and deeds. Arch. Biochem. Biophys. *538*, 171–180.

Siddhanta, U., McIlroy, J., Shah, A., Zhang, Y., and Backer, J.M. (1998). Distinct roles for the p110alpha and hVPS34 phosphatidylinositol 3′-kinases in vesicular trafficking, regulation of the actin cytoskeleton, and mitogenesis. J. Cell Biol. *143*, 1647–1659.

Taguchi-Atarashi, N., Hamasaki, M., Matsunaga, K., Omori, H., Ktistakis, N.T., Yoshimori, T., and Noda, T. (2010). Modulation of local PtdIns3P levels by the PI phosphatase MTMR3 regulates constitutive autophagy. Traffic *11*, 468–478.

Vergne, I., Roberts, E., Elmaoued, R.A., Tosch, V., Delgado, M.A., Proikas-Cezanne, T., Laporte, J., and Deretic, V. (2009). Control of autophagy initiation by phosphoinositide 3-phosphatase Jumpy. EMBO J. *28*, 2244–2258.

Viaud, J., Boal, F., Tronchère, H., Gaits-Iacovoni, F., and Payrastre, B. (2014a). Phosphatidylinositol 5-phosphate: a nuclear stress lipid and a tuner of membranes and cytoskeleton dynamics. Bioessays. *36*, 260–272.

Viaud, J., Lagarrigue, F., Ramel, D., Allart, S., Chicanne, G., Ceccato, L., Courilleau, D., Xuereb, J.M., Pertz, O., Payrastre, B., and Gaits-Iacovoni, F. (2014b). Phosphatidylinositol 5-phosphate regulates invasion through binding and activation of Tiam1. Nat. Commun. *5*, 4080.

Whiteford, C.C., Brearley, C.A., and Ulug, E.T. (1997). Phosphatidylinositol 3,5-bisphosphate defines a novel PI 3-kinase pathway in resting mouse fibroblasts. Biochem. J. *323*, 597–601.

Wilcox, A., and Hinchliffe, K.A. (2008). Regulation of extranuclear PtdIns5P production by phosphatidylinositol phosphate 4-kinase 2alpha. FEBS Lett. *582*, 1391–1394.

Williams, A., Sarkar, S., Cuddon, P., Ttofi, E.K., Saiki, S., Siddiqi, F.H., Jahreiss, L., Fleming, A., Pask, D., Goldsmith, P., et al. (2008). Novel targets for Huntington's disease in an mTOR-independent autophagy pathway. Nat. Chem. Biol. *4*, 295–305.

Zhou, X., Wang, L., Hasegawa, H., Amin, P., Han, B.X., Kaneko, S., He, Y., and Wang, F. (2010). Deletion of PIK3C3/Vps34 in sensory neurons causes rapid neurodegeneration by disrupting the endosomal but not the autophagic pathway. Proc. Natl. Acad. Sci. USA *107*, 9424–9429.

Zhu, J.H., Horbinski, C., Guo, F., Watkins, S., Uchiyama, Y., and Chu, C.T. (2007). Regulation of autophagy by extracellular signal-regulated protein kinases during 1-methyl-4-phenylpyridinium-induced cell death. Am. J. Pathol. *170*, 75–86.

Zolov, S.N., Bridges, D., Zhang, Y., Lee, W.W., Riehle, E., Verma, R., Lenk, G.M., Converso-Baran, K., Weide, T., Albin, R.L., et al. (2012). In vivo, Pikfyve generates PI(3,5)P2, which serves as both a signaling lipid and the major precursor for PI5P. Proc. Natl. Acad. Sci. USA *109*, 17472–17477.

Zou, J., Zhang, C., Marjanovic, J., Kisseleva, M.V., Majerus, P.W., and Wilson, M.P. (2012). Myotubularin-related protein (MTMR) 9 determines the enzymatic activity, substrate specificity, and role in autophagy of MTMR8. Proc. Natl. Acad. Sci. USA *109*, 9539–9544.

Molecular Cell, Volume *57*

Supplemental Information

PI(5)P Regulates Autophagosome Biogenesis

Mariella Vicinanza, Viktor I. Korolchuk, Avraham Ashkenazi,
Claudia Puri, Fiona M. Menzies, Jonathan H. Clarke, and David C. Rubinsztein

Supplemental Data

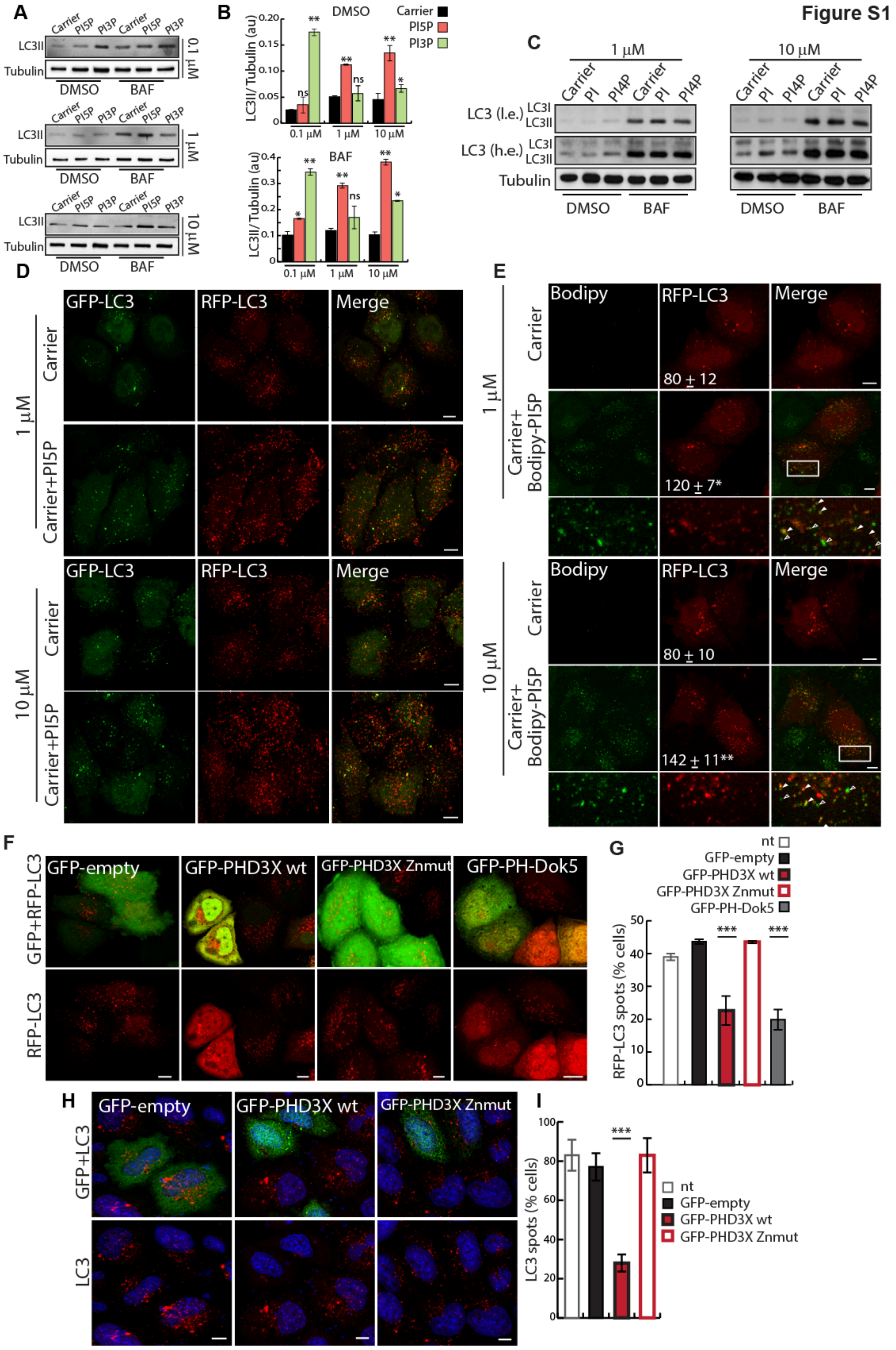

Figure S1: Maniplation of PI(5)P levels affects autophagosome numbers, Related to Figure 1. (**A**) Western blot analysis of LC3-II and tubulin levels in HeLa cells after 1h treatment with carrier alone or in combination with PI(5)P or PI(3)P di-C16 at indicated concentrations, in the absence and presence of 400 nM bafilomycin A1 (BAF, treated in combination with lipids for 1 h). (**B)**. Quantification of the band intensities from three experiments represented as LC3-II/tubulin ratio is shown in the graph; (mean±s.e.m). (**C**) Representative blots of LC3-II and tubulin levels in HeLa cells after 1h treatment with carrier alone or in combination with PI and PI(4)P di-C16 at indicated concentrations, in presence of 400 nM bafilomycin A1 (BAF, treated in combination with lipids for 1 h). Note that LC3-I is detectable with high exposure (h.e.). (**D**) HeLa cells stably expressing GFP-mRFP-LC3 were loaded with indicated concentrations of PI(5)P during which they were left in starvation media (HBSS). (**E**) HeLa cells transfected with RFP-LC3 were exposed to indicated concentrations of BODIPY-labelled-PI(5)P for 1h in starvation media (HBSS) and followed by live cell imaging for 10 min. For the negative control, medium was combined with carrier only and added to the cells. Numbers of RFP-LC3 structures per cell were quantified using the Analyse Particles plugin in ImageJ in at least 10 cells per condition from two independent experiments. (mean±s.e.m.). (**F,G**) HeLa cells transfected for 30 h with GFP-empty, GFP-PHD3X, GFP-PHD3X Znmut or GFP-PH-Dok5 combined with RFP-LC3, and were incubated in starvation media (HBSS) and fixed after 1 h. (**H,I**) HeLa cells transfected for 30 h with GFP-empty, GFP-PHD3X, GFP-PHD3X Znmut were then grown in HBSS for 1 h, after which the cells were fixed in methanol and immunostained for endogenous LC3 and anti-GFP. (**G,I**) Quantification of cells (% of total) showing more than 10 autophagic vesicles (LC3 or RFP-LC3 vesicles) in the different conditions is shown in the graph. nt, not transfected cells, n = 200 cells. (mean±s.e.m., t-test compared to GFP-transfected cells). Bar 10 μm.

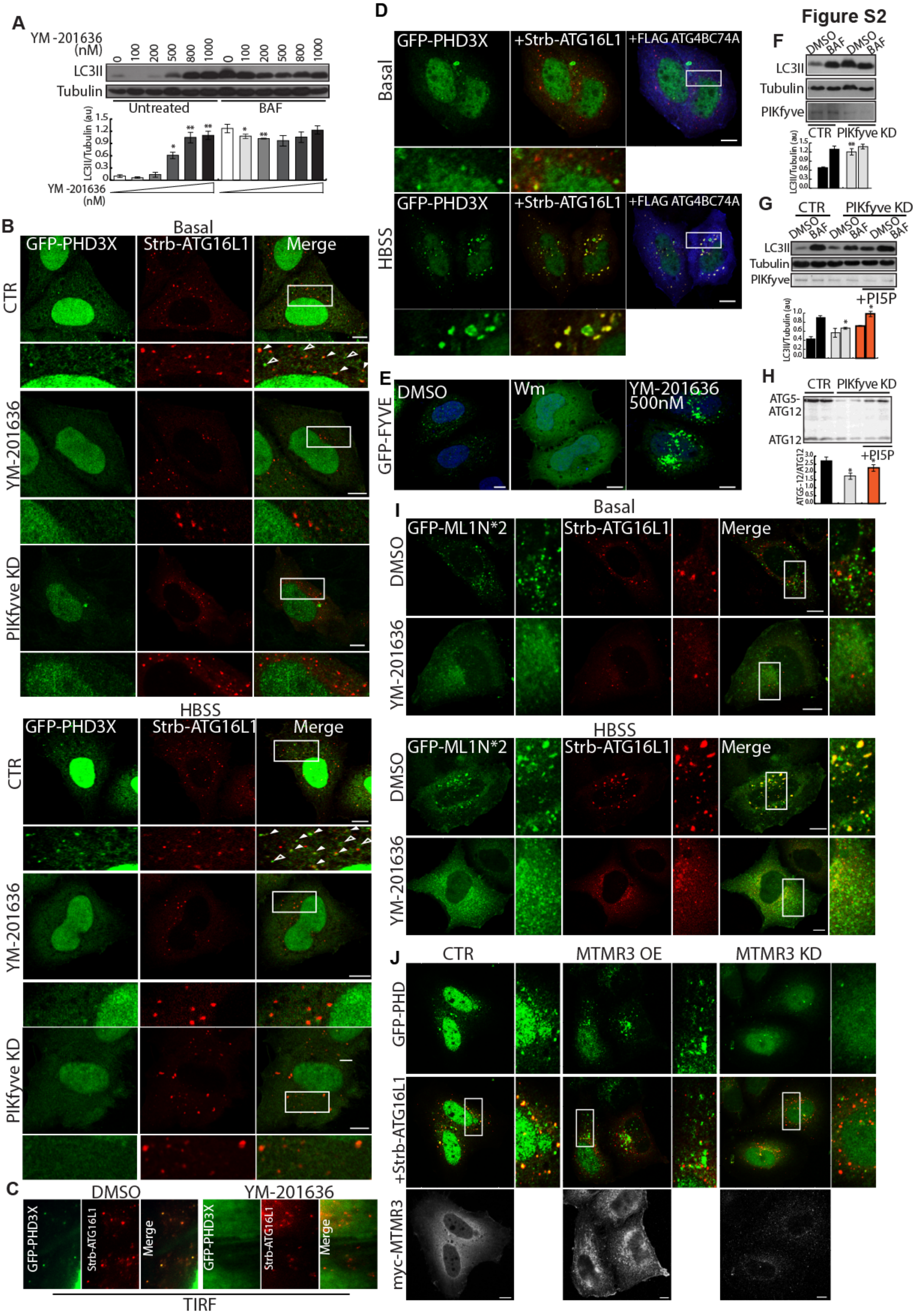

Figure S2
A
YM -201636 (nM)
0 100 200 500 800 1000 0 100 200 500 800 1000
LC3II
Tubulin
Untreated
BAF
LC3II/Tubulin (au)
YM -201636 (nM)
B
Basal
GFP-PHD3X
Strb-ATG16L1
Merge
CTR
YM-201636
PIKfyve KD
HBSS
C
DMSO
YM-201636
Merge
TIRF
D
GFP-PHD3X
+Strb-ATG16L1
+FLAG ATG4BC74A
Basal
HBSS
E
DMSO
Wm
YM-201636 500nM
GFP-FYVE
F
DMSO BAF DMSO BAF
LC3II
Tubulin
PIKfyve
CTR PIKfyve KD
G
CTR PIKfyve KD
+PI5P
H
ATG5-ATG12
ATG12
ATG5-12/ATG12
I
GFP-ML1N*2
J
CTR
MTMR3 OE
MTMR3 KD
GFP-PHD
+Strb-ATG16L1
myc-MTMR3

Figure S2: PIKfyve provides PI(5)P localized to autophagosomes, Related to Figure 2. (**A**) HeLa cells were treated with DMSO or increasing concentrations of YM-201636 for 24 h in the presence or absence of 400 nM BAF. Samples were prepared for SDS-PAGE and immunoblotted for LC3 and tubulin. The levels of LC3-II were normalised to tubulin and quantified (mean±s.e.m.). (**B**) HeLa cells transfected with GFP-PHD3X and Strawberry-ATG16L1 for 16 h were left in complete media (basal) or starvation media (HBSS) for 1h in presence or absence of 100 nM YM-201636, then fixed and imaged on confocal microscope. For PIKfyve silencing, cells were treated with PIKfyve siRNA for 5 days and transfected on day 4. (**C**) PI(5)P localization on ATG16L1-positive structures close to the plasma-membrane was analysed by TIRF imaging of GFP-PHD3X. Inhibition of PIKfyve activity by 100 nM YM-201636 caused loss of GFP-PHD3X dots. (**D**) HeLa cells transfected with flag-tagged ATG4BC74A, GFP-PHD3X and Strawberry-ATG16L1 for 16 h were left in complete media (basal) or starvation media (HBSS) for 1h, then fixed, stained with anti-flag antibodies and imaged on confocal microscope. (**E**) HeLa cells transfected with GFP-FYVE (a PI(3)P probe) for 16 h were treated with DMSO, 200nM Wm or 500 nM YM-201636 for 2 h. Wm treatment ablates the GFP-FYVE signal on vesicles, as a control. (**F-H**) HeLa cells were treated with control (CTR) or PIKfyve targeting siRNA for 5 days and treated with or without 200 nM BAF for 16 h in complete tissue culture medium (**F**) or for 2h in HBSS medium (**G**) or transfected with HA-ATG12 and ATG5 (**H**). In (**G**) and (**H**) cells were loaded with exogenous PI(5)P (10 μM) for 2h. Cells were subjected to western blot analysis with anti-LC3-II and anti-tubulin antibodies (LC3-II levels relative to tubulin were quantified) (**F-G)** and anti-HA antibody (to detect free ATG12 and the ATG5-ATG12 complex) **(H).** (**I**) HeLa cells transfected with GFP-ML1N*2 (tandem repeat of N-terminal segment of mucolipin1) and Strawberry-ATG16L1 for 16 h were left in complete media (basal) or starvation media (HBSS) for 1h in presence or absence of 100 nM YM-201636, then fixed and imaged on confocal microscope. Bar 10 μm. (**J**) HeLa cells transfected for 5 days with two rounds of control or MTMR3 siRNA were transfected with GFP-PHD3X, Strb-ATG16L1 and myc-tagged MTMR3 for last 12 h, starved for 1h in HBSS media and then fixed, labelled with anti-myc antibodies and imaged on confocal microscope. For MTMR3 over-expression, cells were transfected for 30 h. Bar 10 μm.

Figure S3

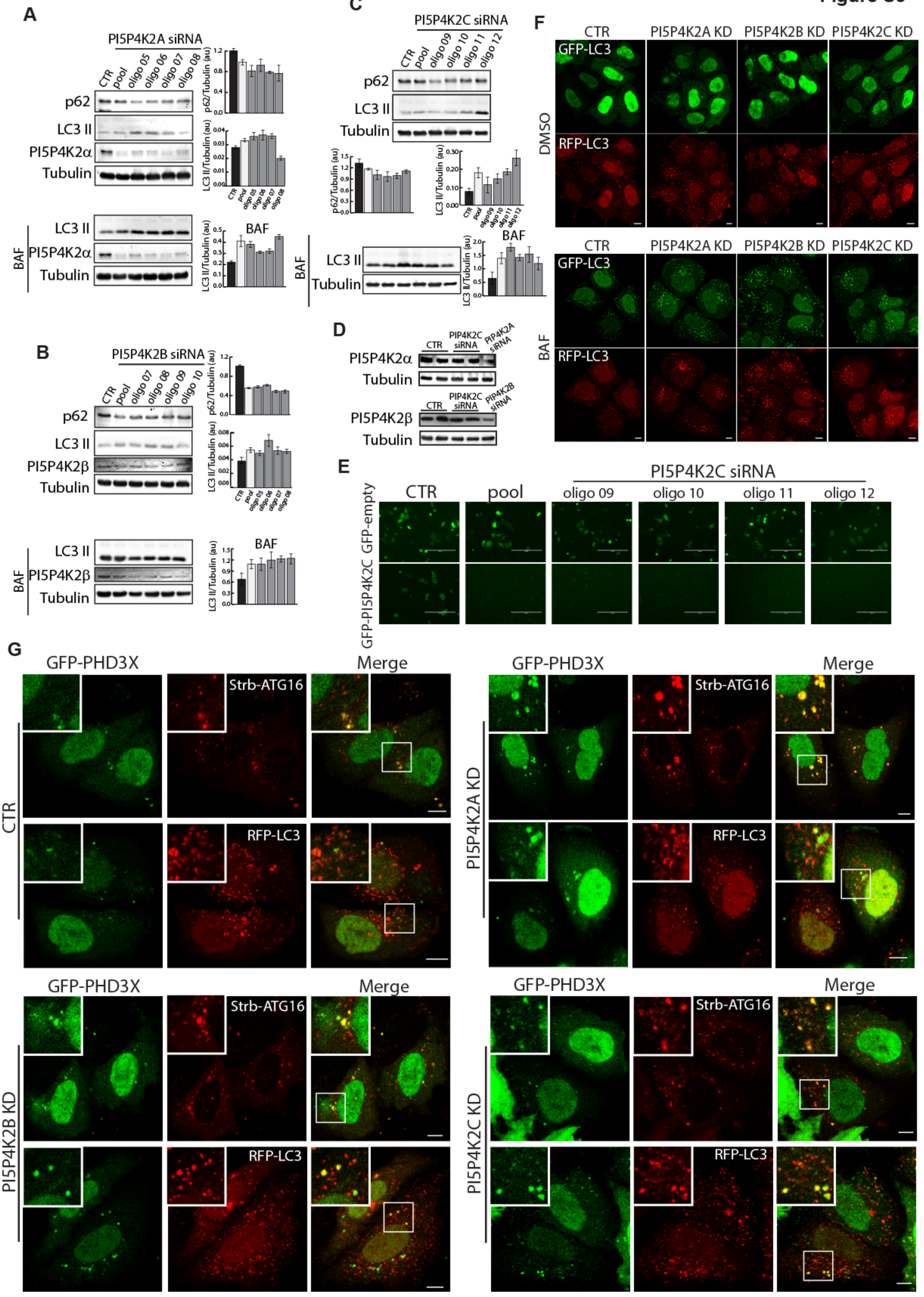
A
PI5P4K2A siRNA
CTR
pool
oligo 05
oligo 06
oligo 07
oligo 08
p62
LC3 II
PI5P4K2α
Tubulin
BAF
B
PI5P4K2B siRNA
PI5P4K2β
C
PI5P4K2C siRNA
oligo 09
oligo 10
oligo 11
oligo 12
D
PIP4K2C siRNA
PIP4K2A siRNA
PIP4K2B siRNA
E
GFP-empty
GFP-PI5P4K2C
F
PI5P4K2A KD
PI5P4K2B KD
PI5P4K2C KD
GFP-LC3
RFP-LC3
DMSO
G
GFP-PHD3X
Strb-ATG16
Merge
PI5P4K2A KD
PI5P4K2B KD
PI5P4K2C KD

Figure S3: PI5P4K2s knockdown affects PI(5)P levels on autophagosomes and clearance of autophagic substrates, Related to Figure 3. The specificity of PI5P4K2s KD effects on the levels of LC3-II and p62, were confirmed by deconvolution of PI5P4K2A, 2B and 2C siRNA smartpools. (**A-E**) HeLa cells were transfected with a set of 4 siRNA (pool) or individual siRNA oligonucleotides for 5 days and treated with or without 200 nM BAF for 16 h in complete tissue culture medium. Cells were immunoblotted and LC3-II and p62 levels relative to tubulin were quantified (graphs on the right). (**D**) PI5P4K2C silencing did not affect PI5P4K2A and PI5P4K2B levels. (**E**) The efficiency of PI5P4K2C knock-down was visualized by disappearance of GFP-tagged kinases using a fluorescence microscope (Evos FL imaging system) since a specific antibody was not available. Bar 200 μm. Note, decreased levels p62 in PI5P4K2s KD cells supports a functional role for PI(5)P and PI5P4K2s in autophagic substrate degradation. (**F**) HeLa cells stably expressing GFP–mRFP–LC3 were transfected for 5 days with two rounds of control, PI5P4K2A, 2B and 2C siRNA, during which they were either left untreated or treated with 200 nM BAF for the last 16 h. Cells were then fixed and analysed on a confocal microscope. (**G**) HeLa cells transfected with two rounds of control, PI5P4K2A, 2B and 2C siRNA for 5 days and with GFP-PHD3X, and RFP-LC3 or Strb-ATG16L1 for the last 16 h were incubated in HBSS for 1h and observed under a live cell confocal microscope. Bar 10 μm.

Figure S4

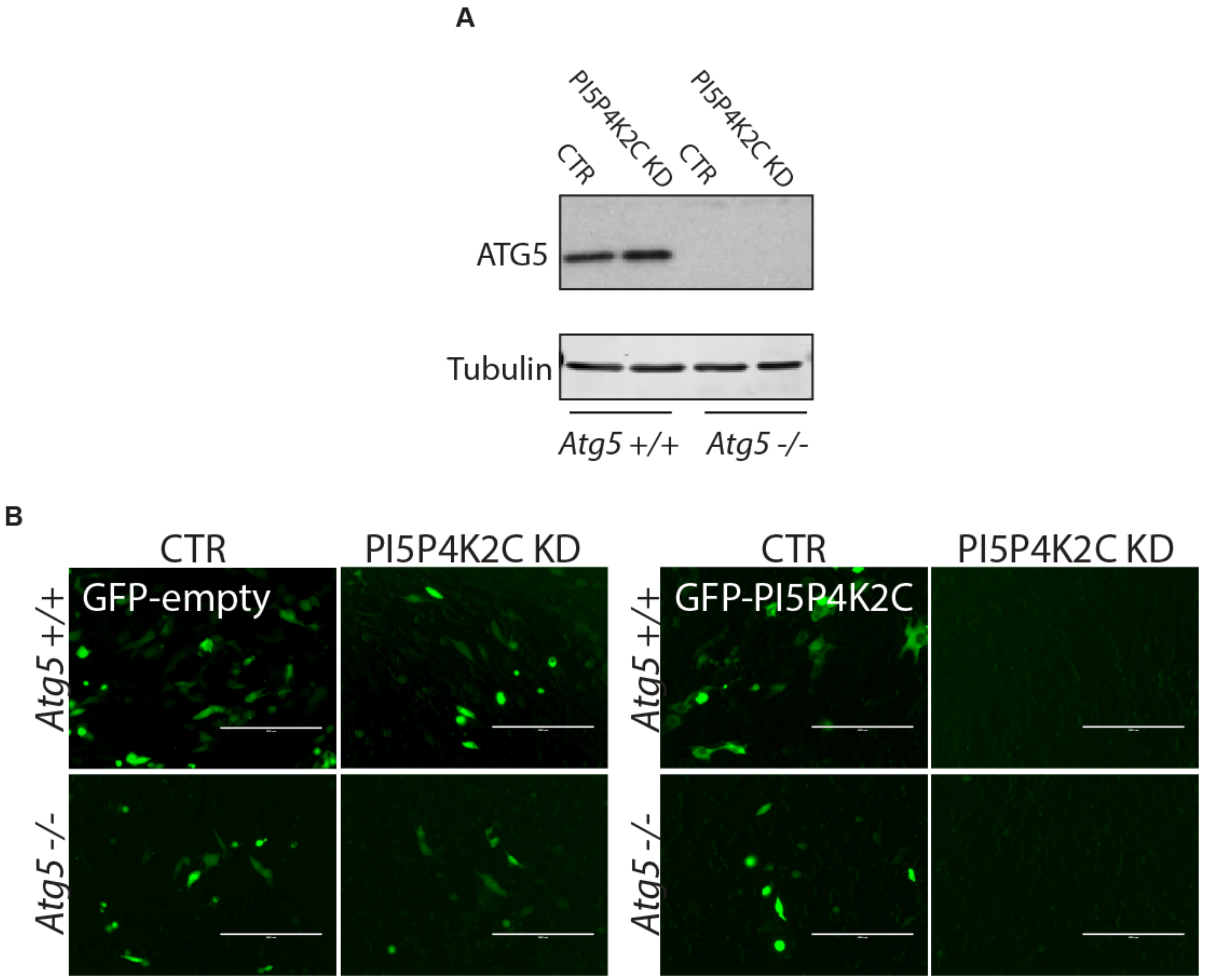

Figure S4: Increased PI(5)P levels accelerate autophagic substrate clearance, Related to Figure 4. *Atg5* $^{+/+}$ and *Atg5* $^{-/-}$ MEF cells were treated with PI5P4K2C siRNAs (oligo 10+oligo12) for 5 days and co-transfected with EGFP-httQ74 for the last 48 h. Western blot analysis of ATG5 levels are shown in (**A**) and efficiency of PI5P4K2C knock-down was visualized by disappearance of GFP-tagged kinases using a fluorescence microscope (Evos FL imaging system) (**B**). Bar 200 μm.

Figure S5

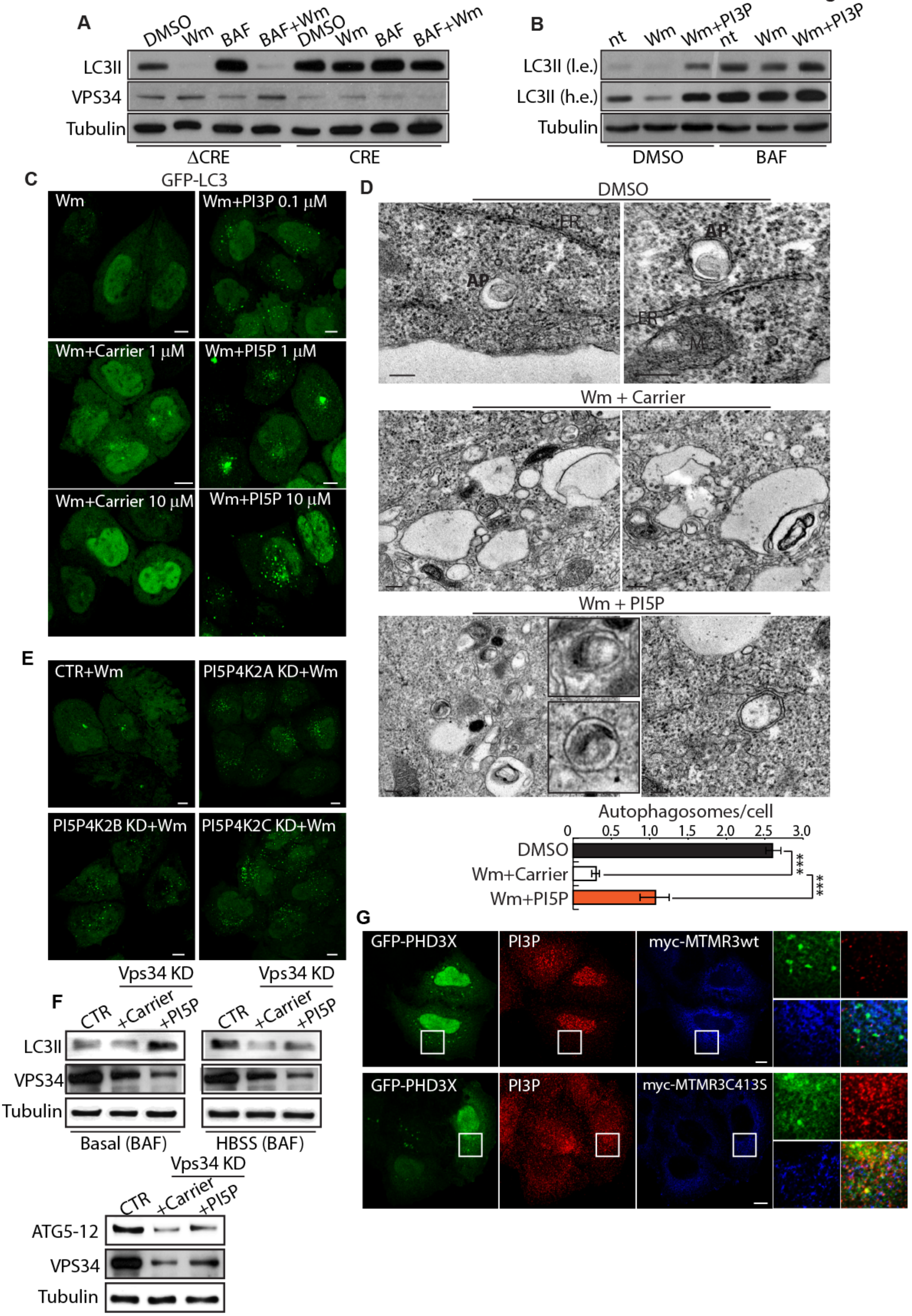
A
DMSO Wm BAF BAF+Wm DMSO Wm BAF BAF+Wm
LC3II
VPS34
Tubulin
ΔCRE
CRE
B
nt Wm Wm+PI3P nt Wm Wm+PI3P
LC3II (l.e.)
LC3II (h.e.)
Tubulin
DMSO
BAF
C
GFP-LC3
Wm
Wm+PI3P 0.1 µM
Wm+Carrier 1 µM
Wm+PI5P 1 µM
Wm+Carrier 10 µM
Wm+PI5P 10 µM
D
DMSO
ER
AP
AP
ER
M
Wm + Carrier
Wm + PI5P
Autophagosomes/cell
0 0.5 1.0 1.5 2.0 2.5 3.0
DMSO
Wm+Carrier
Wm+PI5P
***
***
E
CTR+Wm
PI5P4K2A KD+Wm
PI5P4K2B KD+Wm
PI5P4K2C KD+Wm
F
Vps34 KD
Vps34 KD
CTR +Carrier +PI5P
CTR +Carrier +PI5P
LC3II
VPS34
Tubulin
Basal (BAF)
HBSS (BAF)
Vps34 KD
CTR +Carrier +PI5P
ATG5-12
VPS34
Tubulin
G
GFP-PHD3X
PI3P
myc-MTMR3wt
GFP-PHD3X
PI3P
myc-MTMR3C413S

Figure S5: PI(5)P rescues autophagosome formation in Wm-treated cells, Related to Figure 5. (**A**) *Vps34*Flox/Flox MEFs were infected with lentiviruses either expressing full-length Cre recombinase (CRE) or not (Δ) for 12 days. Cells were incubated in HBSS in presence or absence of 200 nM Wm. Western blot analysis of LC3-II, tubulin and VPS34 levels are shown. (**B**) HeLa cells were loaded for 2 h with carrier alone, or combined with 0.1 μM PI(3)P, in presence of 200 nM Wm and DMSO or BAF in HBSS. (**C**) HeLa cells stably expressing GFP–LC3 were pre-treated with 200 nM Wm for 2h in complete medium, then loaded for 1 h with carrier alone, or combined with PI(5)P or PI(3)P at indicated concentrations, in presence of 200 nM Wm and then shifted to HBSS medium in the presence of Wm for 2 h and imaged on a confocal microscope. (**D**) HeLa cells were loaded for 2 h with carrier alone, or combined with 10 μM PI(5)P, in presence of 200 nM Wm in HBSS and processed for Electron-microscopy. Morphometrical analysis was performed counting autophagosome structures in 40 random cells profile for each condition in two independent experiments. AP, autophagosome, ER, endoplasmic reticulum, M, mitochondria. (***$p<0.001$, t-test), Bar 300nm. (**E**) HeLa cells stably expressing GFP–LC3 transfected for 5 days with two rounds of control, PI5P4K2A, 2B and 2C siRNA were pre-treated with 200 nM Wm for 2 h in complete medium and then shifted to HBSS medium in the presence of Wm for 2 h and imaged on a confocal microscope. Bar 10 μm. (**F**) HeLa cells transfected for 3 days with one round of control and VPS34 siRNA were loaded for 2h with carrier alone, or combined with 10 μM PI(5)P in presence of 400 nM BAF in complete medium or HBSS. Western blot analysis of LC3-II, tubulin, ATG12 and VPS34 protein levels are shown. (**G**) HeLa cells were transfected with GFP-PHD3X and myc-MTMR3wt or myc-MTMR3C314S, incubated for 1h in HBSS and processed for immunofluorescence analysis and labelling of PI(3)P (see Supplemental Experimental Procedures).

Figure S6

A

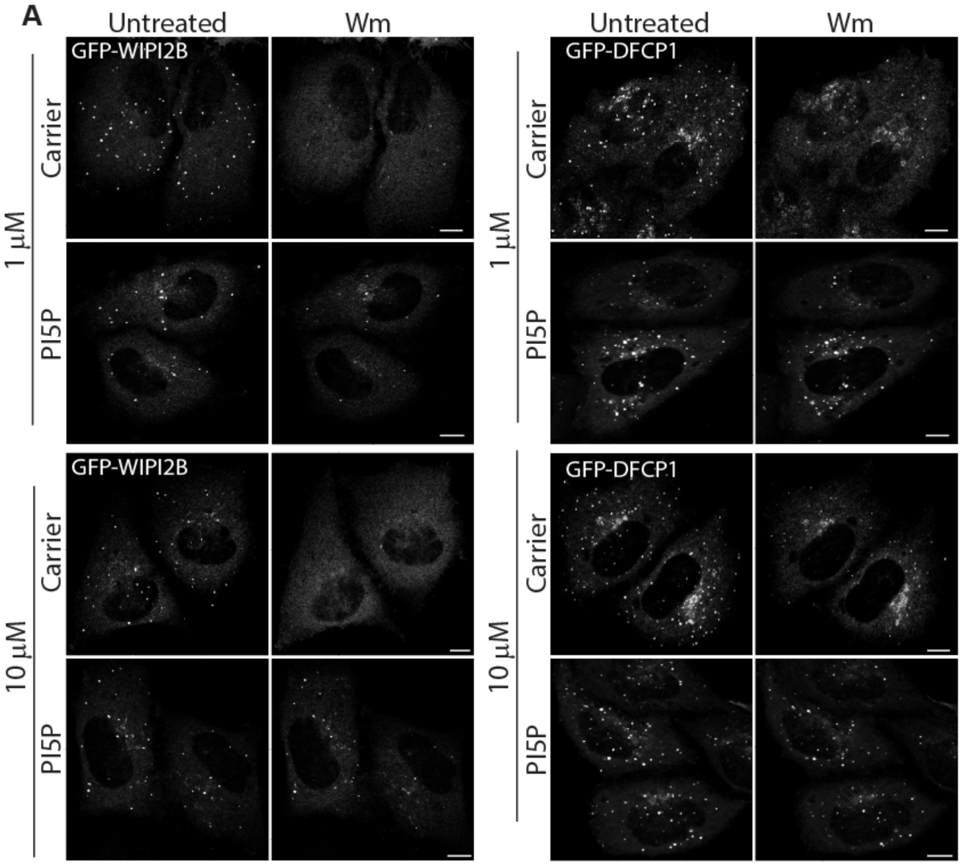

C

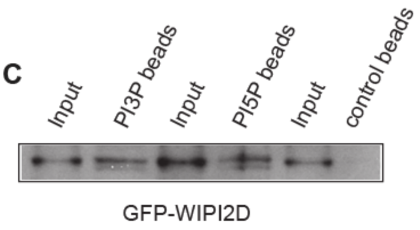

B

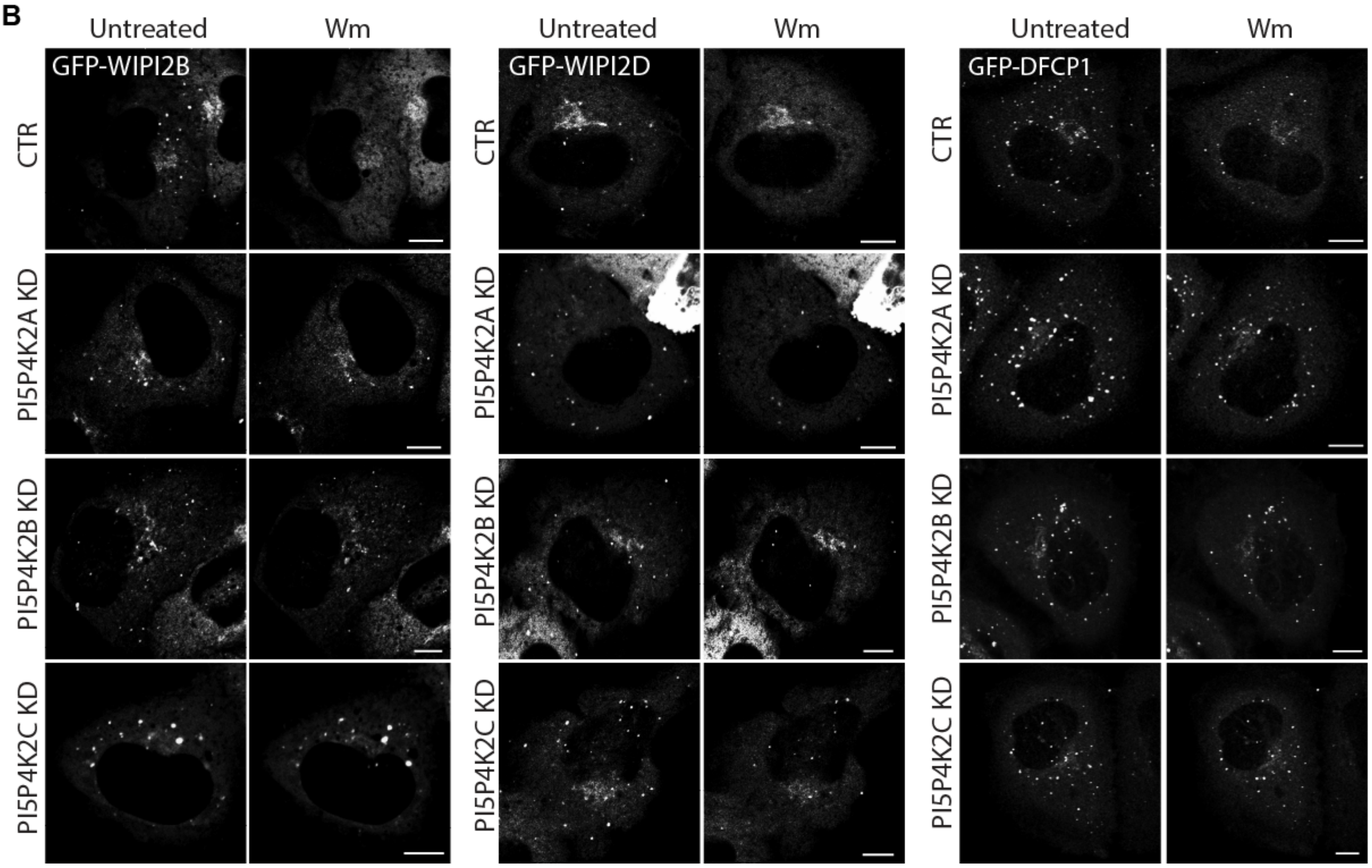

Figure S6: PI(5)P stabilizes WIPI2 and DFCP1 on membranes in Wm-treated cells, Related to Figure 6. (**A**) HeLa cells transfected with GFP-WIPI2B or GFP-DFCP1 were loaded for 1 h with carrier alone or combined with PI(5)P at indicated concentrations and incubated with 200 nM Wm in HBSS media and structures tracked by time-lapse microscopy for 10 minutes after the addition of Wm. (**B**) HeLa cells transfected for 5 days with two rounds of control, PI5P4K2A, 2B and 2C siRNA were transfected with GFP-WIPI2B, GFP-WIPI2D and GFP-DFCP1 for the last 16 h, starved in HBSS media for 1h and incubated with 200 nM Wm in HBSS. Bar 10 μm. (**C**) Lysates from HeLa cells transiently expressing GFP-WIPI2D were incubated with agarose beads coated with PI, PI(5)P and PI(3)P, eluted with SDS-PAGE sample buffer and recovered proteins were assessed by western blotting using antibodies against GFP.

Figure S7

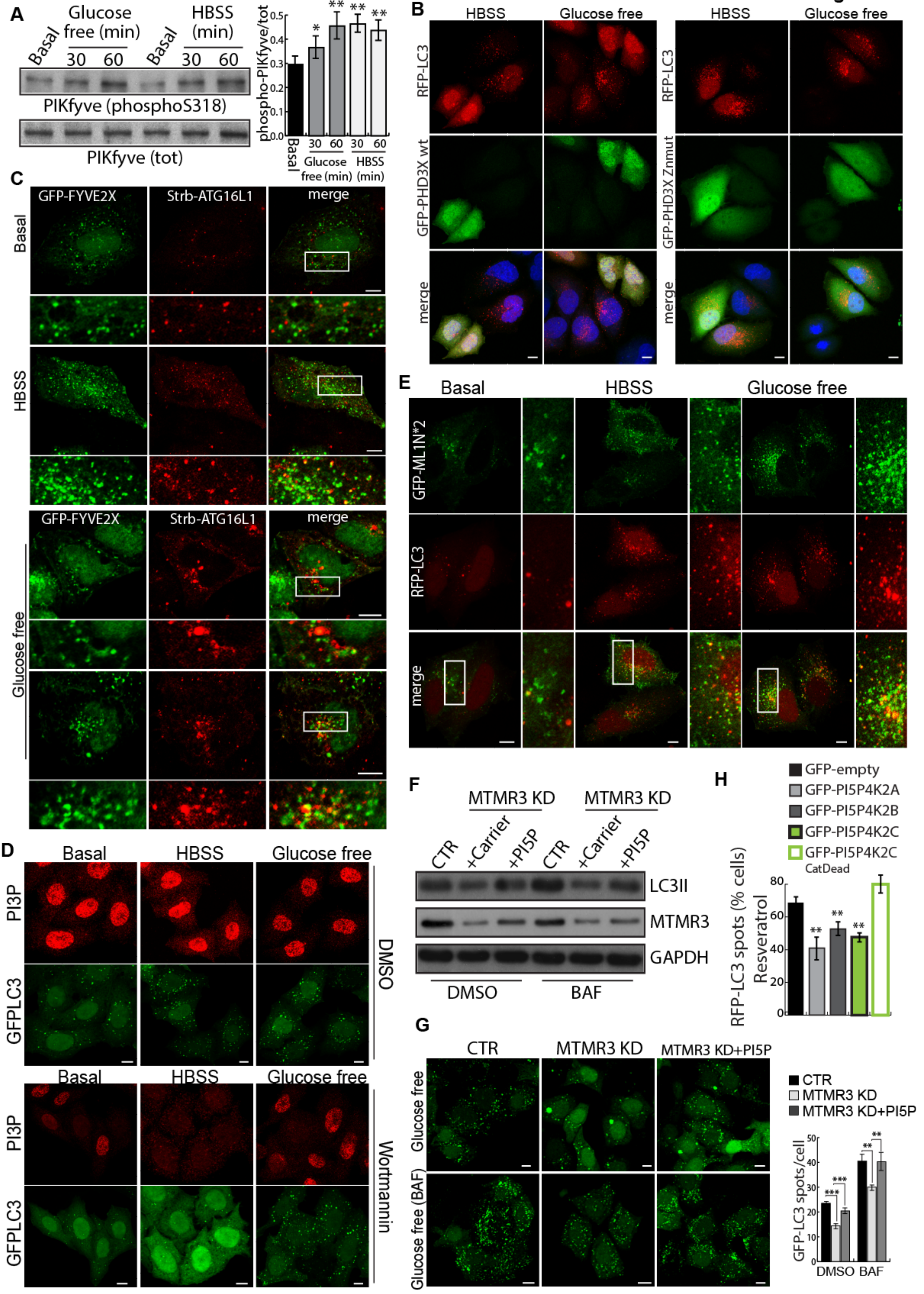
A
Glucose free (min)
HBSS (min)
Basal
30
60
Basal
30
60
PIKfyve (phosphoS318)
PIKfyve (tot)
phospho-PIKfyve/tot
Glucose free (min)
HBSS (min)
B
HBSS
Glucose free
RFP-LC3
GFP-PHD3X wt
GFP-PHD3X Znmut
merge
C
GFP-FYVE2X
Strb-ATG16L1
merge
Basal
HBSS
Glucose free
D
Basal
HBSS
Glucose free
PI3P
GFPLC3
DMSO
Wortmannin
E
Basal
HBSS
Glucose free
GFP-ML1N*2
RFP-LC3
merge
F
MTMR3 KD
CTR
+Carrier
+PI5P
LC3II
MTMR3
GAPDH
DMSO
BAF
G
CTR
MTMR3 KD
MTMR3 KD+PI5P
Glucose free
Glucose free (BAF)
CTR
MTMR3 KD
MTMR3 KD+PI5P
GFP-LC3 spots/cell
DMSO
BAF
H
GFP-empty
GFP-PI5P4K2A
GFP-PI5P4K2B
GFP-PI5P4K2C
GFP-PI5P4K2C CatDead
RFP-LC3 spots (% cells)
Resveratrol

Figure S7: PI(5)P is required for autophagosome formation during glucose starvation, Related to Figure 7. (**A**) MEF cells were incubated for indicated times in glucose free or HBSS media and Western blot analysis of PIKfyve phosphorylation was performed. Quantification of three independent experiments in shown (**$p<0.01$, t-test)**.** (**B**) HeLa cells transfected for 30 h with GFP-PHD3X and GFP-PHD3X Znmut combined with RFP-LC3 then were incubated in glucose-depleted media (Glucose free) and fixed after 4 h. (**C**) HeLa cells transfected with GFP-FYVE (a PI(3)P probe) combined with Strb-ATG16L1 for 16 h were incubated in HBSS or glucose-depleted media (Glucose free) and fixed after 4 h. (**D**) HeLa stably expressing GFP-LC3 were incubated in HBSS or glucose-depleted media (Glucose free), in presence or absence of 200 nM Wm, fixed after 4 h and labelled with PI(3)P antibodies. (**E**) HeLa cells transfected with GFP-ML1N*2 (tandem repeat of N-terminal segment of mucolipin1) and RFP-LC3 for 16 h were left in complete media (basal), starvation media (HBSS) or Glucose free DMEM (Glucose free) for 4h, then fixed and imaged on confocal microscope. Bar 10 μm. (**F**) Human neuroblastoma cells (SKSNH) cells were treated with MTMR3 siRNA for 5 days and loaded with carrier alone or combined with 10 μM PI(5)P in presence or absence of BAF in glucose free media. Western blot analysis of LC3-II, tubulin and MTMR3 is shown. (**G**) HeLa GFP-LC3 cells were treated with MTMR3 siRNA for 5 days and loaded with carrier alone or combined with 10 μM PI(5)P in presence or absence of BAF in glucose-free media. Quantification of numbers of autophagic vesicles per cell (GFP-LC3 vesicles) is shown in the graph (mean±s.e.m.). (**H**) HeLa cells transfected with GFP-PI5P4K2A, 2B, 2C or PI5P4K2C catalytic-dead and RFP-LC3 for 30 h were treated with 64 μM Resveratrol for 3 h and then fixed. Quantification of cells (% of total) showing more than 10 autophagic vesicles (RFP-LC3 vesicles) is shown in the graph. n = 100 cells. (mean±s.e.m.).

Table S1. siRNA oligo sequences used in this study, related to Figure 3, 4, 5, 6, 7, S2, S3, S4, S5, S6, S7.

name	sequence
PIP4K**2A** oligo05	gcccgaugggucuuccguaa
PIP4K**2A** oligo06	gaacaucgacgucuaugga
PIP4K**2A** oligo07	gcauguaccggcuuaaugu
PIP4K**2A** oligo08	gaaaugcacaacauccuga
PIP4K**2B** oligo07	gacaaucaucucuucaaua
PIP4K**2B** oligo08	cccagccgcuuuaaguuua
PIP4K**2B** oligo09	ccucaaggguucuacgguu
PIP4K**2B** oligo10	gccagaaagugaagcuauu
PIP4K**2C** oligo09	gaaccuccgugaucgauuu
PIP4K**2C** oligo10	gcaguaugcuaagcgauuc
PIP4K**2C** oligo11	gaagagagauguggaguuu
PIP4K**2C** oligo11	gaagaaagcagcucaugca
**PIKfyve** oligo16	ucugagccauccugguuua
**PIKfyve** oligo15	gauggacguuggcuggauu
**PIKfyve** oligo14	gagaugaguaugcgcugua
**PIKfyve** oligo13	ggcacaagcuauagcaauu
**MTMR3** oligo06	gaccaaacguggacaguuc
**MTMR3** oligo07	gggcaggcauugagauaca
**MTMR3** oligo08	gcaaaguuaucagguguca
**MTMR3** oligo09	ugaaugccgagauauauuu
**VPS34** oligo09	caccaaagcucaucgacaa
**VPS34** oligo10	auagauagcucccaaauua
**VPS34** oligo11	gaacaacgguuucgcucuu
**VPS34** oligo12	gagauguacuugaacguaa

Supplemental Experimental Procedures

Plasmids. GFP-PHD3X and GFP-PHD3X Znmut were kindly provided by B. Payrastre (Inserm/UPS UMR 1048 - I2MC, Tolouse), GFP-PH-Dok5 by J. A. Nunes (Centre de Recherche en Cancerologie de Marseille), GFP-WIPI2B and GFP-WIPI2D by T. Proikas-Cezanne (Eberhard Karls University Tübingen), GFP-DFCP1 by N.T. Ktistakis (Babraham Institute, Cambridge, England, UK), ATG12-HA and ATG5 by N. Mizushima (The Tokyo Metropolitan Institute of Medical Science Japan), GFP-FYVE(EEA1) by H Stenmark (University of Oslo, Norway), GFP-ML1N*2 by H. Xu (University of Michigan, USA), myc-MTMR3wt and myc-MTMR3C413S by T. Noda (Osaka University) pmStrawberry-ATG16L1,peGFP-httQ74 and FLAG-ATG4BC74A have been described elsewhere (Cadwell et al., 2008; Korolchuk et al., 2011; Moreau et al., 2012), . mGFP-PI5P4K2A, mGFP-PI5P4K2B, mGFP-P5IP4K2C and PI5P4K2C-mGFP (N-term) were generated as previously described (Clarke et al., 2008). A kinase-dead mutant of mGFP-PI5P4K2C was generated by site-directed mutagenesis using the primer pairs 5'-CAGCTGAAGATCATGAAATACAGCCTTCTGCTAGGCATCC-3' (forward) and 5'-CCTAGCAGAAGGCTGTATTTCATGATCTTCAGCTGCACTA-3' (reverse) to convert the conserved ATP-binding residue Asp280 into a lysine. PI5P4K2C(D280K) was expressed as recombinant protein and assayed radiometrically to confirm loss of activity (data not shown). pCMV-PI5P4K2C (untagged) plasmid was from Origene.

WIPI2B FTTG mutants were generated by mutagenesis of the wild-type (FRRG) WIPI2B construct using QuikChange Site-Directed Mutatagenesis kit (Agilent Technologies), the primer sequence was GGACAAAAACTCTTTGAGTTTACGACAGGAGTAAAGAGGTGCGTG as described in(Dooley et al., 2014).

siRNA. ON-TARGETplus SMARTpool siRNA and individual siRNA oligonucleotides against human PI5P4K2A, PI5P4K2B, PI5P4K2C, MTMR3, PIKFyve, hVps34 and non-targeting SMARTpool siRNA (D-001810-04) were purchased from Dharmacon. PI5P4K2C knockdown in MEF cells was carried out using oligo 10+oligo 12 that match sequences in mouse PI5P4K2C. Oligos sequences are listed in Supplementary Table 1.

Cell lines and treatments. Human cervical carcinoma (HeLa) cells, human neuroblastoma (SKNSH) cells, human embryonic kidney (HEK293) cells, African green monkey kidney (COS7) cells, autophagy-related protein 5 (Atg5)-deficient ($Atg5^{-/-}$) and wild-type ($Atg5^{+/+}$) mouse embryonic fibroblast (MEF) cell lines (Kuma et al., 2004), were grown at 37°C in DMEM medium (Sigma) supplemented with 10% FBS, 100 U ml^{-1} penicillin/streptomycin, 2 mM l-glutamine and 1 mM sodium pyruvate (basal media), under 5% CO_2. HeLa cells stably expressing GFP-LC3 and HeLa cells stably expressing GFP–mRFP–LC3 were cultured in basal media supplemented with 500 µg/ml G418 (Sigma) (Kimura et al., 2007) or 200 µg/ml hygromycin B (Sigma), respectively. Stable HeLa cells clones expressing GFP-empty or GFP-WIPI2B were selected and cultured in basal media supplemented with 500 µg/ml G418 (Sigma). Vps34 Flox/Flox MEF were kindly provided by P.Codogno (INSERM U1151-CNRS UMR 8253, Paris). Control Vps34Flox/Flox (Control) and *Vps34-/-* (KO) cells were generated by infecting cells with a lentivirus, produced as previously described (Moffat et al., 2006) carrying or not (control) a catalytically active Cre recombinase. The cells were seeded at 1–2 × 10^5 per well in six-well plates, and transfection was performed with TransIT 2020 Mirus (for DNA) or LipofectAMINE 2000 (for siRNA and double transfections with DNA and siRNA) reagents (Invitrogen), using the manufacturer's protocol. ATG16L1-mStrawberry was transfected at 0.3µg per well, pEGFP-httQ74 at 0.5 µg and the other constructs at 1µg. Cells were transfected for 16 h for localization experiments and 30 h for overexpression experiments. Final siRNA concentrations of 50 or 100nM were used for silencing with two rounds of knockdown for 5 days. In some experiments, cells were starved in Hanks balanced salt solution (HBSS) media (Invitrogen) or treated for 1-4 h with 400 nM or 16 h with 200 nM bafilomycin A (Sigma), for 2-4 h with 200 nM Wortmannin (Sigma), for 1-4 h with different concentrations of YM-201636 (Cayman Chemical) or for 3h with 64 µM Resveratrol (Sigma).

HBSS contains 1g/l of D-glucose; DMEM contains 4.5 g/l glucose; and DMEM without glucose has 0g/l glucose. Glucose starvation was carried by culturing cells in DMEM lacking glucose (Invitrogen, #11966) with 10% dialyzed FBS and glutamine for 4 h.

Preparation of liposomes. Thin films were generated following dissolution of the lipids in a 2:1 (v/v) chloroform/methanol mixture and then dried under a stream of argon gas, while they were rotated. Lipids were mixed at the appropriate ratios to preserve the same net charge on liposomes by controlling the 1-palmitoyl-2-oleoyl-sn-glycero-3-phospho-L-serine (PS) molar fraction. The charges were assumed to be: PS -1; PI(3)P or PI(5)P -3. The final compositions in mole percentage of the different liposome population were: PI(3)P-containing liposomes: 15% of dipalmitoyl phosphatidylinositol 3-phosphate (PI(3)P), 30% PS, 55% of 1-palmitoyl-2-oleoyl-sn-glycero-3-phosphocholine (PC); PI(5)P-containing liposomes: 15% of dipalmitoyl phosphatidylinositol 5-phosphate (PI(5)P), 30% PS, 55% PC; PS-containing liposomes: 75% PS, 25% PC. The films were lyophilized overnight, and the containers were sealed with argon gas to prevent oxidation and stored at −20 °C. Before an experiment, the films were suspended in the lysis buffer used for the lipid binding experiment and vortexed for 1.5 min. The lipid suspension underwent five cycles of freezing and thawing followed by extrusion through polycarbonate membranes with 1 and 0.1 μm diameter pores 21 times to create large unilamellar vesicles.

Western blot analysis. Cells were lysed in Laemmli buffer and protein samples were boiled for 5–7 min at 100°C, separated by SDS-PAGE, transferred onto PVDF membranes, subjected to western blot analysis, and, finally visualized using an ECL enhanced chemiluminescence detection kit (GE Healthcare), or with direct infrared fluorescence detection on an Odyssey Infrared Imaging System. The primary antibodies used include anti- tubulin (1:5,000; Sigma Aldrich), GAPDH (1:3000, Abcam), anti-GFP (1:2000, Clontech), anti-ATG5 (1:5,000; Sigma Aldrich), anti-ATG12 (1:1000, Cell Signaling), anti-LC3 (1:4,000; Novus Biologicals), anti-HA (1:1000, Covance), anti-p62 (1:1000, MBL), anti-PI5P4K2A (1:500, Abcam), anti-PI5P4K2B (1:500, Abcam), anti-PIKfyve and anti-phospho-PIKfyve (1:500, Abcam), anti-MTMR3 (1:500, Cell Signaling), anti-VPS34 (1:1000, Cell Signaling).

Immunofluorescence. Cells were grown on coverslips at 25% confluency were fixed in 4% paraformaldehyde (for endogenous WIP1-2, 1:100 Abcam, for myc tag, 1:500 Abcam, for flag tag, 1:500 Sigma Aldrich) or methanol (for endogenous LC3, 1:100 Novus Biologicals and anti-GFP, 1:400 Abcam) for 5 min and then permeabilized with 0.1% Triton. 1% BSA in PBS was used for blocking and primary and secondary antibodies. A Zeiss LSM710 confocal microscope was used for fluorescent confocal analysis. All confocal images were taken with a 63X oil-immersion lens. ImageJ (number of vesicles analysis) were used for further analysis and processing of confocal images.

Staining of PI(3)P for immunofluorescence

The staining of PtdIns(3)P was performed as described by Hammond et al. (2009). Briefly, cells were fixed in 2% paraformaldehyde and permeabilized with 20 μM digitonin in buffer A (20 mM Pipes pH 6.8, 137 mM NaCl, 2.7 mM KCl). Then cells were blocked with buffer A supplemented with 5% (v/v) FBS and 50 mM NH4Cl. Anti-PtdIns(3)P antibodies from Echelon (for 1 h, 1:300) and secondary antibodies were applied in buffer A with 5% FBS. Cells underwent post-fixation for 5 min in 2% paraformaldehyde, washed with PBS containing 50 mM NH4Cl, washed once with water and then mounted with Mowiol.

TIRF microscopy. HeLa cells were seeded on 35-mm MatTek glass-bottomed Petri dishes at a density of about 1.5×10^5 cells per dish. Cells were transfected for 16 h with relevant reagents, after which they were cultured in HBSS for a further 2 h, and treated with 100 nM YM-201636 for the last 1 h and fixed. Imaging was performed on a Zeiss TIRF 3 system using an αPlan-Fluor 100× 1.45 numerical aperture oil-immersion lens under the control of the AxioVision software. Laser lines at 488 and 561 nm were used for GFP–PHD3X and Strawberry-ATG16L1 excitation, respectively.

Electron microscopy. HeLa cells were loaded for 2 h with carrier alone, or combined with 10 μM PI(5)P, in presence of 200 nM Wm in HBSS and then fixed with a mixture of 2% paraformaldehyde and 2% glutaraldehyde in cacodylate buffer 0.1M (pH. 7.4) for 1 h, at room temperature. Cells were then post-fixed in Osmium tetroxide 1% in cacodylate buffer 0.1M (pH. 7.4) and processed for Epon embedding. Epon

sections were cut using REICHERT ultracut S and analysed with Philips CM100 electron microscope. Morphometrical analysis was performed counting autophagosome structures in 40 random cells profile for each condition in two independent experiments.

Autophagy analyses. Measurement methods for the levels of endogenous LC3-II/tubulin ratios as a read-out for autophagosome numbers have been previously described (Sarkar et al., 2009). To quantify LC3-positive vesicles, cells were transfected with an RFP-LC3 plasmid or were fixed and stained with an anti-LC3II antibody. We determined the percentages of cells with more than 10 LC3-positive vesicles. All experiments were carried out in triplicate with at least 200 cells counted per slide; the scorer was blinded to treatment. Automated microscope counting of total autophagosomes (in HeLa stable expressing GFP-LC3) or autolysosomes labelled with a pH-sensitive mRFP-GFP-LC3 (also called tfLC3) was carried out using a Thermo Scientific Cellomics ArrayScan VTI HCS reader and the Spot Detector Bioapplication protocol, as described (Sarkar et al., 2009). With tfLC3, GFP- (and mRFP-) positive puncta represent autophagosomes before lysosomal fusion, whereas mRFP-positive puncta (that lack GFP fluorescence) represent autolysosomes (as the GFP is more rapidly quenched by the low pH(Kimura et al., 2007)). At least 1000 cells per condition in three independent experiments were used for quantification. Alternatively, total numbers of GFP-RFP and RFP-only positive puncta per cell were quantified from confocal images using ImageJ and the Analyse Particles plugin (a constant threshold for all of the images within each experiment was applied). At least 20 cells per condition in three independent experiments were used for quantification.

ATG5-ATG12 conjugation was detected in cells transfected with HA-ATG12 and ATG5, lysed and subjected to western blot analysis with anti-HA antibody, so we could assess free ATG12 and the ATG5-ATG12 complex formed by the transfected constructs. We determined the ratio of ATG5-ATG12 versus free ATG12.

EGFP-httQ74 aggregation was detected by direct immunofluorescence. The proportion of transfected cells with aggregates was scored (approx. 500 cells per coverslip). Experiments were performed blinded and in triplicate in at least three independent experiments. Statistics for aggregation assays were calculated as odds ratios (the ratio of cells containing aggregates in each condition).

References

Cadwell, K., Liu, J.Y., Brown, S.L., Miyoshi, H., Loh, J., Lennerz, J.K., Kishi, C., Kc, W., Carrero, J.A., Hunt, S., *et al.* (2008). A key role for autophagy and the autophagy gene Atg16l1 in mouse and human intestinal Paneth cells. Nature *456*, 259-263.

Clarke, J.H., Emson, P.C., and Irvine, R.F. (2008). Localization of phosphatidylinositol phosphate kinase IIgamma in kidney to a membrane trafficking compartment within specialized cells of the nephron. Am J Physiol Renal Physiol *295*, F1422-1430.

Dooley, H.C., Razi, M., Polson, H.E., Girardin, S.E., Wilson, M.I., and Tooze, S.A. (2014). WIPI2 links LC3 conjugation with PI3P, autophagosome formation, and pathogen clearance by recruiting Atg12-5-16L1. Mol Cell *55*, 238-252.

Hammond, G.R., Schiavo, G., and Irvine, R.F. (2009). Immunocytochemical techniques reveal multiple, distinct cellular pools of PtdIns4P and PtdIns(4,5)P(2). Biochem J *422*, 23-35.

Kimura, S., Noda, T., and Yoshimori, T. (2007). Dissection of the autophagosome maturation process by a novel reporter protein, tandem fluorescent-tagged LC3. Autophagy *3*, 452-460.

Korolchuk, V.I., Saiki, S., Lichtenberg, M., Siddiqi, F.H., Roberts, E.A., Imarisio, S., Jahreiss, L., Sarkar, S., Futter, M., Menzies, F.M., *et al.* (2011). Lysosomal positioning coordinates cellular nutrient responses. Nat Cell Biol *13*, 453-460.

Kuma, A., Hatano, M., Matsui, M., Yamamoto, A., Nakaya, H., Yoshimori, T., Ohsumi, Y., Tokuhisa, T., and Mizushima, N. (2004). The role of autophagy during the early neonatal starvation period. Nature *432*, 1032-1036.

Moffat, J., Grueneberg, D.A., Yang, X., Kim, S.Y., Kloepfer, A.M., Hinkle, G., Piqani, B., Eisenhaure, T.M., Luo, B., Grenier, J.K., *et al.* (2006). A lentiviral RNAi library for human and mouse genes applied to an arrayed viral high-content screen. Cell *124*, 1283-1298.

Moreau, K., Ravikumar, B., Puri, C., and Rubinsztein, D.C. (2012). Arf6 promotes autophagosome formation via effects on phosphatidylinositol 4,5-bisphosphate and phospholipase D. J Cell Biol *196*, 483-496.
